# Hidden diversity in the Brazilian Atlantic rainforest: the discovery of Jurasaidae, a new beetle family (Coleoptera, Elateroidea) with neotenic females

**DOI:** 10.1038/s41598-020-58416-6

**Published:** 2020-01-31

**Authors:** Simone Policena Rosa, Cleide Costa, Katja Kramp, Robin Kundrata

**Affiliations:** 1 Universidade Federal de Itajubá, Instituto de Recursos Naturais, Av. BPS, 1303, 37500-903 Itajubá, MG Brazil; 2 Museu de Zoologia, Universidade de São Paulo, Avenida Nazaré, 481, 04263-000 São Paulo, SP Brazil; 3 Senckenberg Deutsches Entomologisches Institut, Eberswalder Strasse 90, 15374 Müncheberg, Germany; 4 Department of Zoology, Faculty of Science, Palacky University, 17. listopadu 50, 771 46 Olomouc, Czech Republic

**Keywords:** Ecology, Evolution, Zoology

## Abstract

Beetles are the most species-rich animal radiation and are among the historically most intensively studied insect groups. Consequently, the vast majority of their higher-level taxa had already been described about a century ago. In the 21st century, thus far, only three beetle families have been described *de novo* based on newly collected material. Here, we report the discovery of a completely new lineage of soft-bodied neotenic beetles from the Brazilian Atlantic rainforest, which is one of the most diverse and also most endangered biomes on the planet. We identified three species in two genera, which differ in morphology of all life stages and exhibit different degrees of neoteny in females. We provide a formal description of this lineage for which we propose the new family Jurasaidae. Molecular phylogeny recovered Jurasaidae within the basal grade in Elateroidea, sister to the well-sclerotized rare click beetles, Cerophytidae. This placement is supported by several larval characters including the modified mouthparts. The discovery of a new beetle family, which is due to the limited dispersal capability and cryptic lifestyle of its wingless females bound to long-term stable habitats, highlights the importance of the Brazilian Atlantic rainforest as a top priority area for nature conservation.

## Introduction

Coleoptera (beetles) is by far the largest insect order by number of described species. Approximately 400,000 species have been described, and many new ones are still frequently being discovered even in regions with historically high collecting activity^1^. Because this group has been intensively studied since the age of early systematists, and most of the major higher-level taxa had already been described by about a century ago, discoveries of new beetle families are nowadays a rare occurrence. The vast majority of recent family descriptions is based on the already known taxa for which a new systematic placement was recovered by the use of phylogenetic analyses^2–5^. Thus, only three beetle families have been erected *de novo* in the 21st century based on newly collected material. Firstly, Aspidytidae were described based on two species from South Africa and China, which currently represent monotypic genera^6,7^. Secondly, the monotypic family Meruidae was created for a hitherto undescribed lineage from Venezuela^8^. Finally, the new monogeneric family Iberobaeniidae was described based on three species from the southern part of the Iberian Peninsula^9,10^. Whereas both Aspidytidae and Meruidae represent semiaquatic families within the suborder Adephaga, Iberobaeniidae were recovered as a deep branch in the polyphagan superfamily Elateroidea^9^.

Elateroidea (the click-beetles, soldier-beetles, fireflies and relatives), the largest superfamily of the series Elateriformia, form one of the major and oldest polyphagan lineages^11,12^. Currently, they contain approximately 25,000 described species classified into 13 families^13–16^. The elateroid beetles exhibit extraordinary morphological diversity. Some lineages contain representatives with a fully sclerotized, compact body and presence of a defensive pro-mesothoracic clicking mechanism, while other groups are soft-bodied, with loosely connected abdominal segments and often with variously modified or reduced body parts^13,17^. Such morphological modifications are a result of neoteny, a phenomenon in which animals retain larval characters while attaining reproductive maturity^18–20^. In Elateroidea, females are usually much more affected by neoteny and exhibit various degrees of incomplete metamorphosis; from possession of a larva-like abdomen only, through having a larva-like body excluding the head, to a completely larviform body^16,21–23^. Since developmental modifications lead to similar morphological traits, these groups were usually considered a monophylum (i.e., former Cantharoidea)^17,21^. However, recent molecular-based research showed the multiple origin of soft-bodiedness and neoteny in Elateroidea^9,13,16,22,23^.

**Figure 1:**
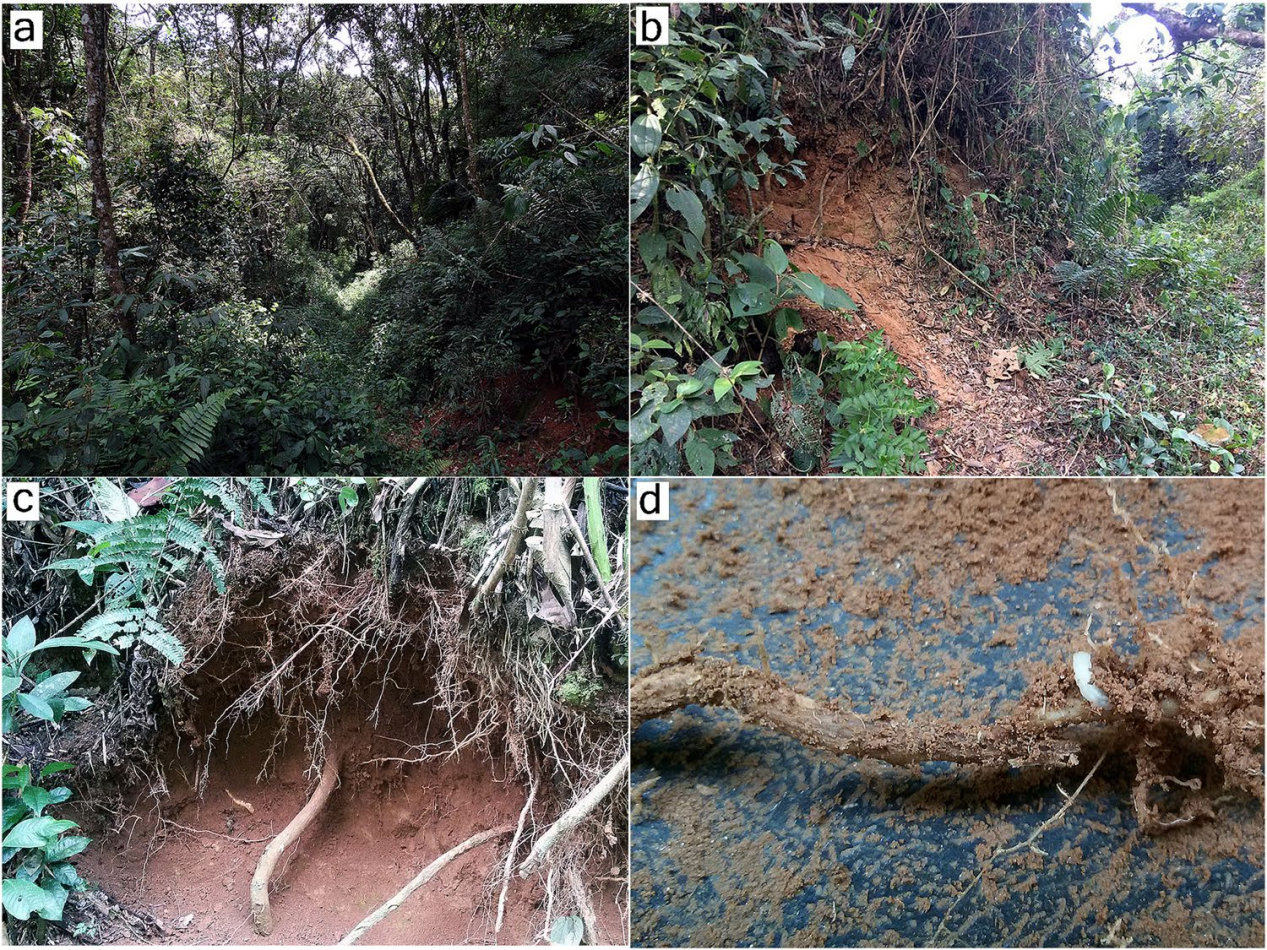
Habitat of *Jurasai itajubense* gen. et sp. nov. and *Tujamita plenalatum* gen. et sp. nov. in the Biological Reserve of Serra dos Toledos: (**a**) main trail; (**b**) ravine along the main trail; (**c**) soil with roots where most larvae were collected; (**d**) larva of *J. itajubense* near the root.

Here, we report the discovery of a completely new elateroid neotenic lineage from the Brazilian Atlantic forest ecoregion, which is one of the Earth’s most endangered biodiversity hotspots with high levels of diversity and endemism^24,25^. The story of a discovery began on 24 May 2016, when an expedition of the first author (SPR) and her undergraduate students was organized to the Municipal Biological Reserve Serra dos Toledos in the Serra da Mantiqueira mountain chain as part of research on its Lampyridae fauna^26^. During that expedition, the first two larvae of the here described new elateroid lineage were collected in soil of a ravine along the main trail of the reserve (Fig. 1). Based on their bizzare morphology, including the remarkable mouthparts which resembled a beak, collectors were not able to assign the specimens to any known beetle family, although it was obvious they belong to Elateroidea. A few weeks later the larvae metamorphosed into pupae and later to a soft-bodied adult male and a larviform female. Because they represented an apparently undescribed elateroid lineage, a great effort was made to collect more larvae during every expedition to Serra dos Toledos. In 2017, adult males of a second species were found among samples from Malaise traps in Serra dos Toledos, and Luiz F. L. Silveira (Western Carolina University, USA) sent us adult males of a third species collected using Malaise traps in the Serra dos Órgãos National Park, a part of the Serra do Mar mountain chain. In this study, we conducted a detailed evaluation of the morphological characters of all life stages and investigated the phylogenetic position of the newly discovered lineage using a multi-marker molecular phylogeny. All available evidence demonstrates the need to establish a new family within the Elateroidea for three new species in two new genera.

## Results

Based on their divergent morphology (Figs. 2–5, S1–S12) and the results obtained from the molecular phylogeny (Figs. 6, S13), we describe here *Jurasai digitusdei* gen. et sp. nov., *J. itajubense* gen. et sp. nov. and *Tujamita plenalatum* gen. et sp. nov., for which we erect a new family Jurasaidae fam. nov. Below we provide remarks on their biology, ecology and behavior, as well as concise diagnostic descriptions for all new taxa based primarily on salient diagnostic characters. Detailed morphological diagnoses and descriptions are given in the Supplementary Text.

**Figure 2:**
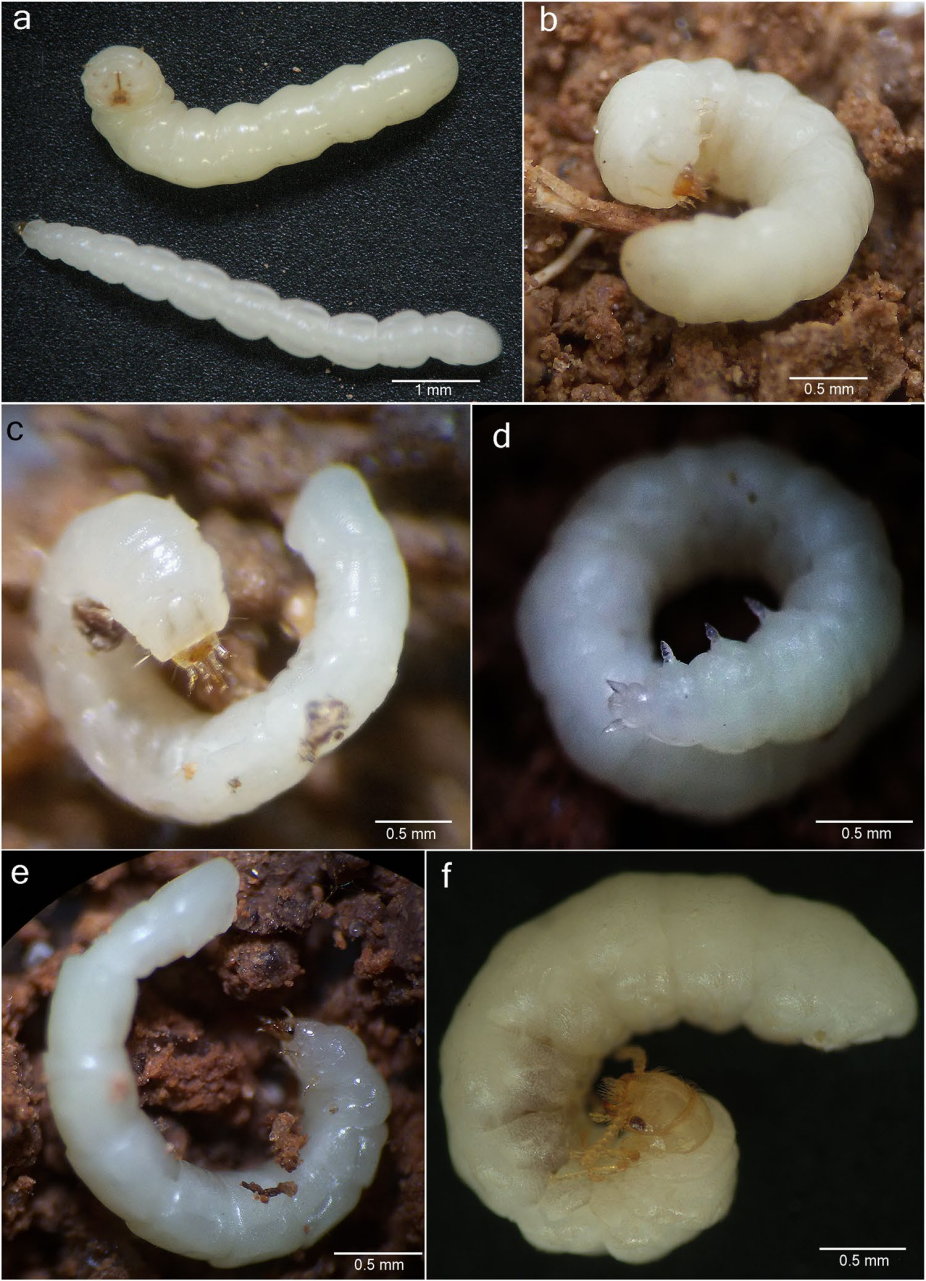
Live specimens of Jurasaidae fam. nov. (**a**) larvae of *Tujamita plenalatum* gen. et sp. nov. (upper specimen) and *Jurasai itajubense* gen. et sp. nov. (lower specimen); (**b**) larva of *T. plenalatum*; (**c–e**) *J. itajubense*: larva, female pupa and adult female, respectively; (**f**) adult female of *T. plenalatum*.

**Figure 3:**
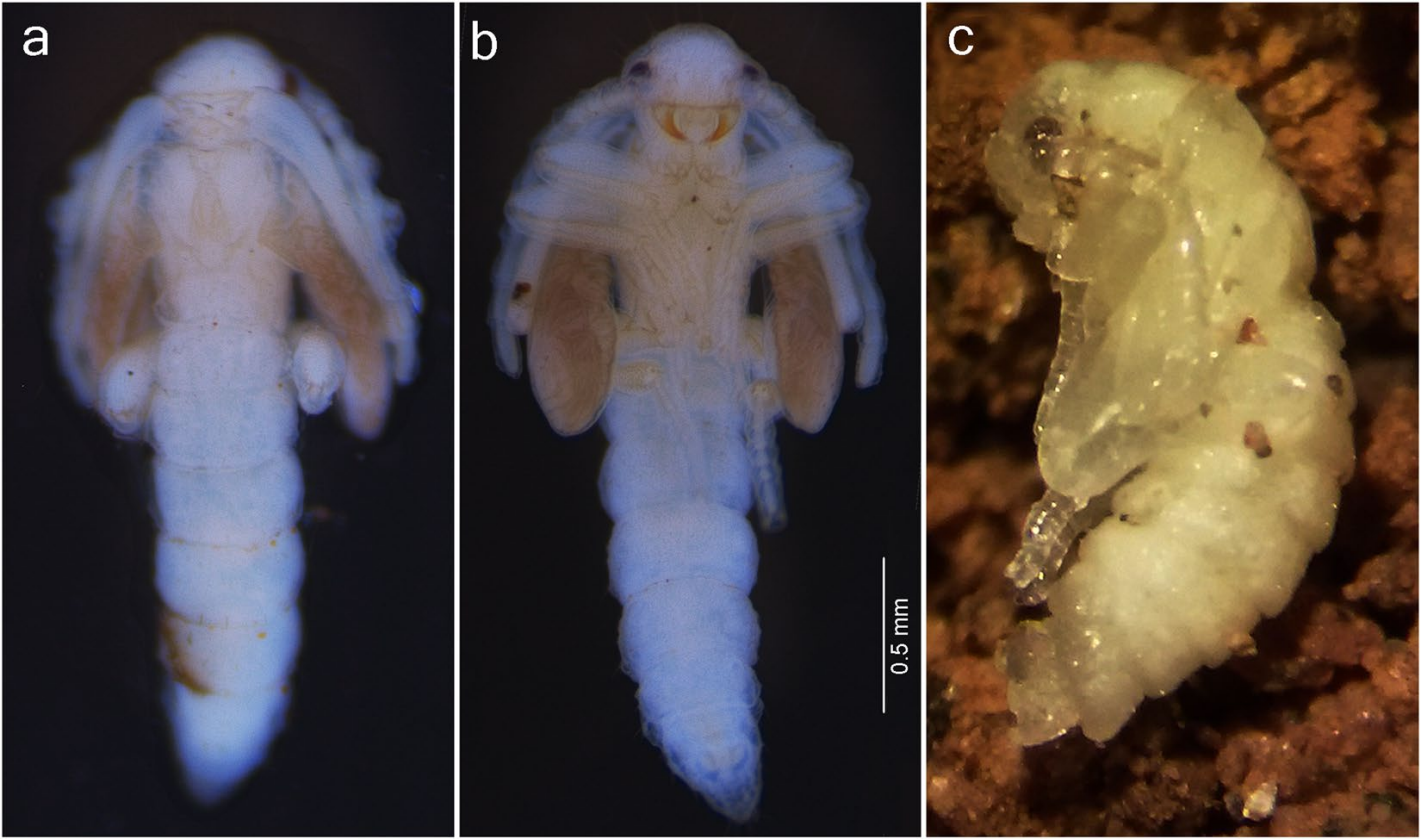
Male pupae of Jurasaidae fam. nov. (**a,b**) *Jurasai itajubense* gen. et sp. nov., dorsal and ventral view, respectively; (**c**) *Tujamita plenalatum* gen. et sp. nov., lateral view.

**Figure 4:**
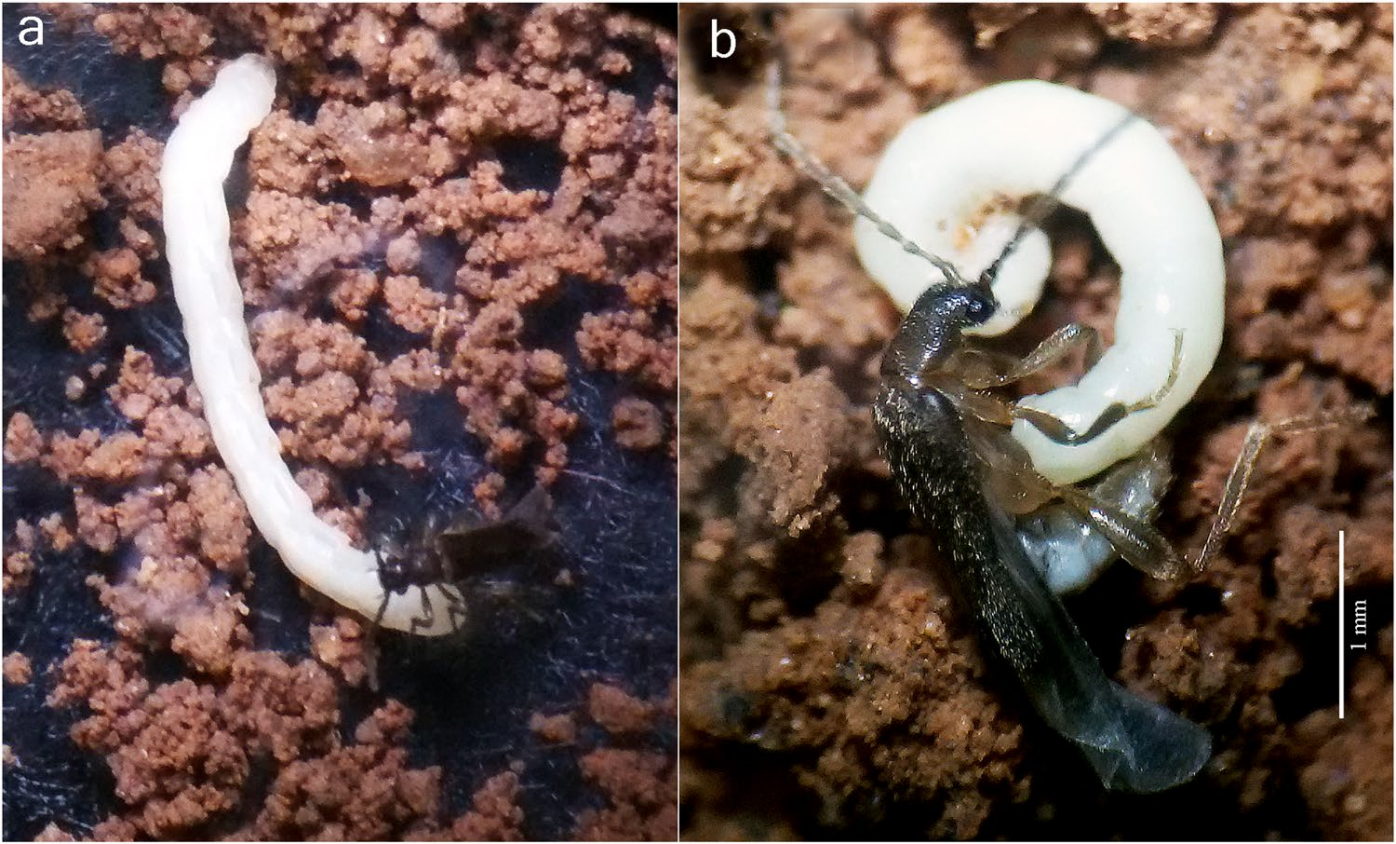
Mating behavior of *Jurasai itajubense* gen. et sp. nov. (**a**) male touches a female with mouthparts; (**b**) male touches a female with aedeagus.

**Figure 5:**
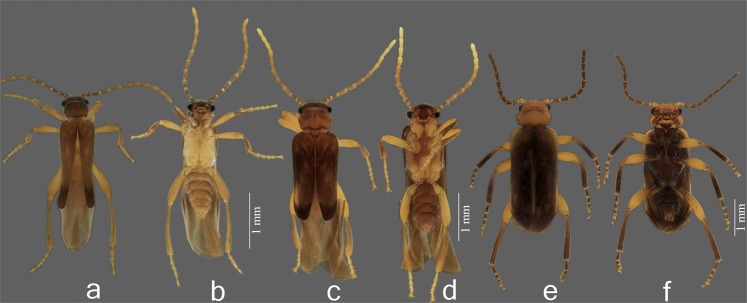
Adult males of Jurasaidae fam. nov. (**a,b**) *Jurasai itajubense* gen. et sp. nov., dorsal and ventral view, respectively; (**c,d**) *J. digitusdei* gen. et sp. nov., dorsal and ventral view, respectively; (**e,f**) *Tujamita plenalatum* gen. et sp. nov., dorsal and ventral view, respectively.

**Figure 6:**
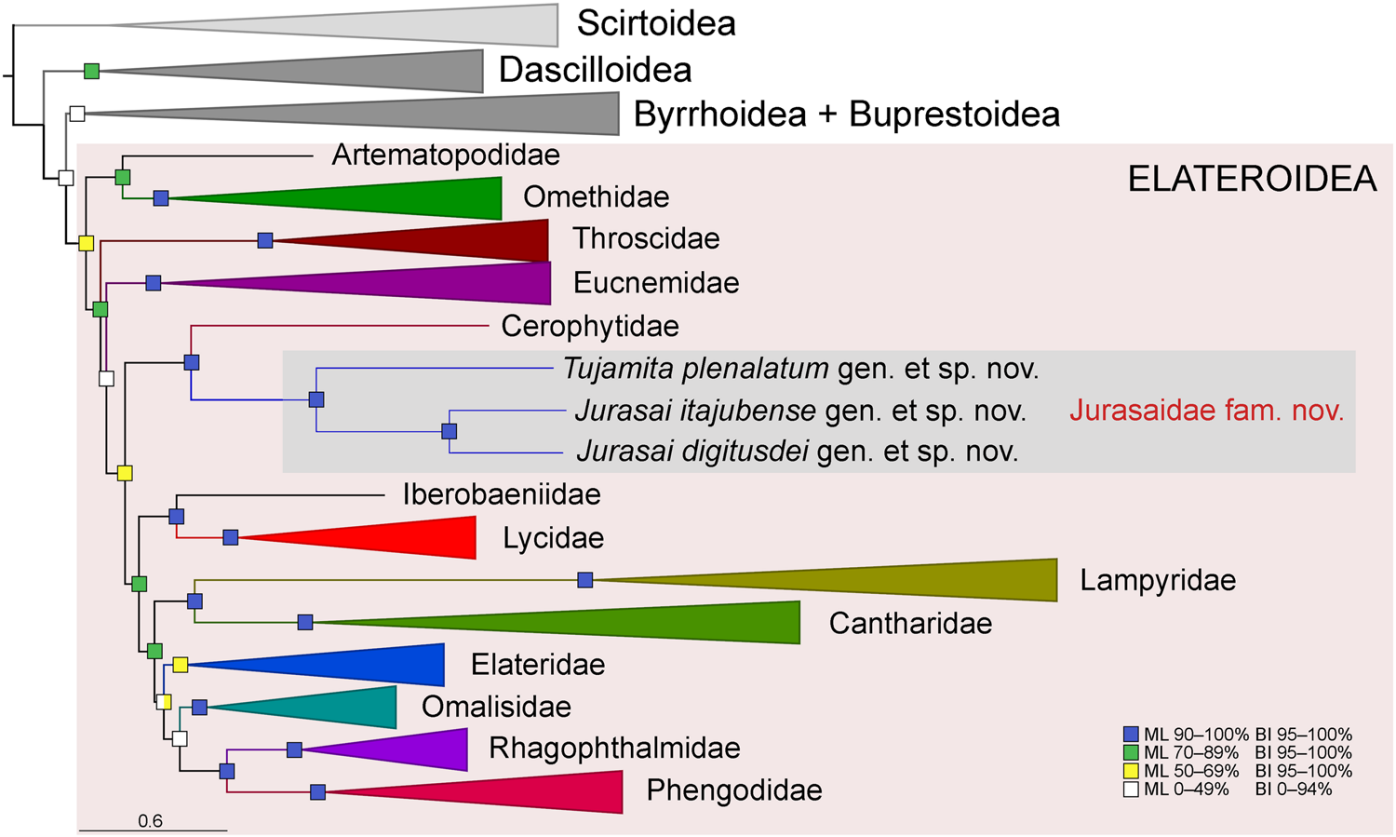
Phylogenetic position of Jurasaidae within Elateriformia based on the maximum likelihood (ML) analysis performed on the four-gene 251-taxa dataset aligned using the MAFFT algorithm. Statistical support for branches includes bootstrap values for the ML analysis and posterior probabilities for the Bayesian inference (BI). The full-resolution tree with taxon names is given in Fig. S13.

### Observations of behavior of the immature stages and adults

Immature stages of two species (out of three) representing two different genera are known to date. Larvae and pupae of *J. itajubense* and *T. plenalatum* occur sympatrically in the Municipal Biological Reserve Serra dos Toledos. They are usually found in the 5–20-cm-thick layer of soil just under the leaf litter (Figs. 1c, 2a–d, 3). Although both species share the same microhabitat, *T. plenalatum* is much more rare; of the total 87 larvae collected, only four belonged to that species. The feeding habits of both larvae and adults are not known. Despite our offering the larvae various potential food items in the laboratory, no movement of the mouthparts or signs of pumping into the esophagus were observed. However, since many larvae were found between roots, we hypothesize that they feed on juices of fungal hyphae. Both larvae and adults usually stay in the soil. When unearthed and disturbed they become active and soon bury themselves again into the substrate. Bioluminescence is present in several elateroid lineages in Brazil, i.e., Lampyridae (fireflies), Phengodidae (glowworms) and Elateridae: Pyrophorini (luminous click-beetles)^22,27,28^. However, it was not detected in Jurasaidae, neither by the naked eye nor by using long exposure photography. In both species, the pupal stage lasts 22–24 days in males and 8–9 days in females. Whilst a male pupa can move only its abdomen, a female pupa is capable of walking, although it usually stays burrowed in the substrate or resting on the surface in a C-shaped position (Fig. 2d). The adult female is wingless and resembles a larva in general appearance, with only certain parts of the head adult-like (*Jurasai*, Fig. 2e) or with the head, prothorax and legs adult-like, though different in appearance from the corresponding parts in the male (*Tujamita*, Fig. 2f) (Table S1). About a week after eclosion, the male actively searches for a female and then copulates several times each for a short period of time (Fig. 4, Supplementary Video). More detailed information on field and laboratory observations are given in the Supplementary Text.

### Systematics

#### Jurasai gen. nov

(Figs. 1d, 2a,c–e, 3a,b, 4, 5a–d, S1–S4, S6a–i, S8a,b, S10a–i, S11a,b, S12a–h).

*Type species*. *Jurasai itajubense* sp. nov.; by present designation.

*Diagnostic description*. **Adult male** (Figs. 4, 5a–d, S1, S2a–h, S4, S6a–i, S8a,b). Body length from frons to apex of abdomen 2.5–3.0 mm, from frons to apex of wing 3.5–5.0 mm. Head with antenna approximately as long as distance between head and elytral apex; labrum 1.8–4.0 times as wide as long; maxillary palpus 4- or 5-segmented; apical labial palpomere 2–5 times longer than basal palpomere. Pronotum (Figs. S2b, S4b,d, S6e) widest at anterior half, posterior half narrowed posteriad; without lateral carinae; posterior edge smooth, not marginated; mesoventrite (Figs. S4f–h, S6e) 1.1 times longer than wide, with anterior margin deeply arcuate; mesepimeron with anterior part indistinct in lateral view, separated from mesanepisternum by weakly impressed suture; mesocoxal cavities separated at middle by 1.3 times mesocoxal cavity width; metaventrite (Fig. S4f) 1.3 times longer than wide, 2.5 times longer than mesoventrite, widest at anterior 1/3. Elytra (Figs. 5a,c, S1a,f) shorter than abdomen, tapered apically, with median edges separated and divergent apicad, lateral edges sinuate, apices swollen; hind wing (Fig. S8a,b) surpassing elytral apex by 0.5–0.7 times elytral length, apical field long (0.5–0.6 times as long as total wing length). Tarsomere IV (Fig. S1e) evenly sclerotized, truncate apically. Abdomen (Fig. S1h) narrow, with sides subparallel; phallus and parameres (Figs. S2f,g, S4m–o) together 1.6 times wider than long; endophallus emerging from dorso-apical elongate notch. **Adult female** (Figs. 2e, 4, S2i,j, S3) (based on *J. itajubense* only). Abdomen and thorax almost equal to those of larvae; head with a pair of pigmented stemmata; mandible falcate; labrum free; maxilla and labium separated, labium not channeled to fit mandibles; antenna with four antennomeres; leg with tibia and tarsus separated, tarsus 1-1-1, with a pair of claws. **Mature larva** (Figs. 1d, 2a,c, S10a–i, S11a,b, S12a–h) (based on *J. itajubense* only). Body 4–7 mm long, slender, 9–10 times longer than wide; clypeolabrum (Fig. S12c) with two long setae. Mandibles and labium (Fig. S10b) forming elongate and sharpened beak-like mouthpart; mandible (Figs. S11b, S12e,f) with apex gradually sharpened apicad, parallel-sided, base elliptical; hypopharyngeal bracon hyaline. Pronotum (Fig. S12a) with four setae between lateral edge and lateral sclerotized stripe (one pair anteriorly and one pair posteriorly), two setae between stripes anteriorly; mesothorax, metathorax, and abdominal segments I–VIII (Fig. S12a,b) with one pair of laterodorsal setae near anterior margin, one pair of laterodorsal setae near posterior margin, and a pair of lateroventral setae near posterior margin. Legs (Fig. S10g) separated by 5–6 times diameter of coxa.

*Etymology*. From Tupi-Guarani language; Jura = mouth; saí = minuscule, thin; allusion to the

larval mouth. Gender: neuter.

*Composition and distribution*. *Jurasai itajubense* (Brazil: Minas Gerais) and *J. digitusdei* (Brazil: Rio de Janeiro).

#### Jurasai itajubense sp. nov

(Figs. 1d, 2a,c–e, 3a,b, 4, 5a,b, S1a–e, S2, S3, S6a–e, S8a, S10a–i, S11a,b, S12a–h).

*Type material*. Holotype, male, “Brazil, Minas Gerais state, Itajubá municipality, Biological Reserve of Serra dos Toledos (22°25′21.3″S 45°22′06.2″W), 1,358 m, soil ravine, collected as pupa on 7.VI.2018 (adult on 5.VII.2018, died on 8.VIII.2018), Rosa S.P, Barbosa T. & Paiva J. leg.” (deposited at MZUSP). For information on paratypes and other material examined see the Supplementary Text.

*Diagnostic description*. Adult male (Fig. 5a,b). Labrum with anterior margin emarginate; maxillary palpus 4-segmented; elytra strongly tapered; hind wing venation with only RA_1+2_ and MP_1+2_ veins; abdominal sternite VIII partly exposed; parameres with apices tapered and curved inwards. For more details see the generic diagnostic description and the Supplementary Text.

*Etymology*. From the type locality, Itajubá, in Minas Gerais state, Brazil.

#### Jurasai digitusdei sp. nov

(Figs. 5c,d, S1f–i, S4, S6f–i, S8b).

*Type material*. Holotype, male, “Brazil, Rio de Janeiro State: Teresópolis, Parque Nacional da Serra dos Órgãos, malaise trap, PVE 6B (22°28′11″S 43° 0′5.3″W, 868 m), VI.2015, Silveira & Khattar leg.” (deposited at DZRJ). For information on paratypes and other material examined see the Supplementary Text.

*Diagnostic description*. Adult male (Fig. 5c,d). Labrum with anterior margin rounded; maxillary palpus 5-segmented; elytra weakly tapered; hind wing venation with RA_1+2_, R_3_, RA_3+4_, RM loop and medial field veins; abdominal sternite VIII concealed; parameres with apices sausage-like, directed posteroventrad. For more details see the generic diagnostic description and the Supplementary Text.

*Etymology*. From Latin; digitus = finger, dei = of god; allusion to “Dedo de Deus”, a mountain peak near the type locality in Serra dos Órgãos National Park, whose shape resembles a hand pointing up towards the sky.

#### Tujamita gen. nov

(Figs. 2a,b,f, 3c, 5e,f, S5, S6j–l, S7, S8c, S9, S10j–m, S11c–e, S12i–l).

*Type species*. *Tujamita plenalatum* sp. nov.; by present designation.

*Diagnostic description*. **Adult male** (Figs. 5e,f, S5, S6j–l, S7, S8c). Body length from frons to apex of abdomen 3.1–4.5 mm, from frons to apex of wing 3.2–4.6 mm. Head with antenna approximately 2/3 as long as distance between head and elytral apex; labrum four times as wide as long; maxillary palpus 5-segmented; apical labial palpomere 6–8 times longer than basal palpomere. Pronotum (Figs. S5d, S6j) weakly narrowed posteriad, with lateral margins subparallel and carinate; posterior edge marginated; mesoventrite (Figs. S6k, S7f–h) 1.3 times wider than long, with anterior margin weakly arcuate; mesepimeron with anterior part distinct in lateral view, separated from mesanepisternum by grooved suture; mesocoxal cavities separated at middle by 0.7 times mesocoxal cavity width; metaventrite (Fig. S7g) 1.1 times wider than long, 2.8–2.9 times longer than mesoventrite, widest at midlength. Elytra (Figs. 5e, S5a) slightly shorter or as long as abdomen, parallel-sided, median edges contiguous to apex; apices flat. Hind wing (Fig. S8c) surpassing elytral apex by 0.2 times elytral length, apical field short (0.3–0.4 times as long as total wing length). Tarsomere IV (Fig. S5e) deeply notched. Abdomen (Fig. S5f) wide, with sides rounded and tapered apicad; phallus and parameres (Fig. S7o,p) together 1.1 times wider than long; endophallus emerging from apical oval orifice on dorsal surface of phallus. **Adult female** (Figs. 2f, S9). Meso-, metathorax and abdomen almost equal to those of larva; head, pronotum and leg (Fig. S9a–g) adult-like but different from those of male: compound eyes very small, flat, not protruded; posterior tentorial pits absent; antenna moniliform, with nine antennomeres; pronotal lateral carinae absent, prosternum reduced to very narrow sclerotized strip; tarsi 4-4-4. **Mature larva** (Figs. 2a,b, S10j–m, S11c–e, S12i–l). Body 4–6 mm long, stout, 5–6 times longer than wide; clypeolabrum (Figs S10j, S12i) with four long setae; mandibles and labium (Figs. S10j,l, S11c–e) forming short, stout, beak-like mouthpart; mandible (Figs. S11d, S12l) with apex abruptly sharpened apically, convergent anteriad, base triangular; hypopharyngeal bracon sclerotized. Pronotum with five setae between lateral edge and lateral stripe (one pair anteriorly, one pair posteriorly, and single seta at midlength), four setae between stripes (one pair anteriorly and one pair posteriorly); mesothorax, metathorax, and abdominal segments I–VIII with six setae at midlength (one pair lateral, one pair laterodorsal and one pair parasagittal); ventral surface of abdominal segments I–VIII with six setae at midlength (two pairs ventrolateral and one pair parasagittal). Legs separated by 8–12 times diameter of coxa.

*Etymology*. From Tupi-Guarani language; Tuja = adult, mitã = child; allusion to neoteny. Gender: neuter.

*Composition and distribution*. Only *T. plenalatum* (Brazil: Minas Gerais).

#### Tujamita plenalatum sp. nov

*Type material*. Holotype, male, “Brazil, Minas Gerais state, Itajubá municipality, Municipal Biological Reserve of Serra dos Toledos (22°25′21.3″S 45°22′06.2″W), 1,358 m, malaise trap, 15.X.–8.XI.2015, Rosa S.P. & Dias D. leg.” (deposited at MZUSP). For information on paratypes and other material examined see the Supplementary Text.

*Diagnostic description*. Adult male (Fig. 5e,f). Labrum four times as wide as long, anterior margin rounded; maxillary palpus 5-segmented; pronotum with lateral carina; elytra not tapered apicad, apices contiguous; hind wing 0.2 times as long as elytra; abdomen relatively wide, with sides rounded and tapered apically; phallus and parameres together 1.1 times as wide as long; endophallus emerging from dorso-apical oval orifice on dorsal surface of phallus. For more details see the generic diagnostic description and the Supplementary Text.

*Etymology*. From Latin; plenus = full, alatus = winged; allusion to complete elytra.

#### Jurasaidae fam. nov

(Figs. 2–5, S1–S12).

*Type genus*. *Jurasai* gen. nov.

*Diagnostic description*. **Male**. Body soft (Fig. 5); head (Figs. S2a, S4a, S5d, S6a–c,f,g, S7a,b,d) declivous, frontoclypeal suture absent, labrum sclerotized, free, separated from head capsule by membrane; antennal insertions elevated and visible from above; antenna filiform, with 11 antennomeres. Mandible falcate, unidentate; maxillary cardo, stipes, galea and lacinia indistinct, last palpomere shorter than all remaining palpomeres combined; gular sutures separated; two posterior tentorial pits present. Thorax (Figs. S2b, S4b–h, S5d, S6e,j,k, S7f–h) with pronotum wider than long, narrower than elytral base; prosternum keel-shaped, strongly convex medially, prosternal process not extending beyond coxae; procoxal cavities open internally and externally, widely separated by prosternal process; mesoventrite evenly sclerotized with anterior margin arcuate, separated from metaventrite by feeble suture; leg (Figs. S1e,g, S5b,e) with meso- and metacoxae oblique, pro- and mesotrochantin exposed; procoxa, mesocoxa and mesal part of metacoxa conical and strongly projecting, tibial spurs long, tarsi 5-5-5, without lamellae or pulvilli. Elytra (Figs. 5, S1a,f, S5a) soft, irregularly punctate, epipleura gradually narrowed posteriorly; wings (Fig. S8) folded longitudinally in resting position, longer than elytra, hiding the abdomen in dorsal view, veins reduced or blurred, cells and transverse cross veins absent, apical field with three triangular sclerotizations and a notch between two most apical ones. Abdomen (Figs. S1h, S4h, S5f) with five free ventrites (i.e., sternites III–VII); sternites II and VIII largely membranous and concealed; sternite IX (Figs. S2e, S4l, S5g, S7m) with apex bilobed, densely pilose and projecting above phallus and parameres; punctures surrounded by usually 2–3 campaniform sensilla in a dog’s-paw pattern (Fig. S1i). Aedeagus (Figs. S2f–h, S4m–o, S5g, S7o–q) trilobate, not entirely retractable into abdomen, phallobase 2.0–2.7 times as long as wide, 1.4–2.6 times as long as parameres, sheathed by a tubular membrane which opens anteriorly into a pair of large balloon-like membranous vesicles; parameres and phallus together 1.1–1.6 times wider than long, basal struts of phallus absent; endophallus emerging from dorso-apical opening, flagellum absent. **Female** (Fig. 2e,f). Body elongate, wingless, with varying degree of neoteny, but always with at least meso-/metathorax and abdomen larva-like (except for ooporus in posterior margin of sternite VIII) (Fig. S3j,k) (Table S1); leg short, with paired claws; ooporus with pair of membranous lobe-like valves, each with supporting sclerotized plate. **Larva** (Figs. 1d, 2a–c, S10, S11, S12a–f, i–l). Body cream or milky-white, cylindrical, with few setae; head sclerotized, wedge-like; epicranial sutures and endocarina absent; clypeolabrum triangular, translucent; gula, maxillae and labium fused to each other and to head capsule ventrally, anterior part of labium projected with pair of channels dorsally, fitting apical half of strongly sharpened mandibles, forming a beak-like mouthpart; basal half of mandibles elongate and retracted into anterior 2/3 of head, linked to inner sclerotized rod that extends into prothorax; pronotum with pair of parasagittal sclerotized stripes; prosternum with strongly sclerotized median longitudinal rod; leg short, 5-segmented; trochanter ring-shaped, pretarsus glabrous.

*Composition*. *Jurasai* (two species) and *Tujamita* (monotypic).

*Distribution*. Brazil (Minas Gerais, Rio de Janeiro).

### Phylogenetic analyses

All phylogenetic analyses placed Jurasaidae in Elateroidea and showed an identical backbone topology for the superfamily, with the main clades branching off in the following order: Artematopodidae + Omethidae (incl. Telegeusinae), Throscidae, Eucnemidae, Cerophytidae + Jurasaidae, and the terminal clade of “higher elateroids” sensu Kundrata *et al*.^13^ (i.e., Lycidae, Iberobaeniidae, Lampyridae, Cantharidae, Elateridae, Omalisidae, Phengodidae and Rhagophthalmidae). The maximum likelihood (ML) phylogenetic tree of 251 terminals with collapsed branches and Elateroidea families highlighted is given in Fig. 6; the full-resolution tree is in Fig. S13. Jurasaidae were always placed as a sister group of *Cerophytum elateroides* (Cerophytidae), with 90–95% bootstrap support in the ML analyses and 100% posterior probabilities in the Bayesian (BI) analyses. This clade was sister to the “higher elateroids” with robust statistical support in the BI analyses and weaker support in the ML analyses (Figs. 6, S13). Jurasaidae formed a maximally supported monophylum, with *Tujamita* sister to a clade formed by two species of *Jurasai*. We associated the different developmental stages and sexes using molecular markers to confirm the field and laboratory observations (Fig. S14). The uncorrected pairwise genetic distance between *cox1* sequences for *J. itajubense* and *T. plenalatum* was 28.5% (*cox1* was not available for *J. digitusdei*).

## Discussion

The majority of the currently recognized families of Elateroidea was described more than a hundred years ago, and only recent fieldwork resulted in surprising discoveries of Iberobaeniidae in southern Spain^9^ and the here reported Jurasaidae in southeastern Brazil. These families had not been discovered earlier most probably due to a minute, soft body in adult males, neotenic, wingless females with a presumably short lifespan (only hypothesized in Iberobaeniidae), and a cryptic lifestyle. Jurasaidae males superficially resemble other small soft-bodied elateroids, with which they share the weakly sclerotized cuticle, widely separated and protruding eyes, partially reduced ventral mouthparts, falcate mandibles, prosternum in front of coxae very short and with incomplete prosternal process, widely open procoxae, conspicuous prothoracic tubular spiracles, conical and projecting coxae, exposed pro- and mesotrochantins, absent elytral costae, relatively shortened elytra compared to a flexible abdomen, hind wings with reduced venation and folded only longitudinally, and the abdomen with free ventrites connected by visible membranes (Table S2; Figs. 5, S1a–c,f–h, S4a–h, S5a–c,d,f, S8). Jurasaidae differ from all other Elateroidea in males by having the following autapomorphies: the ventral surface of the thorax and abdomen with punctures surrounded by usually 2–3 campaniform sensilla in a dog’s-paw pattern (Figs. S1i, S2c, S7j), apex of abdominal sternite IX with a pair of elongate lobes projecting above the parameres and phallus (Fig. S7n), aedeagus which is not retracted into the abdomen, and the phallobase sheathed by a tubular membrane which opens anteriorly into a pair of large membranous vesicles (Figs. S4m–o, S5g, S7o–q). The neotenic females differ from other elateroids in having an ooporus located on the posterior margin of sternite VIII, with a pair of membranous lobe-like valves, each one with a supporting sclerotized plate (Fig. S3j,k), and the larvae are characterized by the following unique characters: fused ventral mouthparts (i.e., maxillae and labium), labial channels located dorsally, and the basal half of the mandibles retracted deeply into the head capsule and linked to an inner sclerotized rod that extends into the prothorax (Figs. S10b,d,e, S11, S12c–f,i–l). Apart from the above-mentioned unique morphological features, Jurasaidae differ from each of the other soft-bodied elateroid groups in the Neotropics, i.e., Phengodidae, Lampyridae, Cantharidae, Lycidae, and Omethidae: Telegeusinae, by a number of additional features (see the Supplementary Text).

Due to the parallel origins of soft-bodiedness, body miniaturization and the characters connected with prematurely terminated metamorphosis in Elateroidea^9,14,16,22,23,29^, a detailed phylogenetic placement of Jurasaidae based on morphology alone would be precarious. Indeed, a preliminary examination of *J. itajubense* led the discoverers of this species to place it tentatively in Penicillophorini, a small group of uncertain position, either in Phengodidae or Omethidae: Telegeusinae^30,31^. However, our detailed morphological investigation revealed the considerable differences between Jurasaidae and Penicillophorini (see the Supplementary Text) and the molecular phylogenetic analyses placed Jurasaidae unambiguously to basal elateroid splits as a sister group of Cerophytidae, far from Phengodidae, Telegeusinae and other soft-bodied lineages (Figs. 6, S13). Although Jurasaidae and Cerophytidae adults are superficially different (i.e., both sexes in Cerophytidae are completely metamorphosed, with a fully sclerotized body and functional clicking mechanism), their larvae share the cylindrical, white and grub-like body, very small wedge-shaped head without dorsal and ventral epicranial sutures, non-opposable, flattened and channeled mandibles, labium with channels to fit the mandibular apices, prosternum with a median sclerotized endocarinate rod, and short legs. Remarkably, many of these features are also shared with Eucnemidae and Throscidae, and were used to define a monophyletic group formed by Eucnemidae, Throscidae and Cerophytidae^32,33^. On the other hand, the labial channels which fit the mandibular apices are an unambiguous synapomorphy of Cerophytidae + Jurasaidae. Since the phylogenetic relationships among the basal elateroids including the above-mentioned families have not yet been satisfactorily resolved (and this is also beyond the scope of this paper), more effort should be made to test the monophyly of Eucnemidae, Throscidae, Cerophytidae and Jurasaidae using more robust datasets and analytical approaches^11–13,32,33^. This would be crucial for understanding the evolution of modified larval morphology associated with the adaptation to burrowing in soil or rotten wood and feeding on juices of fungal hyphae.

The discovery of Jurasaidae and their placement in the basal Elateroidea shed new light on the evolution of neoteny in this beetle superfamily. Neoteny in Elateroidea has long been a widely studied phenomenon^19,21^, and recent studies repeatedly confirmed that it originated several times not only within the superfamily but also within several distantly related families^14,20,22,23,29^. Elateroidea are divided into the “basal grade” with mostly hard-bodied groups with only Omethidae and Jurasaidae having a soft body, and the robustly supported clade of “higher elateroids” which contains predominantly fully sclerotized click-beetles and the vast majority of soft-bodied lineages^13^. It is hypothesized that soft-bodiedness in both adult sexes is the initial stage of ontogenetic modifications leading to development of highly modified larviform females^20^. Until now, the neotenic females with variously modified morphology were known almost exclusively within terminal higher elateroids, and Omethidae: Telegeusinae were the only basal lineage with unknown but supposedly neotenic females. Jurasaidae represent the second loss of sclerotization among basal elateroid lineages, and are the first proven case of highly modified neotenic females outside the terminal clade of higher elateroids. Interestingly, the females of both genera in Jurasaidae exhibit different degrees of neoteny, with *Jurasai* being almost completely larviform and *Tujamita* having the head, prothorax and legs partly adult-like, although more reduced compared to an adult male (Supplementary Text, Table S1). Similar cases of different levels of morphological modifications in females of different genera were reported also for Lampyridae^19^, Lycidae^20^, Rhagophthalmidae^34^ and Elateridae^23^. Not only females, but also males in Jurasaidae, present interesting morphological modifications connected with soft-bodiedness and neoteny. In Elateroidea, divergence in the abdominal morphology represents a continuous scale from five ventrites of which all or at least some are connate (the majority of the well-sclerotized elateroids) through various intermediate stages to seven or eight ventrites, which are all free and connected by extensive intersegmental membranes (majority of the soft-bodied lineages)^29^. Jurasaidae males represent an intermediate form; their abdomen is soft and with five free ventrites (Figs. S1h, S5f). A similar condition is only known in the otherwise morphologically dissimilar Brachypsectridae. Moreover, similar to females, *Jurasai* males are morphologically more affected by ontogenetic modifications than *Tujamita* males, having a pronotum without strengthening structures (pronotum with lateral carinae in *Tujamita*), elytra with median edges divergent posteriorly and with separately tapered apices (elytra parallel-sided, with sutural edges contiguous to apex in *Tujamita*), and with the abdomen narrower and more exposed (abdomen wider and shorter, usually covered by elytra in *Tujamita*) (Figs. 5, S1h, S5f, S6e,k). Finally, the two species of *Jurasai* exhibit different degrees of morphological modifications, with *J. itajubense* being more neotenic than *J. digitusdei*, having a reduced number of maxillary palpomeres, less compact and relatively narrower elytra, and much more reduced hind wing venation (Figs. 5a–d, S1a,b,f,g, S6c,g, S8a,b). Our present findings therefore represent additional evidence for the complex and gradual morphological modifications caused by the various degrees of incomplete metamorphosis in elateroid beetles.

The neotenic elateroid beetles are known for their extremely low dispersal ability, limited geographic ranges, and strong dependence on long-term climatically stable habitats including the humid tropics^9,20,35,36^. These attributes make neotenics excellent indicators of the long, uninterrupted evolutionary history of tropical forests. The Brazilian Atlantic rainforest is a complex biome formed by a network of ecosystems without clear limits, covering approximately 3,000 km of the eastern coast of Brazil^37^. It hosts one of the world’s most diverse tropical forest biotas with an exceptionally high level of species endemism^38,39^ but due to human activities leading to the intense landscape transformation during the last centuries, it is considered one of the most endangered biomes and belongs among the “hottest biodiversity hotspots” on the planet^24,40^. The region currently remains in constant degradation, mostly fragmented into isolated archipelagos of small forest fragments surrounded by open-habitat matrices, with only less than 10% of its original coverage^25,41,42^ and only about a third of its total extent preserved by conservation units^24^. Nevertheless, even those remaining forest fragments still harbor a multitude of newly discovered animal taxa including both vertebrates^43–45^ and invertebrates^46–51^. Currently, the largest and best preserved portions of the Brazilian Atlantic rainforest are situated near the southern mountain ranges including Serras do Mar and da Mantiqueira^42^, where all Jurasaidae known thus far were collected. Such a late discovery of a new beetle family, which is due to the cryptic lifestyle of its representatives bound to these ancient, stable habitats, further stresses the importance of the Brazilian Atlantic rainforest as a top priority area for nature conservation.

## Material and Methods

### Taxon sampling and collecting sites

A total of 120 specimens (21 adult males, 8 adult females, 4 pupae, 87 larvae) from two localities in the Brazilian Atlantic forest ecoregion in southeastern Brazil were studied (Supplementary Text). Larvae, pupae, adult males and females of the first two species were collected in the soil or using Malaise traps in the Serra dos Toledos reserve, and adult males of the third species were collected using Malaise traps in the Serra dos Órgãos National Park. For the detailed numbers of individuals under each species, corresponding localities and information on the collecting and rearing of specimens see the Supplementary Text. The Serra dos Toledos reserve is situated in the municipality of Itajubá, Minas Gerais state in the Mantiqueira mountain range. It comprises an area of 10.7 km^2^ with altitudes ranging from 1,100 to 1,800 m. The reserve was originally covered by dense ombrophilous and *Araucaria* forests, but nowadays it includes secondary forests and suffers from the deforestation caused by the surrounding agricultural and livestock activities^52^. The Serra dos Órgãos National Park is situated in the municipality of Teresópolis, Rio de Janeiro state in the Serra do Mar mountain range. It covers an area of 105.3 km^2^ with altitudes ranging from 200 to 2,285 m. This conservation unit comprises one of the largest area of dense ombrophilous vegetation in Rio de Janeiro state, although approximately 45% are under anthropogenic land use, mainly pastures. Both Serra dos Toledos and Serra dos Órgãos are situated in the Atlantic Rainforest domain^48,53^. The collection, maintenance, and shipping of specimens were conducted in accordance with the Brazilian environmental laws (Sisbio permits 53842-1, 53842-2, 53842-3, 43943-1, SisGen Shipping Registration R1CE59F, and authorization of the Municipal Government of Itajubá).

### Laboratory methods

Whole genomic DNA was extracted using the E.Z.N.A Tissue DNA Kit (Omega Bio-tek Inc. Norcross, USA) following standard protocol but with the overnight incubation and elution performed twice with 100 µl Elution buffer each. Amplifications were performed using Qiagen Multiplex PCR Plus Master Kit (Qiagen, Hilden, Germany) according to the manufacturer protocols. The PCR amplification conditions and list of primers used were reported in previous study^54^. We sequenced two nuclear and two mitochondrial markers which have been widely used for Coleoptera and Elateriformia phylogenies^9,13,22,55,56^, i.e., 18S rRNA (1842–1843 bp), 28S rRNA (627–629 bp), *rrnL* (505 bp), and *cox1* mtDNA (723 bp). Selection of these markers, which are abundantly represented in GenBank, enabled us to test the position of the newly discovered lineage using extensive outgroups. The PCR products were purified using Exonuclease I and FastAP Thermosensitive Alkaline Phosphatase (Life Technologies, Darmstadt, Germany), and subsequently sent for sequencing to the commercial facility run by Macrogen Europe, Netherlands.

### Dataset assembly and phylogenetic analyses

In addition to the field and laboratory observations, we associated the different developmental stages and sexes using the sequences from 10 specimens (three adult males, two adult females, one pupa, four larvae) representing three morphologically defined species from two localities (Table S3). We used the alignment method and the maximum likelihood analysis as described below.

To investigate the position of the newly discovered lineage, we merged the sequences of individuals from the three newly discovered species (one per species) with the 251-taxon dataset from the most comprehensive phylogenetic study of Elateriformia to date^56^. Scirtiformia were used as an outgroup, since they represent basal Polyphaga^11,12^. Only terminals for which all four markers were present were included, except for a few taxonomically important lineages for which some fragments were missing (Table S3). Forward and reverse sequences were edited and assembled using Geneious 7.1.7^57^ and multiple sequence alignments were generated using MAFFT 7.157^58^ at default parameters. Alignment of the length invariable protein-coding *cox1* sequences was checked by amino acid translation. Basic statistics and the pairwise sequence divergences based on uncorrected p-distance were calculated using MEGA 6.06^59^. The resulting alignment included 4780 homologous positions (2338, 1128, 591 and 723 positions for 18 S, 28 S, *rrnL* and *cox1*, respectively), and contained 2134 conserved, 2408 variable, and 1872 parsimony informative characters.

Model and best-fit partitioning testing was carried out using a greedy algorithm in PartitionFinder 1.1.1^60^ under the corrected Akaike information criterion. Subsequent phylogenetic trees were generated using maximum likelihood analysis (ML) and Bayesian inference (BI). ML was conducted using RAxML 8.2.9^61,62^ with the default settings, partitioned by genes and codons, and bootstrapped with 1000 pseudoreplicates. BI was performed using MrBayes 3.2.6^63^ with the GTR + I + G model for most partitions (SYM + I + G for 28 S rRNA) and partitioning by genes and codon positions as recommended by PartitionFinder. Four chains were run for 40 million generations using the Markov chain Monte Carlo method. Stationary phase and convergence were checked using Tracer 1.7.1^64^ and the first 20% of generations were discarded as burn-in. The posterior probabilities (PP) were calculated from the remaining trees. All analyses were run on the CIPRES web server^65^.

We also evaluated the occurrence of substitution saturation in our data using Xia’s nucleotide substitution saturation test^66^ implemented in DAMBE 5.6.14^67^. We analyzed separately each non-coding gene and each position of the protein-coding *cox1* mtDNA using 10,000 replicates on the fully resolved sites and with the empirical proportion of invariant sites estimated from the data. Only little saturation was detected in our data except for the 3rd codon positions of the *cox1* fragment, which were substantially saturated (Table S4). Therefore, we also performed the above-described ML and BI analyses with discarded third codon positions. Additionally, to test the effect of extensive outgroup on the alignment accuracy, we removed from the dataset all terminals other than Elateroidea and Byrrhidae (outgroup), re-aligned the sequences of individual markers, and re-analyzed the concatenated dataset of 95 terminals using both ML and BI analyses.

### Morphology

Male specimens were soaked in 10% KOH for 24 hours before dissection; female abdomens were boiled in KOH for dissection of genital tract; larvae soaked in KOH were extensively damaged, so they were dissected without treatment. Specimens and body parts were mounted in slides with Hoyer’s medium and examined under a stereomicroscope and compound microscope. Live specimens were also examined under a microscope to examine chaetotaxy and mouthparts. Drawings were made using a Zeiss Discovery V8 stereomicroscope with an attached camera lucida; photos were taken with a Canon A640 digital camera and a Axiocam camera attached to a Zeiss Imager A1 microscope. For examination with the scanning electron microscope, the specimens were rinsed with soapy water, gradually dehydrated in graded ethanol (70%, 80%, 90%, 99%), dried in a critical point dryer with liquid CO_2_ (Bal Tec SCD-050), mounted on aluminum stubs with double-sided conductive tape, and sputtered with gold. Images were taken using a Zeiss LEO 440 scanning electron microscope. Stacked photographs were combined using Helicon Focus (Helicon Soft Ltd., Ukraine). Body width of the specimens was measured at the widest part of the body, pronotal length at midline, and pronotal width at the widest part. The examined material is deposited at the Universidade Federal de Itajubá (UNIFEI), Museu de Zoologia da Universidade de São Paulo (MZUSP), the collection of Prof. J. A. P. Dutra at the Universidade Federal do Rio de Janeiro (DZRJ), and the collection of Palacky University, Olomouc, Czech Republic (UPOL). Morphological terminology follows Lawrence *et al*.^68^, and the classification of Elateroidea follows Kundrata *et al*.^13^, with the subsequent changes made by other authors^9,14,15,30^.

## Supplementary information

Supplementary Information.

Supplementary Information.

## Data Availability

All newly generated sequences have been deposited in GenBank under the accession numbers MN578248–MN578256 and MN583350–MN583375 (Table S3). The datasets analyzed during the current study are available from the corresponding author on request.
