# Author Correction: Hidden diversity in the Brazilian Atlantic rainforest: the discovery of Jurasaidae, a new beetle family (Coleoptera, Elateroidea) with neotenic females

**DOI:** 10.1038/s41598-020-60629-8

**Published:** 2020-02-25

**Authors:** Simone Policena Rosa, Cleide Costa, Katja Kramp, Robin Kundrata

**Affiliations:** 1 Universidade Federal de Itajubá, Instituto de Recursos Naturais, Av. BPS, 1303, 37500-903 Itajubá, MG Brazil; 2 Museu de Zoologia, Universidade de São Paulo, Avenida Nazaré, 481, 04263-000 São Paulo, SP Brazil; 3 Senckenberg Deutsches Entomologisches Institut, Eberswalder Strasse 90, 15374 Müncheberg, Germany; 4 Department of Zoology, Faculty of Science, Palacky University, 17. listopadu 50, 771 46 Olomouc, Czech Republic

Correction to: *Scientific Reports* 10.1038/s41598-020-58416-6, published online 31 January 2020

This Article does not include ZooBank LSID number, which is the requirement of the International Commission on Zoological Nomenclature (ICZN). This issue is addressed with this Correction.

The LSID for this publication is: urn:lsid:zoobank.org:pub:C7ACEBA1-9A88-489C-9FC2-1D446387423A. Below, we give the ZooBank LSID numbers for each of the described taxa, along with the Systematics section of the original article.

***Systematics***

*Jurasai gen. nov*.; ZooBank LSID: urn:lsid:zoobank.org:act:1A85CFF4-A7DC-4FFA-8283-0849DAD5EAFC.

*Type species*. *Jurasai itajubense* sp. nov.; by present designation.

*Diagnostic description*. **Adult male**. Body length from frons to apex of abdomen 2.5–3.0 mm, from frons to apex of wing 3.5–5.0 mm. Head with antenna approximately as long as distance between head and elytral apex; labrum 1.8–4.0 times as wide as long; maxillary palpus 4- or 5-segmented; apical labial palpomere 2–5 times longer than basal palpomere. Pronotum widest at anterior half, posterior half narrowed posteriad; without lateral carinae; posterior edge smooth, not marginated; mesoventrite 1.1 times longer than wide, with anterior margin deeply arcuate; mesepimeron with anterior part indistinct in lateral view, separated from mesanepisternum by weakly impressed suture; mesocoxal cavities separated at middle by 1.3 times mesocoxal cavity width; metaventrite 1.3 times longer than wide, 2.5 times longer than mesoventrite, widest at anterior 1/3. Elytra shorter than abdomen, tapered apically, with median edges separated and divergent apicad, lateral edges sinuate, apices swollen; hind wing surpassing elytral apex by 0.5–0.7 times elytral length, apical field long (0.5–0.6 times as long as total wing length). Tarsomere IV evenly sclerotized, truncate apically. Abdomen narrow, with sides subparallel; phallus and parameres together 1.6 times wider than long; endophallus emerging from dorso-apical elongate notch. **Adult female** (based on *J. itajubense* only). Abdomen and thorax almost equal to those of larvae; head with a pair of pigmented stemmata; mandible falcate; labrum free; maxilla and labium separated, labium not channeled to fit mandibles; antenna with four antennomeres; leg with tibia and tarsus separated, tarsus 1-1-1, with a pair of claws. **Mature larva** (based on *J. itajubense* only). Body 4–7 mm long, slender, 9–10 times longer than wide; clypeolabrum with two long setae. Mandibles and labium forming elongate and sharpened beak-like mouthpart; mandible with apex gradually sharpened apicad, parallel-sided, base elliptical; hypopharyngeal bracon hyaline. Pronotum with four setae between lateral edge and lateral sclerotized stripe (one pair anteriorly and one pair posteriorly), two setae between stripes anteriorly; mesothorax, metathorax, and abdominal segments I–VIII with one pair of laterodorsal setae near anterior margin, one pair of laterodorsal setae near posterior margin, and a pair of lateroventral setae near posterior margin. Legs separated by 5–6 times diameter of coxa.

*Etymology*. From Tupi-Guarani language; Jura = mouth; saí = minuscule, thin; allusion to the larval mouth. Gender: neuter.

*Composition and distribution*. *Jurasai itajubense* (Brazil: Minas Gerais) and *J. digitusdei* (Brazil: Rio de Janeiro).

*Jurasai itajubense* sp. nov.; ZooBank LSID: urn:lsid:zoobank.org:act:C8E9861F-C41E-45BB-9ADB-C6E53D904D37.

*Type material*. Holotype, male, “Brazil, Minas Gerais state, Itajubá municipality, Biological Reserve of Serra dos Toledos (22 °25′21.3″S 45 °22′06.2″W), 1,358 m, soil ravine, collected as pupa on 7.VI.2018 (adult on 5.VII.2018, died on 8.VIII.2018), Rosa S.P, Barbosa T. & Paiva J. leg.” (deposited at Museu de Zoologia da Universidade de São Paulo). For information on paratypes and other material examined see the original Article.

*Diagnostic description*. Adult male. Labrum with anterior margin emarginate; maxillary palpus 4-segmented; elytra strongly tapered; hind wing venation with only RA_1+2_ and MP_1+2_ veins; abdominal sternite VIII partly exposed; parameres with apices tapered and curved inwards. For more details see the original Article.

*Etymology*. From the type locality, Itajubá, in Minas Gerais state, Brazil.

*Jurasai digitusdei* sp. nov.; ZooBank LSID: urn:lsid:zoobank.org:act:91291FDF-2F4C-4C80-A792-9BFFAD1ACBFE.

*Type material*. Holotype, male, “Brazil, Rio de Janeiro State: Teresópolis, Parque Nacional da Serra dos Órgãos, malaise trap, PVE 6B (22 °28′11″S 43° 0′5.3″W, 868 m), VI.2015, Silveira & Khattar leg.” (deposited in the collection of Prof. J. A. P. Dutra at the Universidade Federal do Rio de Janeiro). For information on paratypes and other material examined see the original Article.

*Diagnostic description*. Adult male. Labrum with anterior margin rounded; maxillary palpus 5-segmented; elytra weakly tapered; hind wing venation with RA_1+2_, R_3_, RA_3+4_, RM loop and medial field veins; abdominal sternite VIII concealed; parameres with apices sausage-like, directed posteroventrad. For more details see the original Article.

*Etymology*. From Latin; digitus = finger, dei = of god; allusion to “Dedo de Deus”, a mountain peak near the type locality in Serra dos Órgãos National Park, whose shape resembles a hand pointing up towards the sky.

*Tujamita* gen. nov.; ZooBank LSID: urn:lsid:zoobank.org:act:A8A670EF-E16D-444D-89B0-48F05FBFCFE6.

*Type species*. *Tujamita plenalatum* sp. nov.; by present designation.

*Diagnostic description*. **Adult male**. Body length from frons to apex of abdomen 3.1–4.5 mm, from frons to apex of wing 3.2–4.6 mm. Head with antenna approximately 2/3 as long as distance between head and elytral apex; labrum four times as wide as long; maxillary palpus 5-segmented; apical labial palpomere 6–8 times longer than basal palpomere. Pronotum weakly narrowed posteriad, with lateral margins subparallel and carinate; posterior edge marginated; mesoventrite 1.3 times wider than long, with anterior margin weakly arcuate; mesepimeron with anterior part distinct in lateral view, separated from mesanepisternum by grooved suture; mesocoxal cavities separated at middle by 0.7 times mesocoxal cavity width; metaventrite 1.1 times wider than long, 2.8–2.9 times longer than mesoventrite, widest at midlength. Elytra slightly shorter or as long as abdomen, parallel-sided, median edges contiguous to apex; apices flat. Hind wing surpassing elytral apex by 0.2 times elytral length, apical field short (0.3–0.4 times as long as total wing length). Tarsomere IV deeply notched. Abdomen wide, with sides rounded and tapered apicad; phallus and parameres together 1.1 times wider than long; endophallus emerging from apical oval orifice on dorsal surface of phallus. **Adult female**. Meso-, metathorax and abdomen almost equal to those of larva; head, pronotum and leg adult-like but different from those of male: compound eyes very small, flat, not protruded; posterior tentorial pits absent; antenna moniliform, with nine antennomeres; pronotal lateral carinae absent, prosternum reduced to very narrow sclerotized strip; tarsi 4-4-4. **Mature larva**. Body 4–6 mm long, stout, 5–6 times longer than wide; clypeolabrum with four long setae; mandibles and labium forming short, stout, beak-like mouthpart; mandible with apex abruptly sharpened apically, convergent anteriad, base triangular; hypopharyngeal bracon sclerotized. Pronotum with five setae between lateral edge and lateral stripe (one pair anteriorly, one pair posteriorly, and single seta at midlength), four setae between stripes (one pair anteriorly and one pair posteriorly); mesothorax, metathorax, and abdominal segments I–VIII with six setae at midlength (one pair lateral, one pair laterodorsal and one pair parasagittal); ventral surface of abdominal segments I–VIII with six setae at midlength (two pairs ventrolateral and one pair parasagittal). Legs separated by 8–12 times diameter of coxa.

*Etymology*. From Tupi-Guarani language; Tuja = adult, mitã = child; allusion to neoteny. Gender: neuter.

*Composition and distribution*. Only *T. plenalatum* (Brazil: Minas Gerais).

*Tujamita plenalatum* sp. nov.; ZooBank LSID: urn:lsid:zoobank.org:act:227EB9C4-6D91-49BD-9D78-6BA79777A5E1.

*Type material*. Holotype, male, “Brazil, Minas Gerais state, Itajubá municipality, Municipal Biological Reserve of Serra dos Toledos (22 °25′21.3″S 45 °22′06.2″W), 1,358 m, malaise trap, 15.X.–8.XI.2015, Rosa S.P. & Dias D. leg.” (deposited at Museu de Zoologia da Universidade de São Paulo). For information on paratypes and other material examined see the original Article.

*Diagnostic description*. Adult male. Labrum four times as wide as long, anterior margin rounded; maxillary palpus 5-segmented; pronotum with lateral carina; elytra not tapered apicad, apices contiguous; hind wing 0.2 times as long as elytra; abdomen relatively wide, with sides rounded and tapered apically; phallus and parameres together 1.1 times as wide as long; endophallus emerging from dorso-apical oval orifice on dorsal surface of phallus. For more details see the original Article.

*Etymology*. From Latin; plenus = full, alatus = winged; allusion to complete elytra.

*Jurasaidae fam. nov*.; ZooBank LSID: urn:lsid:zoobank.org:act:49654C87-BE63-4323-94F9-1BD97EF5E84B.

*Type genus*. *Jurasai* gen. nov.

*Diagnostic description*. **Male**. Body soft; head declivous, frontoclypeal suture absent, labrum sclerotized, free, separated from head capsule by membrane; antennal insertions elevated and visible from above; antenna filiform, with 11 antennomeres. Mandible falcate, unidentate; maxillary cardo, stipes, galea and lacinia indistinct, last palpomere shorter than all remaining palpomeres combined; gular sutures separated; two posterior tentorial pits present. Thorax with pronotum wider than long, narrower than elytral base; prosternum keel-shaped, strongly convex medially, prosternal process not extending beyond coxae; procoxal cavities open internally and externally, widely separated by prosternal process; mesoventrite evenly sclerotized with anterior margin arcuate, separated from metaventrite by feeble suture; leg with meso- and metacoxae oblique, pro- and mesotrochantin exposed; procoxa, mesocoxa and mesal part of metacoxa conical and strongly projecting, tibial spurs long, tarsi 5-5-5, without lamellae or pulvilli. Elytra soft, irregularly punctate, epipleura gradually narrowed posteriorly; wings folded longitudinally in resting position, longer than elytra, hiding the abdomen in dorsal view, veins reduced or blurred, cells and transverse cross veins absent, apical field with three triangular sclerotizations and a notch between two most apical ones. Abdomen with five free ventrites (i.e., sternites III–VII); sternites II and VIII largely membranous and concealed; sternite IX with apex bilobed, densely pilose and projecting above phallus and parameres; punctures surrounded by usually 2–3 campaniform sensilla in a dog’s-paw pattern. Aedeagus trilobate, not entirely retractable into abdomen, phallobase 2.0–2.7 times as long as wide, 1.4–2.6 times as long as parameres, sheathed by a tubular membrane which opens anteriorly into a pair of large balloon-like membranous vesicles; parameres and phallus together 1.1–1.6 times wider than long, basal struts of phallus absent; endophallus emerging from dorso-apical opening, flagellum absent. **Female**. Body elongate, wingless, with varying degree of neoteny, but always with at least meso-/metathorax and abdomen larva-like (except for ooporus in posterior margin of sternite VIII); leg short, with paired claws; ooporus with pair of membranous lobe-like valves, each with supporting sclerotized plate. **Larva**. Body cream or milky-white, cylindrical, with few setae; head sclerotized, wedge-like; epicranial sutures and endocarina absent; clypeolabrum triangular, translucent; gula, maxillae and labium fused to each other and to head capsule ventrally, anterior part of labium projected with pair of channels dorsally, fitting apical half of strongly sharpened mandibles, forming a beak-like mouthpart; basal half of mandibles elongate and retracted into anterior 2/3 of head, linked to inner sclerotized rod that extends into prothorax; pronotum with pair of parasagittal sclerotized stripes; prosternum with strongly sclerotized median longitudinal rod; leg short, 5-segmented; trochanter ring-shaped, pretarsus glabrous.

*Composition*. *Jurasai* (two species) and *Tujamita* (monotypic).

*Distribution*. Brazil (Minas Gerais, Rio de Janeiro).

