# Supplementary material for: Hidden diversity in the Brazilian Atlantic rainforest: the discovery of Jurasaidae, a new beetle family (Coleoptera, Elateroidea) with neotenic females
Source: Sci Rep. 2020 Jan 31;10:1544. doi: 10.1038/s41598-020-58416-6 (PMC6994542; doi:10.1038/s41598-020-58416-6)
Supplement: Supplementary file 1 — Supplementary Information. [file 41598_2020_58416_MOESM1_ESM.pdf]

Hidden diversity in the Brazilian Atlantic rainforest: the discovery of Jurasaidae, a new beetle family (Coleoptera, Elateroidea) with neotenic females

Simone Policena Rosa[1], Cleide Costa[2], Katja Kramp[3], and Robin Kundrata[4,*]

[1]Universidade Federal de Itajubá, Instituto de Recursos Naturais, Av. BPS, 1303, 37500-903 Minas Gerais, MG, Brazil
[2]Museu de Zoologia, Universidade de São Paulo, Avenida Nazaré, 481, 04263-000 São Paulo, SP, Brazil
[3]Senckenberg Deutsches Entomologisches Institut, Eberswalder Strasse 90, 15374 Müncheberg, Germany
[4]Department of Zoology, Faculty of Science, Palacky University, 17. listopadu 50, 771 46 Olomouc, Czech Republic
*E-mail: robin.kundrata@upol.cz

List of Supplementary Materials

Supplementary Text. The detailed family, generic and species descriptions; Biological notes.
Supplementary Table S1. Selected morphological characters of different sexes in Jurasaidae genera.
Supplementary Table S2. Morphological comparison of Jurasaidae with Elateroidea.
Supplementary Table S3. List of taxa used in the analyses, with voucher and GenBank accession numbers.
Supplementary Table S4. Results of the Xia's nucleotide substitution saturation test.
Supplementary Figure S1. Adult male habitus and morphology of *Jurasai* gen. nov.
Supplementary Figure S2. Adult morphology of *Jurasai itajubense* sp. nov.
Supplementary Figure S3. Adult female morphology of *Jurasai itajubense* sp. nov.
Supplementary Figure S4. Adult male morphology of *Jurasai digitusdei* sp. nov.
Supplementary Figure S5. Adult male habitus and morphology of *Tujamita plenalatum* sp. nov.
Supplementary Figure S6. Adult male morphology of Jurasaidae.
Supplementary Figure S7. Adult male morphology of *Tujamita plenalatum* sp. nov.
Supplementary Figure S8. Hind wings of Jurasaidae.
Supplementary Figure S9. Adult female morphology of *Tujamita plenalatum* sp. nov.
Supplementary Figure S10. Mature larval morphology of Jurasaidae.
Supplementary Figure S11. Mature larval head morphology of Jurasaidae.
Supplementary Figure S12. Immature morphology of Jurasaidae.
Supplementary Figure S13. Phylogenetic position of Jurasaidae within Elateriformia.
Supplementary Figure S14. DNA-based association of different sexes and semaphoronts in Jurasaidae.
Supplementary Video. Mating behavior of *Jurasai itajubense* gen. et sp. nov.

Supplementary Text

Jurasaidae fam. nov.
(Figs 2–5, S1–S12)

Type genus: *Jurasai* gen. nov.

Description. Adult male (Figs 4, 5, S1, S2a–h, S4–S8). Body with cuticle soft, softer on abdomen and elytra; vestiture of fine, semidecumbent and erect setae; punctures on ventral surface of thorax and abdomen surrounded by usually 2–3 campaniform sensilla (Fig. S1i). Head (Figs S2a, S4a, S5d, S6a–c,f,g, S7a,b,d) declined ventrally, mouthparts prognathous, posterior margin shortly covered by pronotum; eyes small (ocular index 0.2–0.3), widely separated, protruding laterad, coarsely facetted, weakly emarginate posteroventrally; frontoclypeal suture absent; labrum (Figs S2a, S7b,c) attached ventrally to frontoclypeus, evenly sclerotized, separated from head by membrane. Antennal insertions elevated, exposed from above, separated by 3.6–4.0 times antennal insertion width; antenna (Fig. S1d) with 11 antennomeres, filiform, reaching anterior third of elytra. Mandible (Figs S2a, S4a, S6a,c,f,g, S7a–d) narrow, falcate, unidentate; mola and prostheca absent. Maxilla (Figs S6d,g,i, S7d) with palpus about 2/3 as long as head ventrally, with 4 or 5 palpomeres, sparsely setose; apical palpomere fusiform with basiconic sensilla at apex; galea, lacinia, cardo, stipes, mentum and postmentum indistinct. Labium (Figs S6c,g,h, S7d) with ligula absent, prementum partly sclerotized, palpi 2-segmented, apical palpomere fusiform, 4–8 times longer than basal palpomere. Gular sutures separated, parallel-sided; two posterior tentorial pits present; cervical sclerites (Figs S4e, S7e) divided into two sections connected by membrane; corpotentorium absent.

Pronotum (Figs S2b, S4b, S6j,k) 1.5–1.7 times wider than long, 0.7 times narrower than elytral base; hypomeron with inner margin curved. Prosternum (Figs S4c–e,g,h, S6k, S7f) evenly sclerotized, anterior part in front of coxae transverse, about 10 times wider than long, lateral part 0.2–0.3 times as long as procoxal cavity, anterior corner fused to hypomeron in strongly sclerotized area; median part strongly convex, with keel-shaped elevation between procoxae; prosternal process wide, somewhat trapezoidal, not surpassing procoxal cavities; procoxal cavity slightly oblique, broadly open internally and externally, separated at middle by distance as long as procoxal cavity at widest place; trochantin exposed, membrane behind coxae with tubular spiracle. Scutellar shield (Figs S4i, S6j) gradually elevated above mesoscutum. Mesoventrite (Figs S4f–h, S7f–h) evenly sclerotized, 0.9–1.3 times as wide as long, with anterior margin arcuate, lateral margins divergent up to midlength and then tapered, apex rounded, fused to metaventrite; mesanepisternum (Figs S4g, S7h) wider than lateral half of mesoventrite; mesepimeron narrow with outer posterior angle acute and produced; mesocoxal cavity oblique, 1.7–2.0 times wider than long, open to both mesanepisternum and mesepimeron; separated at middle by 0.7–1.3 times mesocoxal cavity width; mesotrochantin exposed. Metanotum wider than long, lateral margins convergent posteriad, alacrista extending up to posterior margin, sinuous at anterior 1/2 then convergent posteriad; metascutellum sparsely setose, with intrascutal suture developed; median part of postnotal plate elongate, with longitudinal apodeme. Metaventrite (Figs S4f, S7g) 0.9–1.3 times as wide as long, 2.5–2.9 times longer than mesoventrite, anterior 1/3 with paired concavities to fit mesocoxae; discrimen 1/3 as long as metaventrite, posterior process between metacoxae split at middle;

metanepisternum tapered posteriorly. Metendosternite (Fig. S7i) with lamina triangular, lateral arms indistinct, anterior process elongate and linear, anterior tendons contiguous at base. Elytra (Figs S1a,f, S5a) shorter (covering up to half of abdominal sternite VII) to little longer than abdomen; irregularly punctate, striae absent; epipleura weakly co-adapted to pterothorax but not to abdomen. Hind wing (Fig. 8) folded longitudinally in rest, covering abdomen in dorsal view; RA extending on posterior 3/4, radial cell absent; apical field with three triangular sclerotizations and notch between two apical ones. Leg (Figs S1b,c,e, S5b,c,e) slender, long (distal half of profemur and almost entire meso- and metafemur visible in dorsal view); mesoleg 1.1 times longer than proleg, metaleg 1.1 times longer than mesoleg; femur sparsely and finely setose, tibia densely covered with shorter dark thick setae, tarsomeres with long setae, denser on ventral surface. Pro- and mesocoxae conical and projecting; metacoxae almost contiguous, gradually shortened on outer 1/2 in ventral view, extending to lateral part of metaventrite, without plates, mesal portion conical and projecting; angle formed at midline by horizontal line and line tangential to anterior edge of metacoxa 45–60 degrees (Figs S4f, S7g); trochanters obliquely attached to femur; protrochanter 0.2–0.3 times as long as profemur; mesotrochanter 0.2–0.3 times as long as mesofemur; metatrochanter 0.4 times as long as metafemur; femora weakly swollen, tibiae gradually widened apicad, each with pair of apical spurs; tarsi 5-5-5; tarsomeres I–IV gradually shorter, tarsomere V 1.1 times as long as combined lengths of tarsomeres III and IV; tarsomeres I–III slender, obliquely truncate apically; tarsomere IV truncate or deeply notched; claws (Fig. S6l) simple.

Abdomen (Figs S1h, S4h, S5f) with tergites and sternites soft, weakly sclerotized; sternite II largely membranous and concealed under metacoxae, except small lateral part which is sclerotized and visible in lateral view; five ventrites visible in dorsal view (i.e., sternites III–VII), free, with short membranes between them. Sternites II–VII transverse, almost equal in length, lateral edges bent dorsad covering lateral edges of tergites, pleurae hidden in ventral groove, spiracles on anterior edge of pleurae; surface punctate-setose, punctures surrounded by usually 2–3 campaniform sensilla, most of them in a dog's-paw pattern (Fig. S1i). Tergite VIII (Figs S2c, S4j, S7j) sclerotized and exposed, lateral edges bent ventrad; sternite VIII (Figs S2d, S4k, S7k) reduced or membranous, glabrous, largely or completely hidden under sternite VII. Tergite and sternite IX fused along lateral arms (Figs S4l, S7l,m); apical parts of tergites IX and X membranous, apparently fused. Sternite IX (Figs S2e, S4l, S5g, S7m) with anterior and lateral margins more sclerotized, contiguous to lateral sclerotized arms; apex bilobed, densely setose and projecting above phallus and parameres. Luminous organs and glandular pores absent. Aedeagus (Figs S2f–h, S4m–o, S5g, S7o–q) always exposed, not entirely retractable into abdomen; phallobase elongate, 2.0–2.7 times longer than wide, 1.4–2.6 times longer than parameres, weakly concave ventrally, lateral margins curved inwards, with anterior corners obliquely truncate, anterior margin widely emarginate, posterior margin rounded or truncate; phallobase sheathed by tubular membrane which opens on each anterolateral angle into balloon-like membranous vesicle which is as large as aedeagus. Phallus and parameres together 1.1–1.6 times wider than long. Parameres slightly shorter than phallus, conical, connected to phallus by translucent membrane, without sclerotized articulation, dorso-apical surface with semicircular asperities (Fig. S4p). Phallus without basal struts, tapered apicad, apex projected ventrally; endophallus emerging from apical opening on dorsal surface of phallus; reproductive tract (Fig. S7o) with two large, reniform testes, linked to each other by vas deferens, with only one testis opening in ejaculatory duct.

Adult female (Figs 2e,f, 4, S2i,j, S3, S9). Body length 4–6 mm. Larviform, wingless; head (Figs S3a–c, S9a–c) with adult or larval features different from those of adult male and mature larva (Table S1); abdomen similar to abdomen of larva, except for ooporus at posterior margin of sternite VIII (Fig. S3j,k); reproductive tract opening in ooporus in two membranous lobe-like valves, with weakly sclerotized supporting plates on each valve; vagina short to elongate, narrow; bursa copulatrix spherical, attached at base to spermatheca and pair of small colleterial glands (Figs S2i,j, S9h).

Mature larva (Figs 1d, 2a–c, S10, S11, S12a–f, i–l). Body length 6–7 mm. Body parallel-sided, cylindrical (depressed with marked pleurae when walking), thoracic and abdominal segments with anterolateral plicae; tegument soft, white, except for head, legs, median ventral rod and a pair of dorsal stripes on prothorax, which are sclerotized and brown. Soft tegument microsculptured in meshwork polygonal pattern (Fig. S10m). Vestiture consisting of few fine setae. Head (Figs S10a–e,j–l, S11a,c, S12c,d,i,j) wedge-like, prognathous, shortly retractable, 0.4–0.5 times narrower than prothorax; region at base of antenna translucent; epicranial sutures absent; labrum fused to head, forming triangular and translucent clypeolabrum produced anteriad; each dorsal and ventral anterior corners at base of antenna with 2 long setae; gula and ventral base of mouthparts fused to each other and to head capsule forming smooth single plate. Stemmata absent. Antenna (Fig. S10c) 2-segmented, basal antennomere 1.1–2.0 times longer than apical one, glabrous, with large dome-like sensorium ventrally; apical antennomere with long basiconic sensilla. Mandible (Figs S11b,d,e, S12e,f,k,l) symmetrical, with internal channel extending from basal 1/3 to apex; apical half strongly sharpened and fitting into labial channels; basal half laminar, elongate, retracted into the anterior 2/3 of head, condyle strongly sclerotized, projected ventrad, fused to head capsule; acetabulum indistinct; mola and prostheca absent. Hypopharyngeal bracon hyaline or sclerotized extending across the most posterior edges of mandibles; elongate sclerotized rod extending from posterior tip of each mandibular channel and surpassing posterior edge of head, entering prothorax (Fig. S11). Maxilla (Figs S10b,f, S12d,j) with cardo, stipes, galea and lacinia indistinct, maxillary palpus 3-segmented, basal palpomere with single seta ventrally, median palpomere with single seta dorsally, apical palpomere with basiconic sensilla apically. Labium (Figs S10b,d,e,l, S12c,d,i,j) with mentum, prementum and palpi fused and produced anteriad forming beak-like mouthpiece as long as or longer than maxillary palpi, dorsally with a pair of channels which enclose mandible apices. Prothorax (Fig. S12a,b) about twice wider than long, 0.5–0.8 times as long as mesothorax, sides rounded, convergent anteriad; pronotum with a pair of parasagittal sclerotized stripes; prosternum with median longitudinal, strongly sclerotized endocarinate rod. Metathorax slightly wider and longer than mesothorax. Leg (Fig. S10g) lightly pigmented, subcylindrical, narrowing apicad, with a few setae, short, widely separated by distance about 5–12 times diameter of coxae; coxae about 3 times wider than long, trochanters oblique, very short, ring-shaped, femora about 1.5 times wider than long, tibiotarsus twice longer than wide, 1.5 times longer than femora; pretarsus slender, glabrous, 0.7–1.0 times as long as tibiotarsus. Abdomen (Figs S10h,i, S12a,b) parallel sided, segments I–VIII 1.2–1.5 times longer than mesothorax, segment IX with apex rounded, sparsely setose; setae denser apically; urogomphi absent; segment X dorsoapical, conical, weakly protruded. Thoracic and abdominal spiracles (Fig. S10h) dorsolateral with closing apparatus.

Male pupa (Figs 3, S12g,h). Body length 2.5–3.0 mm. Adecticous, exarate, tegument smooth, white, sparsely setose. Head perpendicular to pronotum, shortly inserted into prothorax; eyes visible dorsally; labrum as long as mandibles. Pronotum with a few setae on anterior and lateroposterior margins. Abdominal segments I–VIII

transverse; tergite I with setae laterally; segments II–VIII with setae on posterior margins, sparser ventrally; tergite IX subtriangular. Urogomphi and gin-traps absent.

Female pupa (Fig. 2d). Body length 4–6 mm. Like adult female, except for entire tegument of head and legs smooth and white; leg and mouth joints swollen, inarticulate; claws indistinct. Capable to move, walk and bury into soil.

Comparison with similar/related taxa. Jurasaidae males superficially resemble other small soft-bodied elateroids, with which they share many morphological characters (Table S2). They differ from all other Elateroidea in the following combination of characters: the ventral surface of thorax and abdomen with punctures surrounded by 1–3 campaniform sensilla (but usually 2–3, especially on abdomen), most of them in a dog's-paw pattern (Figs S1i, S2c, S7j), abdomen with five free ventrites connected by short membranes (Figs S1h, S5f), apex of sternum IX with a pair of elongate lobes projecting above the parameres and phallus (Fig. S7n), very short phallus and parameres which are not retracted into the abdomen, and the phallobase sheathed by a tubular membrane which opens anteriorly into a pair of large, balloon-like, membranous vesicles (Figs S4m–o, S5g, S7o–q). Apart from the above-mentioned unique morphology, Jurasaidae differ from each of the other soft-bodied elateroid groups in the Neotropics, i.e., Phengodidae, Omethidae: Telegeusinae, Cantharidae, Lampyridae, and Lycidae by a number of additional features. They can be distinguished from the currently defined Neotropical Phengodidae (i.e., Mastinocerinae and Phengodinae, *sensu* Zaragoza-Caballero & Zurita-García 2015) by the filiform antennae, evenly sclerotized labrum separated from head by a membrane, indistinct cardo, stipes, galea, lacinia, mentum and submentum, and phallus without flagellum (Mastinocerinae and Phengodinae have pectinate or flabellate antennae, labrum membranous and hidden or connate to clypeus; galea, lacinia, stipes, cardo and prementum usually visible, and the phallus with flagellum) (Witmmer 1976, Costa & Zaragoza-Caballero 2010, Zaragoza-Caballero & Pérez Hernández 2014). Jurasaidae males share with Omethidae the filiform antennae and the labrum which is distinctly separated from head. Particularly, *Tujamita* resembles the representatives of the subfamily Omethinae in having elytra parallel-sided and conjointly rounded apically, whilst *Jurasai* resembles more Telegeusinae in the abbreviated elytra with separated apices, and the reduced hind wing venation. However, Jurasaidae can be easily distinguished from most Omethidae by the combination of declined head, labrum with anterior margin rounded or shortly notched medially, apical maxillary palpus shorter than the remaining ones combined, and the tibial spurs not serrated (head prognathous, and the anterior edge of labrum deeply marginate or dentate in Omethinae and Telegeusinae; apical maxillary palpus long and tibial spurs serrate in Telegeusinae). *Jurasai* species share with *Tarsakanthos* Zagagoza-Caballero, 2008 (Telegeusinae: Penicillophorini,) the elevated antennal insertions and the extreme reduction of the hind wing venation, but it can be distinguished by the two posterior tentorial pits, evenly sclerotized labrum, filiform antenna, and the maxillary palpus 4- or 5-segmented, while *Tarsakanthos* has a single tentorial pit, labrum anteriorly membranous, serrate antenna, and the maxillary palpus 3-segmented (Zaragoza-Caballero 2008, Ramsdale 2010a, Zaragoza-Caballero & Zurita-García 2015). Jurasaidae can be promptly separated from Cantharidae, Lampyridae and Lycidae by their well-sclerotized labrum, conical coxae, tarsomeres not bilobed and without pads, elytra smooth, and the abdomen with five ventrites (labrum usually indistinct, tarsomeres usually widened and bilobed, and abdomen with 7–8 ventrites in Cantharidae and Lampyridae; coxae globular, tarsomeres 2–4 with membranous pads,

elytra often with costae, and the abdomen with 7–8 ventrites in Lycidae) (Bocak & Bocakova 2010, Branham 2010, Ramsdale 2010b).

Females of Jurasaidae, similar to those of Phengodidae, retain many larval features and therefore present a high level of neoteny. Interestingly, the females of both *Jurasai* and *Tujamita* exhibit different degrees of neoteny (Table S1). The female of *J. itajubense*, unlike phengodids, has the head different from that of the mature larva, with a combination of larval and adult features different from those of both male and mature larva. Larviform features include the leg with tibia and tarsus separated and claws paired, antennae with 4 antennomeres, and a pair of large stemmata. It has free labrum, falcate mandibles, maxilla and labium separated with distinct palpi like in adults but with different shapes, ventrally the head lacks gular sutures and the posterior tentorial pits. The chaetotaxy of thorax and abdomen is the same as in larva. The female of *Tujamita plenalatum* has the adult-like head, prothorax and legs, but also different from those of the male in the following characters: eyes smaller and flat, posterior tentorial pits absent, antenna moniliform and with 9 antennomeres, pronotum without lateral carina, prosternum reduced to a narrow, transverse, sclerotized strip, and legs short with tarsus 4-4-4. The mesothorax, metathorax and abdomen are evenly setose, with very short setae. Jurasaidae females share a unique morphology of the ooporus. It is located on the posterior margin of the sternite VIII, with a pair of membranous lobe-like valves, each one with a supporting sclerotized plate (Fig. S3j,k).

Larvae of Jurasaidae are more similar to the larvae of its sister-group Cerophytidae (rare click beetles) than to the larvae of the soft-bodied elateroids. They share the cylindrical, white and grub-like body, very small wedge-shaped head without dorsal and ventral epicranial sutures, non-opposable, flattened and channeled mandibles, labium with channels to fit the mandibular apices, prosternum with a median sclerotized endocarinate rod, and short legs. The shape of head, reduced epicranial sutures and the non-opposable mandibles fused to head capsule are also shared with larvae of Throscidae and most Eucnemidae. Additionally, these groups share with Jurasaidae the ring-shaped trochanter. The sucking apparatus of Jurasaidae larvae is unique in Coleoptera in consisting of a beak-like mouthpart formed by gula, maxillae and labium fused to each other and to the head capsule ventrally, the labium with dorsal channels approximate at midline, forming a narrow groove between the channels and mandibular apex, and strongly sharpened mandibles which bases are long and extend into the anterior third of head (Figs S11, S12e,f,k,l).

Genera and species included in Jurasaidae

Jurasai gen. nov.

Jurasai itajubense sp. nov. Distribution: Brazil: Itajubá, Minas Gerais state.

Jurasai digitusdei sp. nov. Distribution: Brazil: Terezópolis, Rio de Janeiro state.

Tujamita gen. nov.

Tujamita plenalatum sp. nov. Distribution: Brazil: Itajubá, Minas Gerais state.

***Jurasai* gen. nov.**

(Figs 1d, 2a,c–e, 3a,b, 4, 5a–d, S1–S4, S6a–i, S8a,b, S10a–i, S11a,b, S12a–h)

Type species. *Jurasai itajubense* sp. nov.; by present designation.

Etymology. From Tupi-Guarani language; Jura = mouth; saí = minuscule, thin; allusion to the larval mouth. Gender: neuter.

Diagnosis. Adult male (Figs 4, 5a–d, S1, S2a–h, S4, S6a–i, S8a,b). Body 4–5 times longer than wide (in dorsal view, up to wing apex); pronotum (Figs S2b, S4b,d, S6e) distinctly widest at anterior half, posterior half narrowed posteriad; without lateral carinae; posterior edge smooth, not marginated; mesoventrite (Figs S4f–h, S6e) 1.1 times longer than wide, with anterior margin deeply arcuate; mesepimeron with anterior part indistinct in lateral view, separated from mesanepisternum by weakly impressed suture; mesocoxal cavities separated at middle by 1.3 times mesocoxal cavity width; metaventrite (Fig. S4f) 1.3 times longer than wide, 2.5 times longer than mesoventrite, widest at anterior 1/3. Elytra (Figs 5a,c, S1a,f) shorter than abdomen, tapered, with median edges separated and divergent apicad, lateral edges sinuate, apices swollen; hind wing (Fig. S8a,b) surpassing elytral apex by 0.5–0.7 times elytral length, apical field long (1/2 of total wing length). Tarsomere IV (Fig. S1e) evenly sclerotized, truncate apically. Abdomen (Fig. S1h) narrow, with sides subparallel; phallus and parameres (Figs S2f,g, S4m–o) together 1.6 times wider than long; endophallus emerging from dorso-apical elongate notch.

Adult female (Figs 2e, 4, S2i,j, S3) (based on *J. itajubense* only)**.** Abdomen and thorax almost equal to those of larvae; head with pigmented stemmata and lens; mandibles falcate; labrum free; maxilla and labium separated, labium not channeled to fit mandibles; antenna with 4 antennomeres; leg with tibia and tarsus separated, tarsus 1-1-1, with paired claws.

Larva (Figs 1d, 2a,c, S10a–i, S11a,b, S12a–h) (based on *J. itajubense* only). Slender, 9–10 times longer than narrow; clypeolabrum (Fig. S12c) with two long setae. Mandibles and labium (Fig. S10b) forming elongate and sharpened beak-like mouthpart; mandibles (Figs S11b, S12e,f) with apices gradually sharpened apicad, parallel-sided, base elliptical; hypopharyngeal bracon hyaline.

***Jurasai itajubense* sp. nov.**
(Figs 1d, 2a,c–e, 3a,b, 4, 5a,b, S1a–e, S2, S3, S6a–e, S8a, S10a–i, S11a,b, S12a–h)

Type material. **Holotype**: male, BRAZIL, Minas Gerais state, Itajubá municipality, Biological Reserve of Serra dos Toledos (22°25'21.3"S 45°22'06.2"W), 1,358 m, soil ravine, collected as pupa on 7.VI.2018 (adult on 5.VII.2018, died on 8.VIII.2018), Rosa S.P, Barbosa T. & Paiva J. leg. (MZUSP). **Paratypes** (5 males, 7 females): same locality as for the holotype, 1 male, 1 female, 6.IX.2017, Rosa S.P. & Ladenthin M. leg. (MZUSP); 1 male, collected as larva on 7.VI.2018 (adult on 3.VII.2018, died on 25.VIII.2018), Rosa S.P, Barbosa T. & Paiva J. leg. (MZUSP); 1 female, 1.III.2018, Rosa S.P., Barbosa T. & Paiva J. leg. (MZUSP); 1 male, dissected, collected as larva on 24.V.2016 (adult on 5.VII.2016), Rosa S.P. & Lopes A. leg. (UNIFEI); 1 male, head and thorax metallized for SEM, collected as pupa 28.VIII.2017, Rosa S.P. & Ladenthin M. leg. (UNIFEI); 1 male (RK1170, DNA extracted), 07.VI.2018, Rosa S.P., Barbosa T. & Paiva J. leg. (UPOL); 1 female, dissected, 2.IX.2016, Rosa S.P. & Lopes A. leg. (UNIFEI); 1 female, metallized for SEM, 29.IX.2016, Rosa S.P. & Lopes A. leg. (UNIFEI); 1 female, metallized for SEM, 20.X.2017, Rosa S.P. & Ladenthin M. leg. (UNIFEI); 2 females (RK1166, RK1168, DNA extracted), 07.VI.2018, Rosa S.P., Barbosa T. & Paiva J. leg. (UPOL).

Other material examined. Same locality as for the holotype, 1 larva, 16.VI.2016, Rosa S.P. & Lopes A. leg. (MZUSP); 1 larva, metallized for SEM, 29.IX.2016, Rosa S.P. & Lopes A. leg. (UNIFEI); 1 larva, 20.IV.2017, Rosa S.P. & Ladenthin M. leg. (UNIFEI); 1 larva, 28.VIII.2017, Rosa S.P. & Ladenthin M. leg. (UNIFEI); 1 larva, 23.III.2018,

Rosa S.P., Barbosa T. & Paiva J. leg. (UNIFEI); 1 larva, 27.III.2018, Rosa S.P., Barbosa T. & Paiva J. leg. (UNIFEI); 12 larvae, 12.IV.2018, Rosa S.P., Barbosa T. & Paiva J. leg. (MZUSP); 2 larvae (RK1172, RK1173, DNA extracted), 12.IV.2018, Rosa S.P., Barbosa T. & Paiva J. leg. (UPOL); 8 larvae, 7.VI.2018, Rosa S.P. Barbosa T. & Paiva J. leg. (MZUSP); 1 larva (RK1175, DNA extracted), 07.VI.2018, Rosa S.P., Barbosa T. & Paiva J. leg. (UPOL); 4 larvae, 7.VI.2018, Rosa S.P., Barbosa T. & Paiva J. leg. (UNIFEI); 1 larva, 23.VIII.2018, Rosa S.P., Ribeiro R. & Paiva J. leg. (UNIFEI); 1 male pupa, 16.VI.2016, Rosa S.P. & Lopes A. leg. (UNIFEI); 2 male pupae, 07.VI.2018, Rosa S.P., Barbosa T. & Paiva J. leg. (MZUSP).

Etymology. From the type locality, Itajubá, in Minas Gerais state, Brazil.

Diagnosis. Males of *J. itajubense* differ from those of *J. digitusdei* (in parentheses) in the anterior margin of labrum emarginate (rounded); maxillary palpi 4-segmented (5-segmented), sternite VIII partly exposed (concealed), elytra more tapered, wings with only RA1+2 and MP1+2 veins (R3, RA1+2, RA3+4, RM loop and medial field veins present), and apices of parameres tapered and curved inwards (sausage-like, directed posteroventrad).

Description. **Adult male** (Figs 4, 5a,b, S1a–e, S2a–h, S6a–e, S8a). Total length from head to apex of abdomen 2.5 mm, up to apex of wings 3.5 mm. Body (Figs 5a,b, S1a–c) in dorsal view (up to wing apex) about 4.5–5.0 times longer than wide. Coloration of head, pronotum and elytra light brown, apex of elytra darker; antennae, ventral pterothorax and legs pale yellow, except brown tibiae; abdominal ventrites light brown. Head (Figs S2a, S6a,b) in dorsal view as wide as long, densely punctate, punctures large, umbilicate; posterior part to eyes 0.6–0.7 times shorter than anterior part, narrowing posteriad; posterior margin straight; labrum 1.8–2.0 times wider than long, anterior margin notched at middle; antenna (Fig. S1d) as long as distance from head to elytral apex; Mouthparts (Fig. S6a–d) with maxillary palpus 4-segmented, palpomere I and III subequal in length, each longer than palpomere II, apical palpomere as long as combined lengths of palpomeres II and III, palpiger membranous; prementum with lateral borders sclerotized, with 2 setae, labial apical palpomere about twice longer than basal palpomere.

Pronotum (Figs S2b, S6e) 1.5–1.6 times wider than long, lateral margins rounded; anterior margin slightly curved to straight, posterior margin straight; anterior and posterior angles obtuse; surface evenly densely setose-punctate, punctures umbilicate; prosternal process with apex truncate. Scutellar shield 1.5–2.0 times wider than long, narrowing posteriad, apex rounded or truncate; finely setose-punctate; meso-metaventrite suture indistinct in ventral view and weakly impressed in lateral view. Elytra (Figs 5a, S1a) tapered, with median edges divergent on posterior 2/5–1/2, surface densely and finely punctate. Hind wing (Fig. S8a) surpassing elytral apex by 0.5–0.6 times elytral length; r3 and RA3+4 indistinct, corresponding area with elongate sclerotization; medial area with only MP1+2 and medial spur, both weakly impressed; anal field short, without veins, anal notch small; apical field with three triangular light sclerotizations occupying half of total wing length; apex notched between two apical-most sclerotizations.

Abdomen (Fig. S1h) with tergite VIII (Fig. S2c) rectangular, 1.8 times wider than long, evenly weakly sclerotized, with a few setae on posterior 2/3; sternite VIII (Fig. S2d) semicircular, about twice wider than long, as long as 1/3 of sternite VII, evenly membranous, glabrous, anterior part hidden under sternite VII; posterior part of

tergites IX and X membranous, without microsetae. Sternite IX (Fig. S2e) with sclerotized lateral arms and anterior margin lyre-shaped, median part parallel-sided on anterior 2/3, then abruptly tapered to apex; apical lobes about 1/3 as long as whole sternite IX, with long setae. Aedeagus (Fig. S2f–h) with phallobase about twice longer than wide, 1.4 times longer than parameres; parameres strongly convex laterally (semicircular), entirely hiding phallus in lateral view, semispherical in dorsal and ventral view, apices curved inwards; phallus with sides convergent to apex, apex slightly widened.

Adult female (Figs 2e, 4, S2i,j, S3). Body length 5–8 mm. Head sclerotized, light brown, stemmata black; thorax and abdomen larviform, milky-white, with chaetotaxy equal to those of larvae; legs lightly pigmented; two small light brown plates on ooporus. Head (Fig. S3a–c) weakly convex, about twice wider than long, 0.5 times as wide as prothorax; lateral margins rounded; clypeus fused to frons, frontoclypeus gradually declivous, anterior margin emarginate from one mandible base to another; dorsal surface smooth with one long seta at base of antenna and a few microsetae lateroposteriorly; one large stemma on each side, with well-developed lens; labrum free, twice wider than long, 0.2 times as wide as anterior margin of frontoclypeus in dorsal view, with a few short to long setae. Antennae (Fig. S3d,e) separated by 2.5 times basal antennomere diameter, with 4 antennomeres; antennomeres increasing in length and decreasing in width apicad, sparsely setose; subapical antennomere with 5 long conical sensoria lateroventrally, apical antennomere inserted dorsally into subapical one, with several short and long basiconic sensilla. Mandibles unidentate, non-overlapping, lacking prostheca, penicillus and mola. Maxillary cardo, stipes, galea and lacinia indistinct, maxillary palpus 4-segmented, palpomeres increasing in length and decreasing in width apicad; apical palpomere 1.3 times longer than combined lengths of palpomeres II and III, with long basiconic sensilla at apex. Prementum with a pair of microsetae at base, a few setae apically, ligula absent, palpus 2-segmented, suture between palpomeres feeble, apical palpomere elongate, 3 times longer than basal one, reaching apex of mandibles, with two basiconic sensilla at apex; mentum and postmentum indistinct. Ventral surface smooth with 3 setae at base of antennae and a few microsetae basally; gula, posterior tentorial pits and cervical sclerites absent. Thorax equal in shape to that of larva. Mesothoracic dorsoventral spiracle lacking closing apparatus. Leg (Fig. S3f,g) short, cylindrical, with a few setae, coxa oblique, 3 times wider than long; trochanter very short, ring-like; femur 1.2 times wider than long; tibia and tarsus separated; tibia as long as wide; tarsus twice longer than wide, 1.7 times longer than tarsus; tarsus 1-1-1; claws paired, simple, glabrous. Reproductive tract occupying segments VII and VIII, vagina elongate, spermatheca sclerotized, colleterial glands with ducts moderately elongate (Figs S2i,j).

Mature larva (Figs 1d, 2a,c, S10a–i, S11a,b, S12a–h). Length: 4–7mm. Body (Figs 2a,c, S12a,b) slender, 9–10 times longer than wide, milky-white. Head (Figs S10a, S11a, S12c,d) flattened dorsally; clypeolabrum flat with 2 parasagittal long setae; ventral and posterior posterior margins straight to slightly rounded; basal antennomere 1.1–1.4 times longer than apical one. Mandibles (Figs S11a,b, S12e,f) parallel to each other, apical half gradually sharpened, basal half flattened, laminar, semielliptical, with membranous translucent hypopharyngeal bracon; posterior sclerotized rods straight; labium (Figs S10e,f, S11a, S12c,d) longer than maxillary palpus, apex with basiconic sensilla, ventrally with 2 short setae and feeble, incomplete median suture. Prothorax (Fig. S12a) about twice wider than long, 0.6–0.8 times as long as mesothorax; dorsal surface with four setae between lateral edge and lateral sclerotized stripe (one pair anteriorly and one pair posteriorly), 2 setae between stripes anteriorly. Metathorax,

mesothorax and abdominal segments I–VIII (Fig. S12a,b) with one pair of laterodorsal setae near anterior margin, one pair of laterodorsal setae near posterior margin, and a pair of lateroventral setae near posterior margin. Leg (Fig. S10g) separated by 5–6 times diameter of coxa.

Male pupa (Figs 3a,b, S12g,h). Body length 2.5 mm. Frons (Fig. S12h) with a pair of setae between antennal insertions; antennal insertions separated by about two scape widths; labrum with apex truncate and with 4 setae. Pronotum with stiff tuberculate setae; legs with tuft of setae on femur-tibia joint. Abdomen 2.3 times longer than combined lengths of head and prothorax in dorsal view; tergite IX with tuberculate stiff setae lateroposteriorly, denser at apex.

Female pupa (Fig. 2d). Body length 5–7 mm. Like adult female, but tegument entirely smooth and white, leg and mouth joints swollen, inarticulate; leg truncate at apex.

***Jurasai digitusdei* sp. nov.**
(Figs 5c,d, S1f–i, S4, S6f–i, S8b)

Type material. **Holotype**: male, BRAZIL, Rio de Janeiro State: Teresópolis, Parque Nacional da Serra dos Órgãos, malaise trap, PVE 6B (22º28'11"S 43º 0'5.3"W, 868 m), VI.2015, Silveira & Khattar leg. (DZRJ). **Paratypes** (8 males): same locality as for the holotype, 1 male, antennae dissected, PVE 6A (22º28'11.5"S 43º0'6.0"W, 877 m), VI.2015 (DZRJ); 2 males, both with abdomen and aedeagus dissected, PVE 10A (22º26'54.2"S 43º00'49"W, 1482 m), VI.2015, Silveira & Khattar leg. (DZRJ); 2 males, PVE 10B (22º26'51.0"S 43º00'46.9"W, 1444 m), VI.2015, Silveira & Khattar leg. (DZRJ); 1 male, PVE 11A (22º2'7.9"S 43º00'53.8"W, 1681 m), VI.2015, Silveira & Khattar leg. (DZRJ); 1 male, dissected (head metallized for SEM), PVE 12B (22º27'17.4"S 43º00'57.3"W, 1681 m), VI.2015, Silveira & Khattar leg. (UNIFEI); 1 male (RK1171, DNA extracted), 1.–15.VI.2017, Silveira L., Gomes C., Quintarelli F. & Esperança A. leg. (UPOL).

Etymology. From Latin; digitus = finger, dei = of god; allusion to "Dedo de Deus", a mountain peak near the type locality in Serra dos Órgãos National Park, whose shape resembles a hand pointing up towards the sky.

Diagnosis. Male of *J. digitusdei* differs from its congener *J. itajubense* by the following combination of characters: anterior margin of labrum rounded, not emarginate, maxillary palpus 5-segmented, elytra weakly tapered; R3, RA1+2, RA3+4, RM loop and medial field veins present, abdominal sternite VIII concealed, and parameres with apices sausage-like, directed posteroventrally.

Description. **Adult male**. Body length from head to apex of abdomen 2.5–3.0 mm, up to apex of wings 4.0–5.0 mm. Body (Figs 5c, S1f) in dorsal view (up to wing apices) about 4–5 times longer than wide. Head light to dark brown; mandibles yellow, head evenly colored or with lighter areas on posterior corners and between antennal insertions; antennomeres I–VII brown, antennomere VIII light brown, antennomeres IX–XI yellow; pronotum and elytra light to dark brown, apices of elytra darker; ventral surface of pterothorax and abdomen yellow to light brown, posterior margins of abdominal sternites lighter; legs amber except tibiae which are brown. Head (Fig. S4a) about as long as wide, densely punctate, punctures large, umbilicate; posterior part to eyes as long as anterior part, narrowing posteriad; posterior margin emarginate; labrum

4 times wider than long, anterior margin rounded; antenna as long as distance from head to elytral apices in dorsal view. Mouthparts (Figs S6f–i) with maxillary palpus 5-segmented, palpomeres I–III subequal in length, palpomere IV shortest, apical palpomere as long as combined lengths of palpomeres III and IV, with set of parallel slit-like sensilla laterally; palpiger with large triangular sclerotization; prementum with transverse sclerotization at base, densely setose, apical palpomere 4–5 times longer than basal one.

Pronotum (Fig. S4b) 1.6 times wider than long, lateral margins sinuous; anterior margin straight, posterior margin weakly produced posteriorly; anterior and posterior angles rounded; surface evenly densely setose-punctate, punctures umbilicate. Prosternal process (Fig. S4c) with apex rounded or slightly pointed. Scutellar shield (Fig. S4i) as long as wide, parallel-sided, posterior margin straight, finely setose-punctate; meso-metaventrite suture (Fig. S4f–h) distinct in both ventral and lateral view. Elytra (Figs 5c, S1f) tapered with median edges divergent on posterior 1/3–1/4; surface densely finely punctate. Hind wing (Fig. S8b) surpassing elytral apex by 0.6–0.7 times elytral length, RA1+2 and RA3+4 not contiguous, r3 elongate, parallel to narrow sclerotization, MP1+2, RM loop and medial spur present; MP3+4, Cu, AA3+4 and AP3+4, weakly impressed, branches indistinct; veins blurred, except SCA, RA and MP1+2; anal field inconspicuous, anal notch absent; apical field with three triangular sclerotizations occupying half of total wing length; apex notched between two apical-most sclerotizations.

Abdomen (Fig. S1h) with tergite VIII (Fig. S4j) 1.9 times wider than long, with sides weakly convergent anteriorly, posterior margin rounded, posterior half setose, with transverse membranous area on anterior margin; sternite VIII (Fig. S4k) reduced to glabrous, transverse and curved sclerotized strip, 7.8 times wider than long, hidden under sternite VII; posterior parts of tergites IX and X (Fig. S4l) with sclerotized glabrous strip contiguous to lateral arms of sternite IX; sternite IX (Fig. S4l) with lateral arms subparallel, anterior margin rounded; median part with sides rounded; apical lobes 1/4 of sternite IX total length, with short setae. Aedeagus (Fig. S4m–p) with phallobase 2.7 times longer than wide, 2.6 times longer than parameres; parameres strongly convex laterally (semicircular), apex strongly tapered, forming sausage-like lobe projecting posteroventrad, with circular microsensilla; phallus with lateral margins straight and gradually convergent apicad.

Female and immature stages. Unknown.

***Tujamita* gen. nov.**
(Figs 2a,b,f, 3c, 5e,f, S5, S6j–l, S7, S8c, S9, S10j–m, S11c–e, S12i–l)

Type species. *Tujamita plenalatum* sp. nov.; by present designation.

Etymology. From Tupi-Guarani language; Tuja = adult, mitã = child; allusion to neoteny. Gender: neuter.

Diagnosis. **Adult male**. Body (Figs 5e, S5a) in dorsal view (up to visible elytral apex) about 3 times longer than wide; pronotum (Figs S5d, S6j) weakly narrowed posteriad, with lateral margins subparallel and carinate; posterior edge marginated; mesoventrite (Figs S6k, S7f–h) 1.3 times wider than long, with anterior margin weakly arcuate; mesepimeron with anterior part distinct in lateral view, separated from mesanepisternum by grooved suture; mesocoxal cavities separated at middle by 0.7 times mesocoxal cavity width; metaventrite (Fig. S7g) 1.1 times wider than long, 2.8–

2.9 times longer than mesoventrite, widest at midlength. Elytra (Figs 5e, S5a) slightly shorter or as long as abdomen, parallel-sided, median edges contiguous to apex; apices flat. Hind wing (Fig. S8c) surpassing elytral apex by 0.2 times elytral length, apical field short (1/3 of total wing length). Tarsomere IV (Fig. S5e) deeply notched. Abdomen (Fig. S5f) wide, with sides rounded and tapered apicad; phallus and parameres (Fig. S7o,p) together 1.1 times wider than long; endophallus emerging from apical oval orifice on dorsal surface of phallus.

Adult female. Meso-, metathorax and abdomen almost equal to those of larva; head, pronotum and leg (Fig. S9a–g) adult-like but different from those of male: compound eyes very small, flat, not protruded; posterior tentorial pits absent; antenna moniliform, with 9 antennomeres; pronotal lateral carinae absent, prosternum reduced to very narrow sclerotized strip; tarsi 4-4-4.

Mature larva. Body stout, 5–6 times longer than wide; clypeolabrum (Figs S10j, S12i) with four long setae; mandibles and labium (Figs S10j,l, S11c–e) forming short, stout, beak-like mouthpart; mandibles (Figs S11d, S12l) with apices abruptly sharpened apically, convergent anteriad, base triangular; hypopharyngeal bracon sclerotized.

***Tujamita plenalatum* sp. nov.**
(Figs 2a,b,f, 3c, 5e,f, S5, S6j–l, S7, S8c, S9, S10j–m, S11c–e, S12i–l)

Type material. **Holotype**: male, BRAZIL, Minas Gerais state, Itajubá municipality, Municipal Biological Reserve of Serra dos Toledos (22°25'21.3"S 45°22'06.2"W), 1,358 m, malaise trap, 15.X.–8.XI.2015, Rosa S.P. & Dias D. leg. (MZUSP). **Paratypes** (5 males, 1 female): same locality as for the holotype, 1 male, 15.X.–8.XI.2015, Rosa S.P. & Dias D. leg.; 1 male, 24.IX.–15.X.2015, Rosa S.P. & Dias D. leg. (MZUSP); 1 female, dissected, ravine soil, collected as larva on 24.V.2016, pupa on 3.VIII.2016, adult on 24.VIII.2016, Rosa S.P. & Lopes A.C. leg. (MZUSP); 2 males (dissected, metallized for SEM), 15.X.–8.XI.2015, Rosa S.P. & Dias D. leg. (UNIFEI); 1 male (RK1169, DNA extracted), 15.X.–8.XI.2015, Rosa S.P. & Dias D. leg. (UPOL).

Other material examined. Same locality as for the holotype, ravine soil, 1 pupa (RK1167, DNA extracted), 12.VII.2018, Rosa S.P., Barbosa T. & Ribeiro G. leg. (UPOL); 1 larva, metallized for SEM, 25.VIII.2016, Rosa S.P. & Lopes A.C. leg. (UNIFEI); 1 larva (RK1174, DNA extracted), 12.IV.2018, Rosa S.P., Barbosa T. & Paiva J. leg. (UPOL); same data, 1 larval exuvia (UNIFEI).

Etymology. From Latin; plenus = full, alatus = winged; allusion to complete elytra.

Diagnosis. *Tujamita plenalatum* is the only species in the genus and can be distinguished from other representatives of Jurasaidae by the characters listed in the generic diagnosis.

Description. **Adult male** (Figs 5e,f, S5, S6j–l, S7, S8c). Body length from head to apex of abdomen 3.1–4.5 mm, up to apex of wings 3.2–4.6 mm. Body (Figs 5e,f, S5a–c) in dorsal view (up to visible elytral apex) about 3 times longer than wide. Head, antennomeres I–III and pronotum amber, antennomeres III–XI brown to dark brown, elytra dark brown, with humeri, epipleura, and posterior 2/3 of suture amber; ventral surface of thorax and abdomen brown to dark brown, legs brown with femora amber. Head (Figs S5d, S7a–d) about as long as wide, densely punctate laterally, almost

glabrous at midline, punctures umbilicate; posterior part to eyes as long as anterior part, weakly narrowed posteriad to parallel-sided, posterior margin weakly emarginate; labrum 4 times wider than long, anterior margin rounded; antenna 2/3 as long as distance from head to elytral apices in dorsal view. Mouthparts (Fig. S7d) with maxillary palpus 5-segmented, palpomeres I, III and IV subequal in length, palpomere II shortest, apical palpomere as long as combined lengths of palpomeres III and IV, palpiger with small sclerotized spot; prementum with lateral and anterior edges sclerotized, sparsely setose, labial apical palpomere 6–8 times longer than basal one.

Pronotum (Figs S5d, S6j,k, S7f) 1.5–1.7 times wider than long, lateral margins subparallel, with complete lateral carina visible from above; anterior margin almost straight, posterior margin weakly arcuate, anterior and posterior angles rounded, not produced; finely and sparsely setose-punctate on margins, almost glabrous at middle; prosternal process with apex truncate. Scutellar shield (Fig. S6j) as long as wide, sides rounded or slightly divergent posteriorly, posterior margin straight or rounded, finely setose-punctate; meso-metaventrite suture (Fig. S7f,g) weakly impressed in ventral view, indistinct in lateral view. Elytra (Figs 5e, S5a) parallel-sided, sutures contiguous, apices rounded (apices strongly curved ventrad in both ethanol-preserved and dried specimens); sparsely finely punctate, not separated. Hind wing (Fig. S8c) surpassing elytral apex by 0.2 times elytral length, RA1+2 and RA3+4 not contiguous, r3 elongate, MP1+2, RM loop and medial spur present; MP3+4, Cu, AA3+4 and AP3+4 well impressed, branches indistinct; veins blurred, except SCA, RA and MP1+2; anal field prominent, anal notch present; apical field with three triangular sclerotizations occupying 1/3 of total wing length; apex weakly notched between two apical-most sclerotizations. Abdomen (Fig. S5f) with tergite VIII (Fig. S7j) semi-oval, 1.1 times as long as wide, sclerotized (except for membranous anterior margin), sparsely setose on posterior 2/3; sternite VIII (Fig. S7k) 5.8 times wider than long, partly membranous, with bisinuate sclerotization, hidden under sternite VII; posterior parts of tergites IX and X (Fig. S7l) transverse, membranous, densely covered with microsetae; sternite IX (Fig. S7m) with sclerotized lateral arms subparallel, anterior margin rounded; median part with sides rounded; apical lobes 1/4 as long as sternite IX total length, with very short setae. Aedeagus (Fig. S7o–q) with phallobase about 3 times longer than wide; 1.7 times longer than parameres; parameres rounded laterally (semi-oval), anterior margin rounded or straight; apices curved inwards; anterior half of phallus with sides rounded, posterior half narrowed and parallel-sided, apex rounded in ventral view.

Adult female (Figs 2f, S9). Body length 4.5 mm. Head, antenna, pronotum and leg sclerotized, light brown; mesothorax, metathorax and abdomen larviform, soft and cream-white, moderately densely covered with very short setae. Head (Fig. S9a–c) weakly convex, 1.7 times wider than long, with sides rounded, finely punctate-setose, pilosity sparser at middle; eyes small (ocular index 0.2), circular, not protruded; labrum (Fig. S9e) free, twice wider than long, anterior margin weakly emarginate and densely setose, setae long. Antenna (Fig. S9d) moniliform, with 9 antennomeres, 1.4 times longer than head, setose; antennal insertions elevated. Mandible (Fig. S9f) unidentate, with a few setae laterally at base; prostheca, penicillus and mola absent; cardo, stipes, galea and lacinia indistinct, maxillary palpus 4-segmented, setose; palpiger salient, apical palpomere as long as combined lengths of palpomeres III and IV, somewhat elliptical (asymmetrical) with basiconic sensilla at apex and set of parallel slit-like sensilla laterally. Prementum glabrous, ligula short, labial palpi 2-segmented, apical palpomere fusiform, 3 times longer than basal one, with one seta on inner margin and basiconic sensilla apically, postmentum indistinct. Gular sutures distinct, parallel, gula 1/3 as wide as head, with one pair of setae; posterior tentorial pits absent; cervical

sclerites in two parts. Pronotum (Fig. S9a,c) sclerotized, transverse, 2.5 times wider than long, lateral carina absent, sparsely setose; prosternum reduced to very narrow sclerotized strip. Meso- and metathorax subequal in shape to those of larva. Leg (Fig. S9g) short, sparsely setose, with a few campaniform sensilla, denser on trochanter; trochantin exposed, coxae conical, projecting, trochanter obliquely truncate, 0.5 times as long as femur; femur as long as tibia, tarsus 4-4-4, longer than tibia, tarsomeres subtriangular, I–III subequal in length, each apically obliquely truncate and about half length of tarsomere IV; claws paired, simple, glabrous. Reproductive tract (Fig. S9h) occupying segment VIII; vagina short, spermatheca membranous, colleterial glands with ducts very short.

Mature larva (Figs 2a,b, S10j–m, S11c–e, S12i–l). Body length 4–6 mm. Body (Fig. 2a,b) stout, 5–6 times longer than wide, cream-white. Head (Figs S10j,k, S11c, S12i,j) convex dorsally; dorsal surface with a more sclerotized well defined subtriangular area; clypeolabrum with 4 parasagittal long setae; dorsal posterior margin sinuous, deeply emarginate at middle; ventral posterior margin convergent on lateral 1/3; median 1/3 straight or curved; basal antennomere 1.7–2.0 times longer than apical one. Mandible (Figs S11d,e, S12k,l) oblique, convergent anteriad, apical half abruptly sharpened; basal half triangular, with a narrow membranous strip and a strongly sclerotized arc-shaped hypopharyngeal bracon; posterior sclerotized rods curved; labium (Figs S10l, S12i,j) as long as or slightly longer than maxillary palpus, basiconic sensilla absent; ventral surface grooved at midline. Prothorax 2.0–2.2 times wider than long, 0.5–0.8 times as long as mesothorax, dorsal surface with five setae between lateral edge and lateral stripe (one pair anteriorly, one pair posteriorly, and single seta at midlength), four setae between stripes (one pair anteriorly and one pair posteriorly). Meso-, metathorax, and abdominal segments I–VIII with six setae at midlength (one pair lateral, one pair laterodorsal and one pair parasagittal); ventral surface of abdominal segments I–VIII with six setae at midlength (two pairs ventrolateral and one pair parasagittal). Legs separated by 8–12 times diameter of coxa.

Male pupa (Fig. 3c). Body length 3.0 mm. Frons with four setae (one pair between antennal insertions and one pair posteriorly to antennal insertions); antennal insertions separated by 0.5 times scape width; labrum triangular, glabrous. Pronotum with fine setae; legs glabrous. Abdomen 1.9 times longer than combined head and prothorax; abdominal tergite IX with fine setae lateroposteriorly and 2 short brown spiniform setae at apex.

Female pupa. Body length 4.5 mm. Similar to adult female but tegument entirely smooth and white, leg and mouth joints swollen, inarticulate, legs truncate at apex.

Biological notes. Larvae and male pupae of *Jurasai itajubense* and *Tujamita plenalatum* were found at the same place, i.e., in a single ravine in the Municipal Biological Reserve Serra dos Toledos. Although both species share the same microhabitat, *T. plenalatum* is much more rare; of the total 87 larvae collected, only four belonged to that species. In the laboratory, larvae were individually kept in about 50–80-ml capped plastic pots with soil. One male pupa of *J. itajubense* was collected in the field and all remaining pupae emerged in the laboratory; five of them reached the adult stage. Seven adult females of *J. itajubense* were collected in the field or obtained from larvae in the laboratory. The only male pupa of *T. plenalatum* collected in the field died and was associated with adult and larva of the same species by the molecular analysis (Fig. S14). One female larva of *T. plenalatum* reached the adult stage. The mortality of larvae was very high and most of them, mainly the smallest individuals

(about 2–3 mm), disappeared in the substrate, possibly due to the rapid decomposition. A few larval exuviae were obtained and only two heads could be localized and preserved (Fig. S11b–e).

Larvae were very resistant to dry conditions. Some individuals found in a very dry substrate became thinner and translucent, but still active. After the moisture was re-established, larvae returned to their original size and milky-white coloration. On the other hand, all larvae kept in a very moist soil became swollen, with heads extremely protracted, and eventually died. In both *Jurasai* and *Tujamita*, the pupal stage in females lasts 8–9 days. The female pupae were moveable and capable of walking. They differ from adults by the typically swollen antennae, mouthparts and legs, and unpigmented eyes. All larvae, female pupae and females usually stayed burrowed in the substrate or resting on the surface (Fig. 2). The pupal stage in males of both genera lasts 22–24 days. The adult males usually remained inside the substrate. When unearthed, they walked along the borders of the glass and soon buried themselves again into the substrate. Only once we observed a male shortly jump, trying to fly.

We found no evidence that either larvae or adults feed (e.g., the movement of the mouthparts or signs of pumping into the esophagus). We offered larvae various potential food items, including the plant roots from the collection site, termites, small pieces (or juice) of yams, carrots, potatoes, fruits and hummingbird artificial nectar. When some food was placed very close to the larvae, they continually and intensely twitched but appeared not to be drawn to the food source. However, since many larvae were collected between the roots, their food source is most probably associated with these roots - they possibly feed on juices of fungal hyphae.

Mating was observed in the laboratory (Fig. 4, Supplementary Video). When male and recently emerged females were put together in the same capped plastic pot, the male walked around, touching the substrate with maxillary palps and antennae. For several times, the male walked around the pot, passed over the female and tried to bury himself without showing any interest in the female. In these cases, the females remained resting immobile on the soil surface. About a week after eclosion, the males became very active, walking around, and when touched the female they tried to copulate. Mounted on the female, male touched the body of the female using the aedeagus and the mouthparts until it reached its posterior end, then curved the apex of its abdomen under the female's abdomen, trying to attach the phallus to the ooporus. The apices of the parameres fit above the sclerotized plates of the valves, and the phallus was inserted into the ooporus. The insertion of the phallus into the ooporus was observed several times, each for less than 3 seconds. When males tried to copulate, the females did not seem to be receptive as they always buried themselves. Males usually pulled females with their legs, digging them up. However, the females buried themselves again until the male seemed either to loose interest or the ability to locate her. Some females had the segments VIII and IX more inflated and the ooporus protruding, easily visible. Several weeks after copulation, neither eggs nor larvae were found in the substrate.

References for Supplementary Text

Bocak, L. & Bocakoca, M. Lycidae Laporte, 1836 in *Coleoptera, Beetles; Volume 2: Morphology and Systematics (Elateroidea, Bostrichiformia, Cucujiformia partim)* (eds Leschen, R. A. B., Beutel, R. G. & Lawrence, J. F) in *Handbook of Zoology, Arthropoda: Insecta* (eds Kristensen, N. P. & Beutel, R. G.) 114–122 (Berlin/New York: Walter de Gruyter GmbH & Co., 2010).

Branham, M. A. Lampyridae Latreille, 1817 in *Coleoptera, Beetles; Volume 2: Morphology and Systematics (Elateroidea, Bostrichiformia, Cucujiformia partim)* (eds Leschen, R. A. B., Beutel, R. G. & Lawrence, J. F) in *Handbook of Zoology, Arthropoda: Insecta* (eds Kristensen, N. P. & Beutel, R. G.) 141–148 (Berlin/New York: Walter de Gruyter GmbH & Co., 2010).

Costa, C. & Zaragoza-Caballero, S. Phengodidae LeConte, 1861 in *Coleoptera, Beetles; Volume 2: Morphology and Systematics (Elateroidea, Bostrichiformia, Cucujiformia partim)* (eds Leschen, R. A. B., Beutel, R. G. & Lawrence, J. F) in *Handbook of Zoology, Arthropoda: Insecta* (eds Kristensen, N. P. & Beutel, R. G.) 126–135 (Berlin/New York: Walter de Gruyter GmbH & Co., 2010).

Ramsdale, A.S. Omethidae LeConte, 1861 in *Coleoptera, Beetles; Volume 2: Morphology and Systematics (Elateroidea, Bostrichiformia, Cucujiformia partim)* (eds Leschen, R. A. B., Beutel, R. G. & Lawrence, J. F) in *Handbook of Zoology, Arthropoda: Insecta* (eds Kristensen, N. P. & Beutel, R. G.) 149–152 (Berlin/New York: Walter de Gruyter GmbH & Co., 2010a).

Ramsdale, A.S. Cantharidae Imhoff, 1856 in *Coleoptera, Beetles; Volume 2: Morphology and Systematics (Elateroidea, Bostrichiformia, Cucujiformia partim)* (eds Leschen, R. A. B., Beutel, R. G. & Lawrence, J. F) in *Handbook of Zoology, Arthropoda: Insecta* (eds Kristensen, N. P. & Beutel, R. G.) 153–161 (Berlin/New York: Walter de Gruyter GmbH & Co., 2010b).

Wittmer W. Arbeiten zu einer Revision der Familie Phengodidae (Coleoptera). Entomologische Arbeiten aus dem Museum G. Frey Tutzing bei München 27, 415–524 (1976).

Zaragoza-Caballero, S. Two new genera and three new species of Penicillophorinae (Coleoptera: Phengodidae) of Mexico. *Rev. Mex. Biodivers*. **79**, 363–368 (2008).

Zaragoza-Caballero, S. & Zurita-García, M. L. A preliminary study on the phylogeny of the family Phengodidae (Insecta: Coleoptera). *Zootaxa* **3947**, 527–542 (2015).

Zaragoza-Caballero, S. & Pérez Hernández, C. X. *Sinopsis de la familia Phengodidae (Coleoptera): trenecitos, bigotudos, glow-worms, railroad-worms o besouros trem de ferro*. México, D.F.: Instituto de Biología, Universidad Nacional Autónoma de México (2014).

Table S1. Selected morphological characters of different sexes in Jurasaidae genera.

	Jurasai		*Tujamita*	
	male	female	male	female
**Head**				
Eye	compound	stemma	compound	compound
Antenna	adult-like	larva-like	adult-like	adult-like
	11 antennomeres	4 antennomeres(1)	11 antennomeres	9 antennomeres
	filiform	telescoped	filiform	moniliform
Gular sutures	present	absent	present	present
Tentorial pits	present	absent	present	absent
Labrum	distinct	distinct	distinct	distinct
Maxillary palpus	4 or 5-segmented	4-segmented	5-segmented	4-segmented
**Thorax**				
Pronotum	adult-like	larva-like	adult-like	adult-like(2)
Prosternum	adult-like	larva-like	adult-like	reduced
Mesothorax	adult-like	larva-like	adult-like	larva-like(3,5)
Metathorax	adult-like	larva-like	adult-like	larva-like(3,5)
Leg	adult-like	larva-like(4)	adult-like	adult-like
Tarsus	5-5-5	-	5-5-5	4-4-4
**Abdomen**	adult-like	larva-like	adult-like	larva-like(3,5)

(1) three antennomeres in larva
(2) but lateral carinae absent (present in male)
(3) but different chaetotaxy
(4) but tarsus and tibia separate, with claws paired
(5) but with an ooporus

Table S2. Morphological comparison of Jurasaidae with Elateroidea. The selected characters are compared with those in Lawrence et al. (2011) and Bocak et al. (2016).

Synapomorphies shared with Elateroidea (clade 35 in Lawrence et al. 2011)	
1.	Adult corpotentorial bridge incomplete or absent.
2.	Adult hind wing with apical field without distinct veins, with sclerotizations (Fig. S8).
3.	Larval labral tormae absent (Fig. S11a,c).
4.	Larval maxillary articulating area absent (Fig. S12d,j).
5.	Larval tentorial bridge absent (Fig. S11a,c).
6.	Larva oral cavity blocked, for liquid feeding (Fig. S11a,c).
Autapomorphies of Jurasaidae (new states for characters 399 and 404)	
1.	Larval maxillae and labium fused together and to head capsule (only maxillary palpus free) (Fig. S12d,j).
2.	Larval cardo fused to stipes (Fig. S12d,j).
Characters shared with soft-bodied elateroids (clade 37 in Lawrence et al. 2011)	
1.	Adult mandible more than 2 times as long as basal width (Fig. S6c,g).
2.	Prosternal process incomplete, ending at most at posterior edge of coxa (Fig. S4g).
3.	Procoxae projecting well below prosternum (Fig. S1c).
4.	Elytral apices independently rounded, truncate or emarginate, separated by distinct gap (only in *Jurasai* gen. nov.) (Fig. S1a,f).
5.	Mesocoxae strongly projecting (Fig. S1c).
6.	Angle formed at midline by horizontal line and line tangential to anterior edge of metacoxa at least 30 degrees (Fig. S4f).
7.	Abdominal sternite III without intercoxal process (Figs S1h, S5f).
Characters shared with soft-bodied elateroids (see Bocak et al. 2016)	
1.	Adult procoxal cavities reduced (Fig. S4c).
2.	Lateral portion of the adult prosternum in the front of procoxal base 0.2–0.3 times as long as mid-length of the coxal base at that point (Fig. S4c).
3.	Anterior edge of the adult scutellar shield gradually elevated (Fig. S6j).
4.	Adult anterior process of the metendosternite at least as long as wide (Fig. S3i).
Characters different from soft-bodied elateroids	
1.	Adult mesocoxal cavity distinct (Fig. S4f).
2.	Adult male abdomen with five ventrites (sternites III–VII) (Figs S1h, S5f).
3.	Adult male sternite VIII concealed at least in part by sternite VII (Fig. S1h).
4.	Adult mesoventral process distinct (Fig. S4g,h).

References for Table S2

Bocak, L., Kundrata, R., Andújar-Fernández, C. & Vogler, A. P. The discovery of Iberobaeniidae (Coleoptera: Elateroidea), a new family of beetles from Spain, with immatures detected by environmental DNA sequencing. *Proc. R. Soc. B* **283**, 20152350 (2016).

Lawrence, J. F. et al. Phylogeny of the Coleoptera based on morphological characters of adults and larvae. *Ann. Zool.* **61**, 1–217 (2011).

Table S3. List of taxa used in the analyses, with voucher and GenBank accession numbers. The chimaeric taxa are marked with an asterisk (*).

Superfamily/family	Subfamily	Genus/Species	Geographic origin	Markers 18S	28S	*rrnL*	*cox1*	Specimen voucher	Matrix voucher	Original source
**SCIRTOIDEA**										
Clambidae		gen. sp.	Indonesia	KF625497	KF626105	KF625804	KF625196	UPOL 001320	1320CLA	Kundrata et al. 2014
Eucinetidae		*Eucinetus haemorrhoidalis*	Czech Republic	KF625496	KF626097	KF625806	KF625198	UPOL 001319	1319EUT	Kundrata et al. 2014
Eucinetidae		*Eucinetus* sp.	USA	AF427609	AJ862793	AJ862756	AJ862822	APV–2001	SCIEuEuc	Bocakova et al. 2007
Scirtidae		*Prionocyphon sexmaculatus*	Japan	KF625516	KF626117	KF625822	KF625216	UPOL RK0170	R170SCI	Kundrata et al. 2014
Scirtidae		*Scirtes hemisphaericus*	United Kingdom	AF451937	DQ198699	DQ202592	DQ221997	BMNH 679275	SCIScSci	Bocakova et al. 2007
Scirtidae		*Scirtes* sp.	Japan	KF625500	KF626100	KF625808	KF625201	UPOL RK0160	R160SCI	Kundrata et al. 2014
Scirtidae		*Cyphon hilaris*	United Kingdom	AF201419	DQ198698	DQ198620	DQ198542	BMNH 679123	SCIScCyp	Bocakova et al. 2007
Scirtidae		*Cyphon* sp.	Cameroon	KF625501	KF626101	KF625809	KF625202	UPOL RK0161	R161SCI	Kundrata et al. 2014
Scirtidae		gen. sp.	Malaysia	KF625505	KF626106	KF625813	KF625206	UPOL 001321	1321SCI	Kundrata et al. 2014
Scirtidae		gen. sp.	Ethiopia	KF625498	KF626098	KF625807	KF625199	UPOL RK0147	R147SCI	Kundrata et al. 2014
Scirtidae		gen. sp.	Indonesia	KF625502	KF626102	KF625810	KF625203	UPOL RK0162	R162SCI	Kundrata et al. 2014
Scirtidae		gen. sp.	Philippines	KF625504	KF626104	KF625812	KF625205	UPOL RK0165	R165SCI	Kundrata et al. 2014
Scirtidae		gen. sp.	Cameroon	KX092896	KX093035	KX092644	KX092758	UPOL RK0641	R641SCI	Kundrata et al. 2017
Scirtidae		gen. sp.	Cameroon	KX092897	KX093036	KX092645	KX092759	UPOL RK0642	R642SCI	Kundrata et al. 2017
Scirtidae		gen. sp.	Indonesia	KX092895	KX093034	KX092643	KX092761	UPOL RK0698	R698SCI	Kundrata et al. 2017
**DASCILLOIDEA**										
Dascillidae	Dascillinae	*Dascillus cervinus*	United Kingdom	AY745558	DQ198700	DQ198621	DQ198543	BMNH 679199	DASDaDas	Bocakova et al. 2007
Dascillidae	Dascillinae	*Petalon* sp.	Laos	KX092899	N	KX092646	KX092763	UPOL RK0639	R639DAS	Kundrata et al. 2017
Dascillidae	Karumiinae	*Genecerus* cf. *cervinus*	Oman	KX092900	KX093038	KX092647	KX092764	UPOL RK0793	R793DAS	Kundrata et al. 2017
Dascillidae	Karumiinae	*Emmita serricornis*	Morocco	N	N	N	KX092765	UPOL RK0791	R791DAS	Kundrata et al. 2017
Rhipiceridae	Sandalinae	*Sandalus* sp.	South Africa	KF625506	KF626108	KF625814	KF625208	UPOL RK0144	R144RHI	Kundrata et al. 2014
Rhipiceridae	Sandalinae	*Sandalus* sp.	Zambia	KF625507	KF626107	KF625815	KF625207	UPOL 001322	1322RHI	Kundrata et al. 2014
**BUPRESTOIDEA**										
Schizopodidae	Schizopodinae	*Glyptoscelimorpha marmorata*	USA	KM364156	KM364300	N	KM364418	BUP0298	SCHglyp	Evans et al. 2015
Schizopodidae	Schizopodinae	*Schizopus laetus*	USA	KM364083	KM364209	N	KM364343	BUP0272	SCHschl	Evans et al. 2015
Buprestidae	Agrilinae	*Agrilus* sp.	UK/Czech Rep.	AF451934	DQ198701	DQ198622	DQ198544	UPOL 001047	BUPBuAgr	Bocakova et al. 2007
Buprestidae	Agrilinae	*Mychommatus violaceus*	Cameroon	KX092902	KX093039	KX092648	KX092767	UPOL RK0777	R777BUP	Kundrata et al. 2017
Buprestidae	Agrilinae	*Sibuyanella bakeri*	Philippines	KX092903	KX093040	KX092649	KX092768	UPOL RK0778	R778BUP	Kundrata et al. 2017
Buprestidae	Agrilinae	*Agrilus* sp.	Cameroon	KX092904	KX093041	KX092650	KX092769	UPOL RK0783	R783BUP	Kundrata et al. 2017
Buprestidae	Agrilinae	*Pseudagrilus granulosus*	Ethiopia	KX092907	KX093043	KX092652	KX092772	UPOL RK0784	R784BUP	Kundrata et al. 2017
Buprestidae	Agrilinae	*Aphanisticus* sp.	Cameroon	KX092908	KX093044	KX092653	KX092773	UPOL RK0785	R785BUP	Kundrata et al. 2017
Buprestidae	Agrilinae	*Pseudagrilus alutaceus*	Ethiopia	KX092909	KX093045	KX092654	KX092774	UPOL RK0786	R786BUP	Kundrata et al. 2017
Buprestidae	Agrilinae	*Aphanisticus elongatus*	Italy	KX092914	KX093048	KX092657	KX092779	UPOL RK0801	R801BUP	Kundrata et al. 2017
Buprestidae	Agrilinae	*Habroloma subbicorne*	China	KX092915	KX093049	KX092658	KX092780	UPOL RK0802	R802BUP	Kundrata et al. 2017
Buprestidae	Agrilinae	*Trachys saundersi*	China	KX092917	KX093051	KX092660	KX092782	UPOL RK0804	R804BUP	Kundrata et al. 2017
Buprestidae	Agrilinae	*Coraebus diminutus*	China	KX092923	KX093057	KX092666	KX092788	UPOL RK0805	R805BUP	Kundrata et al. 2017
Buprestidae	Agrilinae	*Meliboeus parvulus*	Turkey	KX092929	KX093063	KX092672	KX092794	UPOL RK0808	R808BUP	Kundrata et al. 2017
Buprestidae	Agrilinae	*Meliboeus* sp.	Ethiopia	KX092930	KX093064	KX092673	KX092795	UPOL RK0814	R814BUP	Kundrata et al. 2017
Buprestidae	Agrilinae	*Meliboeus burgeoni*	Cameroon	KX092931	KX093065	KX092674	KX092796	UPOL RK0815	R815BUP	Kundrata et al. 2017
Buprestidae	Agrilinae	*Agrilus* sp.	China	KX092932	KX093066	KX092675	KX092797	UPOL RK0823	R823BUP	Kundrata et al. 2017
Buprestidae	Buprestinae	*Anthaxia hungarica*	France	DQ100484	DQ198702	DQ198623	DQ198545	UPOL 000M24	BUPBuAnt	Bocakova et al. 2007
Buprestidae	Buprestinae	*Chrysobothris* sp.	Malaysia	KX092926	KX093060	KX092669	KX092791	UPOL RK0813	R813BUP	Kundrata et al. 2017
Buprestidae	Buprestinae	*Anthaxia corinthia*	Cyprus	KX092919	KX093053	KX092662	KX092784	UPOL RK0807	R807BUP	Kundrata et al. 2017
Buprestidae	Buprestinae	*Megactenodes levior*	Cameroon	KX092921	KX093055	KX092664	KX092786	UPOL RK0779	R779BUP	Kundrata et al. 2017
Buprestidae	Chrysochroinae	*Sphenoptera tappesi*	Turkey	KX092925	KX093059	KX092668	KX092790	UPOL RK0812	R812BUP	Kundrata et al. 2017
Buprestidae	Chrysochroinae	*Sphenoptera coracina*	Turkey	KX092928	KX093062	KX092671	KX092793	UPOL RK0824	R824BUP	Kundrata et al. 2017
Buprestidae	Chrysochroinae	*Capnodis tenebricosa*	Greece	KX092920	KX093054	KX092663	KX092785	UPOL RK0781	R781BUP	Kundrata et al. 2017
Buprestidae	Chrysochroinae	*Chalcophorella stigmatica*	Turkey	KX092922	KX093056	KX092665	KX092787	UPOL RK0797	R797BUP	Kundrata et al. 2017
Buprestidae	Julodinae	gen. sp.	South Africa	AF451935	DQ198703	DQ198624	DQ198546	BMNH 679324	BUPBuJul	Bocakova et al. 2007
Buprestidae	Julodinae	*Julodis pubescens*	Greece, Crete	KX092911	KX093046	KX092655	KX092776	UPOL RK0798	R798BUP	Kundrata et al. 2017
Buprestidae	Polycestinae	*Acmaeodera lugubris*	Spain	KX092924	KX093058	KX092667	KX092789	UPOL RK0809	R809BUP	Kundrata et al. 2017

Buprestidae	Polycestinae	*Astraeus irregularis*	Australia	KM364139	KM364283	N	KM364404	BUP0008	BUP0008	Evans et al. 2015
Buprestidae	Polycestinae	*Polycesta aruensis*	USA	KM364149	KM364293	N	KM364413	BUP0060	BUP0060	Evans et al. 2015
**BYRRHOIDEA**										
Byrrhidae	Byrrhinae	*Byrrhus pilula*	United Kingdom	AF427604	DQ198705	DQ198625	DQ198548	BMNH 679172	BYRByByr	Bocakova et al. 2007
Byrrhidae	Byrrhinae	*Byrrhus* sp.	France	KX092934	KX093068	N	KX092799	UPOL RK0651	R651BYR	Kundrata et al. 2017
Byrrhidae	Byrrhinae	*Curimus erichsoni*	Czech Republic	KX092935	KX093069	KX092676	KX092800	UPOL RK0652	R652BYR	Kundrata et al. 2017
Byrrhidae	Byrrhinae	*Simplocaria* sp.	–	KM364160	KM364303	N	N	BT0017	BYRsimp	Evans et al. 2015
Byrrhidae		*Chalcosphaerium* sp.	–	EF214158	N	EF214030	EF214233	BMNH 668332	BYRch02	Hunt et al. 2007
Byrrhidae	Syncalyptinae	*Chaetophora spinosa*	United Kingdom	AF451929	DQ198706	N	N	BMNH 679203	BYRByCha	Bocakova et al. 2007
Byrrhidae	Syncalyptinae	*Curimopsis setigera*	United Kingdom	AF451930	DQ198707	N	N	BMNH 679204	BYRByCur	Bocakova et al. 2007
Callirhipidae		*Callirhipis dissimilis*	Malaysia	KX092944	KX093078	KX092685	KX092809	UPOL RK0643	R643CAL	Kundrata et al. 2017
Callirhipidae		*Callirhipis suturalis*	Malaysia	KX092942	KX093076	KX092683	KX092807	UPOL RK0644	R644CAL	Kundrata et al. 2017
Callirhipidae		*Callirhipis* sp.	Indonesia	KX092943	KX093077	KX092684	KX092808	UPOL RK0655	R655CAL	Kundrata et al. 2017
Callirhipidae		*Ennometes* cf. *testaceicornis*	Malaysia	KX092946	KX093080	KX092686	KX092810	UPOL RK0645	R645CAL	Kundrata et al. 2017
Callirhipidae		gen. sp.	Malaysia	DQ100490	DQ198726	DQ198637	DQ198560	UPOL 000M23	BYRCaCal	Bocakova et al. 2007
Callirhipidae		gen. sp.	Malaysia	KF625511	KF626112	KF625818	KF625211	UPOL 001249	1249CAL	Kundrata et al. 2014
Chelonariidae		gen. sp.	Indonesia	DQ100488	DQ198724	DQ198635	DQ198558	UPOL 000M06	BYRChChe	Bocakova et al. 2007
Chelonariidae		gen. sp.	Indonesia	KF625509	KF626110	KF625817	KF625210	UPOL 001324	1324CHE	Kundrata et al. 2014
Chelonariidae		gen. sp.	China	KX092996	KX093124	KX092731	KX092857	UPOL RK0704	R704CHE	Kundrata et al. 2017
Chelonariidae		gen. sp.	China	KX092997	KX093125	KX092732	KX092858	UPOL RK0705	R705CHE	Kundrata et al. 2017
Chelonariidae		gen. sp.	China	KX092998	KX093126	KX092733	KX092859	UPOL RK0706	R706CHE	Kundrata et al. 2017
Dryopidae	*incertae sedis*	*Ceradryops matei*	–	EF209494	N	EF209434	EF209554	FC_C12	DRYCema	Ciampor & Ribera
Dryopidae		*Pomatinus substriatus*	United Kingdom	AF451924	DQ198708	DQ198626	DQ198549	BMNH 693616	BYRDrPom	Bocakova et al. 2007
Dryopidae		*Dryops* sp.	Madagascar	KX092971	KX093101	KX092709	KX092835	UPOL RK0672	R672DRY	Kundrata et al. 2017
Dryopidae		*Sostea* sp.	Cambodia	KX092983	KX093112	KX092721	KX092846	UPOL RK0674	R674DRY	Kundrata et al. 2017
Dryopidae		*Sostea* sp.	Malaysia	KX092989	KX093118	KX092725	KX092851	UPOL RK0676	R676DRY	Kundrata et al. 2017
Dryopidae		*Sostea* sp.	Malaysia	KX092985	KX093114	KX092723	KX092847	UPOL RK0677	R677DRY	Kundrata et al. 2017
Dryopidae		*Sostea* sp.	Indonesia	KX092987	KX093116	KX092724	KX092849	UPOL RK0679	R679DRY	Kundrata et al. 2017
Dryopidae		*Sostea* sp.	Indonesia	KX092977	KX093107	KX092715	KX092841	UPOL RK0681	R681DRY	Kundrata et al. 2017
Dryopidae		*Sostea* sp.	Indonesia	KX092980	KX093110	KX092718	KX092843	UPOL RK0683	R683DRY	Kundrata et al. 2017
Dryopidae		*Sostea* sp.	Indonesia	KX092982	KX093111	KX092720	KX092845	UPOL RK0686	R686DRY	Kundrata et al. 2017
Dryopidae		*Sostea* sp.	Philippines	KX092990	KX093119	KX092726	KX092852	UPOL RK0687	R687DRY	Kundrata et al. 2017
Dryopidae		*Sostea* sp.	Philippines	KX092992	KX093120	KX092728	KX092854	UPOL RK0688	R688DRY	Kundrata et al. 2017
Dryopidae		*Sostea* sp.	China	KX092979	KX093109	KX092717	KX092842	UPOL RK0692	R692DRY	Kundrata et al. 2017
Dryopidae		*Dryops lutulentus*	France	KX092976	KX093106	KX092714	KX092840	UPOL RK0718	R718DRY	Kundrata et al. 2017
Dryopidae		gen. sp.	Indonesia	KX092972	KX093102	KX092710	KX092836	UPOL RK0733	R733DRY	Kundrata et al. 2017
Dryopidae		gen. sp.	Cameroon	KX092995	KX093123	KX092730	KX092856	UPOL RK0738	R738DRY	Kundrata et al. 2017
Dryopidae		*Pachyparnus* sp.	Indonesia	KX092973	KX093103	KX092711	KX092837	UPOL RK0740	R740DRY	Kundrata et al. 2017
Dryopidae		gen. sp.	Indonesia	KX092974	KX093104	KX092712	KX092838	UPOL RK0742	R742DRY	Kundrata et al. 2017
Dryopidae		*Pachyparnus* sp.	Malaysia	KX092975	KX093105	KX092713	KX092839	UPOL RK0745	R745DRY	Kundrata et al. 2017
Elmidae	Elminae	*Limnius perrisi*	–	AF451915	AJ862767	AJ862736	AJ862800	BMNH 693613	BYRElLip	Bocakova et al. 2007
Elmidae	Elminae	*Macronychus quadrituberculatus*	Hungary	AF451920	DQ198713	EF209458	EF209578	BMNH 693611	BYRElMac	Ciampor & Ribera
Elmidae	Elminae	*Oulimnius rivularis*	Portugal	AF451913	DQ198714	DQ198628	DQ198551	BMNH 679264	BYRElOul	Bocakova et al. 2007
Elmidae	Elminae	*Stenelmis* sp.	China	KX093003	KX093131	N	KX092864	UPOL RK0713	R713ELM	Kundrata et al. 2017
Elmidae	Elminae	*Stenelmis* sp.	Malaysia	KX093004	KX093132	KX092737	KX092865	UPOL RK0720	R720ELM	Kundrata et al. 2017
Elmidae	Elminae	*Stenelmis* sp.	Malaysia	KX093005	KX093133	N	KX092866	UPOL RK0721	R721ELM	Kundrata et al. 2017
Elmidae	Elminae	*Stenelmis* sp.	Malaysia	KX093006	KX093134	N	KX092867	UPOL RK0722	R722ELM	Kundrata et al. 2017
Elmidae	Elminae	*Stenelmis* sp.	Malaysia	KX093008	KX093135	EF209466	KX092869	UPOL RK0724/FC19*	R724ELM	Kundrata et al. 2017
Elmidae	Elminae	*Nesonychus* sp.	Indonesia	KX093012	KX093138	KX092741	KX092873	UPOL RK0736	R736ELM	Kundrata et al. 2017
Elmidae	Elminae	*Stenelmis* sp.	Indonesia	KX093013	KX093139	N	KX092874	UPOL RK0737	R737ELM	Kundrata et al. 2017
Elmidae	Elminae	*Ancyronyx raffaelacatharina*	Indonesia	KX093016	KX093141	KX092744	KX092877	UPOL RK0743	R743ELM	Kundrata et al. 2017
Elmidae	Elminae	*Stenelmis* sp.	Indonesia	KX093017	KX093142	N	KX092878	UPOL RK0744	R744ELM	Kundrata et al. 2017
Elmidae	Elminae	*Stenelmis* sp.	Malaysia	KX093018	KX093143	KX092745	KX092879	UPOL RK0746	R746ELM	Kundrata et al. 2017
Elmidae	Elminae	*Stenelmis* sp.	Malaysia	KX093019	KX093144	N	KX092880	UPOL RK0747	R747ELM	Kundrata et al. 2017
Elmidae	Larainae	*Dryopomorphus* sp.	Indonesia	KX093010	KX093137	KX092740	KX092871	UPOL RK0732	R732ELM	Kundrata et al. 2017
Elmidae	Larainae	*Dryopomorphus* sp.	Malaysia	KX093000	KX093128	KX092734	KX092861	UPOL RK0717	R717ELM	Kundrata et al. 2017

Elmidae	Larainae	*Potamodytes* sp.	South Africa	AF451912	DQ198715	DQ198629	DQ198552	BMNH 679360	BYRElPos	Bocakova et al. 2007
Elmidae	Larainae	*Potamodytes* sp.	Madagascar	KX093001	KX093129	KX092735	KX092862	UPOL RK0659	R659ELM	Kundrata et al. 2017
Elmidae	Larainae	*Potamodytes* sp.	Madagascar	KX093002	KX093130	KX092736	KX092863	UPOL RK0660	R660ELM	Kundrata et al. 2017
Elmidae	Larainae	*Potamodytes* sp.	Zambia	KX093014	KX093140	KX092742	KX092875	UPOL RK0739	R739ELM	Kundrata et al. 2017
Eulichadidae		*Eulichas* sp.	Malaysia	DQ100489	DQ198725	DQ198636	DQ198559	UPOL 000M22	BYREuEul	Bocakova et al. 2007
Eulichadidae		*Eulichas* cf. *fasciolata*	Malaysia	KX092937	KX093071	KX092678	KX092802	UPOL RK0646	R646EUL	Kundrata et al. 2017
Eulichadidae		*Eulichas dudgeoni*	China	KX092936	KX093070	KX092677	KX092801	UPOL RK0647	R647EUL	Kundrata et al. 2017
Eulichadidae		*Eulichas funebris*	China	KX092938	KX093072	KX092679	KX092803	UPOL RK0648	R648EUL	Kundrata et al. 2017
Eulichadidae		*Eulichas funebris*	China	KX092939	KX093073	KX092680	KX092804	UPOL RK0649	R649EUL	Kundrata et al. 2017
Eulichadidae		*Eulichas* sp.	China	KX092941	KX093075	KX092682	KX092806	UPOL RK0650	R650EUL	Kundrata et al. 2017
Eulichadidae		*Eulichas baeri*	Philippines	KX092940	KX093074	KX092681	KX092805	UPOL RK0653	R653EUL	Kundrata et al. 2017
Heteroceridae	Heterocerinae	gen. sp.	Indonesia	KX093031	KX093156	KX092756	KX092892	UPOL RK0661	R661HET	Kundrata et al. 2017
Heteroceridae	Heterocerinae	gen. sp.	Malaysia	KX093032	KX093157	KX092757	KX092893	UPOL RK0662	R662HET	Kundrata et al. 2017
Heteroceridae	Heterocerinae	*Augyles maritimus*	Morocco	AF451927	DQ198717	N	N	BMNH 693618	BYRHeAug	Bocakova et al. 2007
Heteroceridae	Heterocerinae	*Heterocerus* sp.	Slovakia	AF451928	DQ198718	DQ198630	DQ198553	UPOL 001048*	BYRHeHet	Bocakova et al. 2007
Limnichidae	Cephalobyrrhinae	*Cephalobyrrhus* sp.	–	EF209534	N	EF209474	EF209594	FC_396	LIMceph	Ciampor & Ribera
Limnichidae	Thaumastodinae	*Pseudeucinetus* sp.	Malaysia	KF625495	KF626096	KF625805	KF625197	UPOL 001318	1318LIM	Kundrata et al. 2014
Limnichidae	Limnichinae	*Limnichus pygmaeus*	United Kingdom	AF451923	DQ198719	DQ198631	DQ198554	BMNH 679196	BYRLiLim	Bocakova et al. 2007
Limnichidae	Limnichinae	*Limnichus* sp.	Indonesia	KX093022	KX093147	KX092748	KX092883	UPOL RK0666	R666LIM	Kundrata et al. 2017
Limnichidae	Limnichinae	*Limnichus* sp.	Malaysia	KX093025	KX093150	KX092751	KX092886	UPOL RK0725	R725LIM	Kundrata et al. 2017
Limnichidae	Limnichinae	*Eulimnichus* sp.	–	AF451922	HQ634240	N	N	IR2002/979750*	LIMeuli	Ribera et al.
Limnichidae	Limnichinae	*Pelochares* sp.	China	KX093024	KX093149	KX092750	KX092885	UPOL RK0708	R708LIM	Kundrata et al. 2017
Limnichidae	Limnichinae	*Pelochares* sp.	Indonesia	KX093023	KX093148	KX092749	KX092884	UPOL RK0665	R665LIM	Kundrata et al. 2017
Limnichidae	Limnichinae	*Byrrhinus* sp.	Cameroon	KX093021	KX093146	KX092747	KX092882	UPOL RK0663	R663LIM	Kundrata et al. 2017
Limnichidae	Limnichinae	*Byrrhinus* sp.	Indonesia	KX093028	KX093153	KX092753	KX092889	UPOL RK0664	R664LIM	Kundrata et al. 2017
Limnichidae	Limnichinae	*Byrrhinus* sp.	Malaysia	KX093027	KX093152	KX092752	KX092888	UPOL RK0727	R727LIM	Kundrata et al. 2017
Limnichidae	Limnichinae	*Mandersia* sp.	Indonesia	KX093029	KX093154	KX092754	KX092890	UPOL RK0667	R667LIM	Kundrata et al. 2017
Limnichidae	Limnichinae	*Mandersia* sp.	China	KX093030	KX093155	KX092755	KX092891	UPOL RK0670	R670LIM	Kundrata et al. 2017
Limnichidae	Limnichinae	*Tricholimnichus* sp.	Malaysia	KX093026	KX093151	N	KX092887	UPOL RK0748	R748LIM	Kundrata et al. 2017
Limnichidae	Limnichinae	*Paralimnichus* sp.	Philippines	KX093020	KX093145	KX092746	KX092881	UPOL RK0691	R691LIM	Kundrata et al. 2017
Lutrochidae		*Lutrochus* sp.	–	EF209539	HQ634239	EF209479	EF209599	FC_C14/JK–2011*	LUTlutr	Ciampor & Ribera
Psephenidae	Eubrianacinae	*Eubrianax* sp.	Indonesia	DQ100485	DQ198721	DQ198632	DQ198555	UPOL 000M33	BYRPsEus	Bocakova et al. 2007
Psephenidae	Eubrianacinae	gen. sp.	Cambodia	KF625503	KF626103	KF625811	KF625204	UPOL RK0163	R163PSE	Kundrata et al. 2014
Psephenidae	Eubriinae	*Dicranopselaphus* sp.	Malaysia	KF625514	KF626115	KF625820	KF625214	UPOL RK0168	R168PSE	Kundrata et al. 2014
Psephenidae	Eubriinae	*Dicranopselaphus* sp.	Indonesia	KF625515	KF626116	KF625821	KF625215	UPOL RK0169	R169PSE	Kundrata et al. 2014
Psephenidae	Eubriinae	*Dicranopselaphus* sp.	China	KX092951	KX093082	KX092691	KX092811	UPOL RK0671	R671PSE	Kundrata et al. 2017
Psephenidae	Eubriinae	*Dicranopselaphus* sp.	Indonesia	KX092949	N	KX092689	KX092812	UPOL RK0699	R699PSE	Kundrata et al. 2017
Psephenidae	Eubriinae	gen. sp.	Indonesia	KX092947	N	KX092687	KX092813	UPOL RK0693	R693PSE	Kundrata et al. 2017
Psephenidae	Eubriinae	*Schinostethus brevis*	Japan	KX092950	KX093081	KX092690	KX092814	UPOL RK0694	R694PSE	Kundrata et al. 2017
Psephenidae	Eubriinae	gen. sp.	Indonesia	KX092948	N	KX092688	KX092815	UPOL RK0700	R700PSE	Kundrata et al. 2017
Ptilodactylidae	Cladotominae	*Paralichas pectinatus*	Japan	DQ100486	DQ198722	DQ198633	DQ198556	UPOL 000M41	BYRPtPar	Bocakova et al. 2007
Ptilodactylidae	Anchytarsinae	gen. sp.	Cameroon	KX092963	KX093094	KX092702	KX092827	UPOL RK0773	R773PTI	Kundrata et al. 2017
Ptilodactylidae	Anchytarsinae	*Epilichas* sp.	Japan	KX092968	KX093099	KX092706	KX092832	UPOL RK0830	R830PTI	Kundrata et al. 2017
Ptilodactylidae	Anchytarsinae	gen. sp.	Cameroon	KX092962	KX093093	KX092701	KX092826	UPOL RK0771	R771PTI	Kundrata et al. 2017
Ptilodactylidae	Anchytarsinae	*Daemon* sp.	Madagascar	KF625517	KF626118	KF625823	KF625222	UPOL RK0131	R131PTI	Kundrata et al. 2014
Ptilodactylidae	Ptilodactylinae	*Ptilodactyla serricornis*	Japan	AF451931	DQ198723	DQ198634	DQ198557	BMNH 693606	BYRPtPti	Bocakova et al. 2007
Ptilodactylidae	Ptilodactylinae	*Ptilodactyla* sp.	China	KX092954	KX093085	KX092694	KX092818	UPOL RK0673	R673PTI	Kundrata et al. 2017
Ptilodactylidae	Ptilodactylinae	*Ptilodactyla* sp.	China	KX092956	KX093087	KX092696	KX092820	UPOL RK0756	R756PTI	Kundrata et al. 2017
Ptilodactylidae	Ptilodactylinae	*Ptilodactyla* sp.	Japan	KX092969	KX093100	KX092707	KX092833	UPOL RK0831	R831PTI	Kundrata et al. 2017
Ptilodactylidae	Ptilodactylinae	gen. sp.	Indonesia	KF625518	KF626119	KF625824	KF625217	UPOL RK0149	R149PTI	Kundrata et al. 2014
Ptilodactylidae	Ptilodactylinae	gen. sp.	Indonesia	KF625520	KF626121	KF625825	KF625219	UPOL RK0151	R151PTI	Kundrata et al. 2014
Ptilodactylidae	Ptilodactylinae	gen. sp.	Indonesia	KF625522	KF626123	KF625827	KF625221	UPOL RK0153	R153PTI	Kundrata et al. 2014
Ptilodactylidae	Ptilodactylinae	gen. sp.	Malaysia	KX092953	KX093084	KX092693	KX092817	UPOL RK0714	R714PTI	Kundrata et al. 2017
Ptilodactylidae	Ptilodactylinae	gen. sp.	China	KX092955	KX093086	KX092695	KX092819	UPOL RK0755	R755PTI	Kundrata et al. 2017
Ptilodactylidae	Ptilodactylinae	gen. sp.	Malaysia	KX092957	KX093088	KX092697	KX092821	UPOL RK0759	R759PTI	Kundrata et al. 2017
Ptilodactylidae	Ptilodactylinae	gen. sp.	Indonesia	KX092958	KX093089	KX092698	KX092822	UPOL RK0760	R760PTI	Kundrata et al. 2017

Ptilodactylidae	Ptilodactylinae	gen. sp.	Malaysia	KX092959	KX093090	KX092699	KX092823	UPOL RK0761	R761PTI	Kundrata et al. 2017
Ptilodactylidae	Ptilodactylinae	gen. sp.	Cameroon	KX092952	KX093083	KX092692	KX092816	UPOL RK0775	R775PTI	Kundrata et al. 2017
Ptilodactylidae	Ptilodactylinae	*Pherocladus* sp.	Indonesia	KF625521	KF626122	KF625826	KF625220	UPOL RK0152	R152PTI	Kundrata et al. 2014
Ptilodactylidae	Ptilodactylinae	*Pherocladus* sp.	Malaysia	KX092961	KX093092	KX092700	KX092825	UPOL RK0764	R764PTI	Kundrata et al. 2017
Ptilodactylidae	Ptilodactylinae	*Pherocladus* sp.	Philippines	KX092964	KX093095	KX092703	KX092828	UPOL RK0826	R826PTI	Kundrata et al. 2017
Ptilodactylidae	Ptilodactylinae	*Pherocladus* sp.	Philippines	KX092965	KX093096	KX092704	KX092829	UPOL RK0827	R827PTI	Kundrata et al. 2017
**ELATEROIDEA**										
Artematopodidae	Artematopodinae	*Eurypogon japonicus*	Japan	KF294761	KF294767	KF294755	KF294774	UPOL RK0091	R091ART	Kundrata et al. 2013
Omethidae	Telegeusinae	*Telegeusis nubifer*	USA	DQ100503	DQ198751	DQ198660	DQ198582	UPOL 000321	telTele	Bocakova et al. 2007
Omethidae	Driloniinae	*Drilonius striatulus*	Japan	KF625527	KF626128	KF625830	KF625227	UPOL 001272	omeDril	Kundrata et al. 2014
Omethidae	Omethinae	*Troglomethes leechi*	USA	KF625529	KF626130	KF625828	KF625229	UPOL 001340	1340OMEo	Kundrata et al. 2014
Omethidae	Matheteinae	*Ginglymocladus* sp.	USA	KF625530	KF626131	KF625829	KF625230	UPOL 001341	1341OMEm	Kundrata et al. 2014
Cerophytidae		*Cerophytum elateroides*	Slovakia	KF625714	KF626302	KF626002	KF625407	UPOL RK0129	R129CER	Kundrata et al. 2014
Jurasaidae		*Jurasai itajubense* gen. et sp. nov.	Brazil	MN583368	MN583350	MN583359	MN578248	UPOL RK1166	RK1166	present study
Jurasaidae		*Jurasai itajubense* gen. et sp. nov.	Brazil	MN583369	MN583351	MN583360	MN578249	UPOL RK1168	RK1168	present study
Jurasaidae		*Jurasai itajubense* gen. et sp. nov.	Brazil	N	MN583352	MN583361	MN578250	UPOL RK1170	RK1170	present study
Jurasaidae		*Jurasai itajubense* gen. et sp. nov.	Brazil	MN583370	MN583353	MN583362	MN578251	UPOL RK1172	RK1172	present study
Jurasaidae		*Jurasai itajubense* gen. et sp. nov.	Brazil	MN583371	MN583354	MN583363	MN578252	UPOL RK1173	RK1173	present study
Jurasaidae		*Jurasai itajubense* gen. et sp. nov.	Brazil	MN583372	MN583355	MN583364	MN578253	UPOL RK1175	RK1175	present study
Jurasaidae		*Jurasai digitusdei* gen. et sp. nov.	Brazil	MN583375	MN583358	N	N	UPOL RK1171	RK1171	present study
Jurasaidae		*Tujamita plenalatum* gen. et sp. nov.	Brazil	N	N	MN583365	MN578254	UPOL RK1167	RK1167	present study
Jurasaidae		*Tujamita plenalatum* gen. et sp. nov.	Brazil	MN583373	MN583356	MN583366	MN578255	UPOL RK1169	RK1169	present study
Jurasaidae		*Tujamita plenalatum* gen. et sp. nov.	Brazil	MN583374	MN583357	MN583367	MN578256	UPOL RK1174	RK1174	present study
Throscidae		gen. sp.	Indonesia	KF625533	KF626134	KF625836	KF625233	UPOL RK0138	R138THR	Kundrata et al. 2014
Throscidae		gen. sp.	Japan	KF625535	KF626136	KF625837	KF625235	UPOL RK0141	R141THR	Kundrata et al. 2014
Eucnemidae	Anischiinae	*Anischia bicolor*	New Caledonia	KF625546	KF626146	KF625846	KF625247	UPOL RK0120	R120EUCa	Kundrata et al. 2014
Eucnemidae	Eucneminae	gen. sp.	Indonesia	HQ333829	HQ333923	HQ333736	HQ334009	UPOL RK0076	R076EUC	Kundrata Bocak 2011
Eucnemidae	Eucneminae	gen. sp.	Indonesia	HQ333830	HQ333924	HQ333737	HQ334010	UPOL RK0077	R077EUC	Kundrata Bocak 2011
Eucnemidae	Macraulacinae	gen. sp.	Japan	KF625564	KF626164	KF625862	KF625265	UPOL RK0350	R350EUC	Kundrata et al. 2014
Eucnemidae	Melasinae	*Micorhagus pygmaeus*	Czech Republic	KF625570	KF626170	KF625867	KF625271	UPOL 001224	1224EUC	Kundrata et al. 2014
Eucnemidae	Melasinae	gen. sp.	Indonesia	KF625549	KF626149	KF625849	KF625250	UPOL RK0123	R123EUC	Kundrata et al. 2014
Eucnemidae	indet.	gen. sp.	Panama	KF625554	KF626154	KF625854	KF625255	UPOL RK0303	R303EUC	Kundrata et al. 2014
Iberobaeniidae		*Iberobaenia minuta*	Spain	KT339296	KT339297	KT825140	KT339298	UPOL RK0790*	IBEsp	Bocak et al. 2016
Lycidae	Libnetinae	*Libnetis* sp.	Malaysia	DQ181038	DQ181112	DQ180964	DQ181186	UPOL 000L02	LLibl1	Bocak et al. 2008
Lycidae	Dictyopterinae	*Lycoprogenthes* sp.	Indonesia	DQ181070	DQ181144	DQ180996	DQ181218	UPOL 000358	LDicly1	Bocak et al. 2008
Lycidae	Dictyopterinae	*Taphes brevicollis*	Laos	DQ181098	DQ181172	DQ181024	DQ181246	UPOL 000812	LDictap	Bocak et al. 2008
Lycidae	Dictyopterinae	*Benibotarus nigripennis*	Japan	DQ181075	DQ181149	DQ181001	DQ181223	UPOL 000572	LDicben	Bocak et al. 2008
Lycidae	Lyropaeinae	*Lyropaeus* sp.	Malaysia	DQ181042	DQ181116	DQ180968	DQ181190	UPOL 000L11	LLyrly1	Bocak et al. 2008
Lycidae	Lyropaeinae	*Antennolycus constrictus*	Malaysia	DQ181051	DQ181125	DQ180977	DQ181199	UPOL 000L22	LLyrana	Bocak et al. 2008
Lycidae	Lyropaeinae	*Platerodrilus* sp.	Malaysia	DQ181037	DQ181111	DQ180963	DQ181185	UPOL 000L01	LLyrmd1	Bocak et al. 2008
Lycidae	Ateliinae	*Scarelus* sp.	Malaysia	DQ181046	DQ181120	DQ180972	DQ181194	UPOL 000L15	LAtesc1	Bocak et al. 2008
Lycidae	Lycinae	*Plateros* sp.	French Guyana	KF625685	KF626271	KF625975	KF625384	UPOL RK0377	R377LYC	Kundrata et al. 2014
Lycidae	Lycinae	*Macrolycus* sp.	China	DQ181049	DQ181123	DQ180975	DQ181197	UPOL 000L18	LLyMac1	Bocak et al. 2008
Lycidae	Lycinae	*Thonalmus sinuaticostis*	Montserrat	DQ181093	DQ181167	DQ181019	DQ181241	UPOL 000594	LLyThos	Bocak et al. 2008
Lycidae	Lycinae	*Lyponia nigrohumeralis*	China	DQ181048	DQ181122	DQ180974	DQ181196	UPOL 000L17	LLyLyp1	Bocak et al. 2008
Lycidae	Lycinae	*Lycus* sp.	South Africa	DQ181039	DQ181113	DQ180965	DQ181187	UPOL 000L03	LLyLycu	Bocak et al. 2008
Lycidae	Lycinae	*Metriorrhynchus lineatus*	Malaysia	DQ181040	DQ181114	DQ180966	DQ181188	UPOL 000L05	LLyMet3	Bocak et al. 2008
Lampyridae	Ototretinae	*Lamellipalpus pacholatkoi*	India	KF625664	KF626254	KF625955	KF625363	UPOL RK0379	R379LAM	Kundrata et al. 2014
Lampyridae		gen. sp.	Philippines	KF625643	KF626242	KF625934	KF625342	UPOL RK0099	R099LAM	Kundrata et al. 2014
Lampyridae		gen. sp.	French Guyana	KF625661	KF626252	KF625952	KF625360	UPOL RK0375	R375LAM	Kundrata et al. 2014
Lampyridae		gen. sp.	Japan	KF625663	KF626253	KF625954	KF625362	UPOL RK0378	R378LAM	Kundrata et al. 2014
Lampyridae		gen. sp.	South Africa	KF625665	KF626255	KF625956	KF625364	UPOL RK0380	R380LAM	Kundrata et al. 2014
Lampyridae		gen. sp.	Philippines	KF625670	KF626259	KF625961	KF625369	UPOL RK0385	R385LAM	Kundrata et al. 2014
Lampyridae		gen. sp.	Panama	KF625671	KF626260	KF625962	KF625370	UPOL RK0386	R386LAM	Kundrata et al. 2014
Lampyridae		gen. sp.	Indonesia	KF625678	KF626265	KF625969	KF625377	UPOL RK0393	R393LAM	Kundrata et al. 2014
Cantharidae	Cantharinae	*Rhagonycha lignosa*	United Kingdom	AF451939	DQ198770	DQ198687	DQ198610	BMNH 679176	canRhLi	Bocakova et al. 2007

Cantharidae	Cantharinae	*Habronychus* sp.	Japan	KF625625	KF626227	KF625917	KF625324	UPOL 001311	1311CAN	Kundrata et al. 2014
Cantharidae	Silinae	*Laemoglyptus* sp.	Indonesia	KF625601	KF626203	KF625893	KF625301	UPOL 001287	1287CAN	Kundrata et al. 2014
Cantharidae	Silinae	*Asiosilis* sp.	Indonesia	DQ100530	DQ198773	DQ198690	DQ198613	UPOL 000M13	canAsis	Bocakova et al. 2007
Cantharidae	Silinae	gen. sp.	Indonesia	KF625608	KF626210	KF625900	KF625307	UPOL 001294	1294CAN	Kundrata et al. 2014
Cantharidae	Silinae	gen. sp.	Ethiopia	KF625588	KF626190	KF625883	KF625288	UPOL RK0154	R154CAN	Kundrata et al. 2014
Cantharidae	Chauliognathinae	*Chauliognathus* sp.	USA	KF625600	KF626202	KF625892	KF625300	UPOL 001250	1250CAN	Kundrata et al. 2014
Cantharidae	Chauliognathinae	*Ichthyurus* sp.	Indonesia	DQ100531	DQ198774	DQ198691	DQ198614	UPOL 000M12	canIchs	Bocakova et al. 2007
Cantharidae	Chauliognathinae	*Trypherus mutilatus*	Japan	KF625622	KF626224	KF625914	KF625321	UPOL 001308	1308CAN	Kundrata et al. 2014
Cantharidae	Chauliognathinae	gen. sp.	Philippines	KF625594	KF626196	KF625888	KF625294	UPOL RK0179	R179CAN	Kundrata et al. 2014
Cantharidae	Malthininae	*Malthinus* sp.	Czech Republic	KF625627	KF626229	KF625919	KF625326	UPOL 001313	1313CAN	Kundrata et al. 2014
Cantharidae	Malthininae	*Malthodes* sp.	France	DQ100532	DQ198776	DQ198693	DQ198616	UPOL 000M20	canMas1	Bocakova et al. 2007
Cantharidae	Malthininae	*Inmalthodes* sp.	Indonesia	KF625603	KF626205	KF625895	KF625303	UPOL 001289	1289CAN	Kundrata et al. 2014
Cantharidae	Malthininae	gen. sp.	Cameroon	KF625595	KF626197	KF625889	KF625295	UPOL RK0180	R180CAN	Kundrata et al. 2014
Omalisidae	Thilmaninae	*Pseudeuanoma* sp.	Greece	HQ333832	KF626300	HQ333738	HQ334011	UPOL RK0079	R079OMAp	Kundrata Bocak 2011
Omalisidae	Omalisinae	*Omalisus fontisbellaquei*	Czech Republic	AF451948	DQ198749	DQ198658	DQ198580	UPOL 000377	omaOmal	Bocakova et al. 2007
Omalisidae	Paradrilinae	*Paradrilus opacus*	Spain	KJ909284	KJ909285	N	KJ909287	UPOL RK0626	R626OMA	Kundrata et al. 2015
Rhagophthalmidae		*Rhagophthalmus* sp.	India	DQ100508	DQ198756	DQ198665	DQ198587	UPOL 000155	pheRha1	Bocakova et al. 2007
Rhagophthalmidae		*Mimoochotyra* sp.	Malaysia	DQ100505	DQ198753	DQ198662	DQ198584	UPOL 000M30	pheMimo	Bocakova et al. 2007
Rhagophthalmidae		*Bicladodrilus* sp.	China	DQ100507	DQ198755	DQ198664	DQ198586	UPOL 000M35	pheBicl	Bocakova et al. 2007
Rhagophthalmidae		gen. sp.	Indonesia	KF625717	KF626305	KF626005	KF625410	UPOL 001359	1359PHE	Kundrata et al. 2014
Phengodidae		gen. sp.	Panama	KF625723	KF626310	KF626009	KF625416	UPOL RK0366	R366PHE	Kundrata et al. 2014
Phengodidae		gen. sp.	Belize	KF625719	KF626306	KF626007	KF625412	UPOL RK0361	R361PHE	Kundrata et al. 2014
Phengodidae		gen. sp.	Panama	KF625721	KF626308	KF626008	KF625414	UPOL RK0364	R364PHE	Kundrata et al. 2014
Phengodidae		gen. sp.	Panama	KF625728	KF626315	KF626014	KF625421	UPOL 001245	1245PHE	Kundrata et al. 2014
Phengodidae		gen. sp.	Panama	KF625730	KF626317	KF626016	KF625423	UPOL 001247	1247PHE	Kundrata et al. 2014
Phengodidae		gen. sp.	Panama	KF625736	KF626320	KF626020	KF625430	UPOL 001351	1351PHE	Kundrata et al. 2014
Elateridae	Agrypninae	*Agrypnus murinus*	Slovakia	AF451943	DQ198735	DQ198645	DQ198567	UPOL 001049	elaAGRam	Bocakova et al. 2007
Elateridae	Agrypninae	*Adelocera* sp.	Japan	HQ333778	HQ333873	HQ333694	HQ333961	UPOL RK0025	R025EAgr	Kundrata Bocak 2011
Elateridae	Agrypninae	*Drasterius bimaculatus*	Slovakia	HQ333793	HQ333888	HQ333704	HQ333975	UPOL RK0040	R040EAgr	Kundrata Bocak 2011
Elateridae	Agrypninae	*Pyrophorus* sp.	French Guyana	KF625742	KF626325	KF626030	KF625436	UPOL RK0213	R213EAgr	Kundrata et al. 2014
Elateridae	Agrypninae	*Cryptalaus* sp.	Malaysia	HQ333834	HQ333926	HQ333740	HQ334014	UPOL RK0082	R082EAgr	Kundrata Bocak 2011
Elateridae	Agrypninae	*Drilus flavescens*	Malta	DQ100501	DQ198748	DQ198657	DQ198579	UPOL 001046	driDril	Bocakova et al. 2007
Elateridae	Dendrometrinae	*Nothodes parvulus*	Slovakia	HQ333763	HQ333858	HQ333681	KF625443	UPOL RK0010	R010EDen	Kundrata Bocak 2011
Elateridae	Dendrometrinae	*Athous vittatus*	Czech Republic	HQ333755	HQ333850	HQ333674	HQ333939	UPOL RK0002	R002EDen	Kundrata Bocak 2011
Elateridae	Dendrometrinae	*Pheletes quercus*	Czech Republic	HQ333775	HQ333870	HQ333692	HQ333958	UPOL RK0022	R022EDen	Kundrata Bocak 2011
Elateridae	Dendrometrinae	*Cidnopus pilosus*	Slovakia	HQ333792	HQ333887	HQ333703	HQ333974	UPOL RK0039	R039EDen	Kundrata Bocak 2011
Elateridae	Dendrometrinae	*Selatosomus latus*	Czech Republic	HQ333774	HQ333869	HQ333691	HQ333957	UPOL RK0021	R021EDen	Kundrata Bocak 2011
Elateridae	Dendrometrinae	*Denticollis linearis*	Czech Republic	DQ100498	DQ198741	DQ198651	DQ198573	UPOL 000M25	elaDENdl	Bocakova et al. 2007
Elateridae	Elaterinae	*Cebrio* sp.	Italy	KF625745	KF626329	KF626040	KF625440	UPOL RK0142	R142ECeb	Kundrata et al. 2014
Elateridae	Elaterinae	*Elater* sp.	Japan	HQ333766	HQ333861	HQ333683	HQ333949	UPOL RK0013	R013EEla	Kundrata Bocak 2011
Elateridae	Elaterinae	*Tomicephalus* sp.	Panama	HQ333750	HQ333845	HQ333669	HQ333935	UPOL 001420	1420EEla	Kundrata Bocak 2011
Elateridae	Elaterinae	*Anoplischius* sp.	Panama	HQ333745	HQ333840	HQ333664	HQ333930	UPOL 001415	1415EEla	Kundrata Bocak 2011
Elateridae	Elaterinae	*Agriotes acuminatus*	Czech Republic	HQ333756	HQ333851	HQ333675	HQ333940	UPOL RK0003	R003EEla	Kundrata Bocak 2011
Elateridae	Elaterinae	*Melanotus villosus*	Czech Republic	HQ333754	HQ333849	HQ333673	HQ333938	UPOL RK0001	R001EEla	Kundrata Bocak 2011
Elateridae	Elaterinae	*Priopus ornatus*	Laos	HQ333785	HQ333880	HQ333698	HQ333967	UPOL RK0032	R032EEla	Kundrata Bocak 2011
Elateridae	Morostomatinae	*Diplophoenicus* sp.	Madagascar	KF625753	KF626335	KF626050	KF625454	UPOL RK0145	R145E	Kundrata et al. 2014

References for Table S3

Bocak, L., Bocakova, M., Hunt, T. & Vogler, A. P. Multiple ancient origins of neoteny in Lycidae (Coleoptera): consequences for ecology and macroevolution. *Proc. R. Soc. B* **275**, 2015–2023 (2008).

Bocak, L., Kundrata, R., Andújar-Fernández, C. & Vogler, A. P. The discovery of Iberobaeniidae (Coleoptera: Elateroidea), a new family of beetles from Spain, with immatures detected by environmental DNA sequencing. *Proc. R. Soc. B* **283**, 20152350 (2016).

Bocakova, M., Bocak, L., Hunt, T., Teraväinen, M. & Vogler, A. P. Molecular phylogenetics of Elateriformia (Coleoptera): evolution of bioluminescence and neoteny. *Cladistics* **23**, 477–496 (2007).

Evans, A. M., McKenna, D. D., Bellamy, C. L. & Farrell, B. D. Large-scale molecular phylogeny of metallic wood-boring beetles (Coleoptera: Buprestoidea) provides new insights into relationships and reveals multiple evolutionary origins of the larval leaf-mining habit. *Syst. Entomol.* **40**, 385–400 (2015).

Hunt, T. et al. A comprehensive phylogeny of beetles reveals the evolutionary origins of a superradiation. *Science* **318**, 1913–1916 (2007).

Kundrata, R. & Bocak, L. The phylogeny and limits of Elateridae (Insecta, Coleoptera): is there a common tendency of click beetles to soft-bodiedness and neoteny? *Zool. Scr.* **40**, 364–378 (2011).

Kundrata, R., Bocakova, M. & Bocak, L. The phylogenetic position of Artematopodidae (Coleoptera: Elateroidea), with description of the first two *Eurypogon* species from China. *Contrib. Zool.* **82**, 199–208 (2013).

Kundrata, R., Bocakova, M. & Bocak, L. The comprehensive phylogeny of the superfamily Elateroidea (Coleoptera: Elateriformia). *Mol. Phylogenet. Evol.* **76**, 162–171 (2014).

Kundrata, R., Jäch, M. A. & Bocak, L. Molecular phylogeny of the Byrrhoidea-Buprestoidea complex (Coleoptera, Elateriformia). *Zool. Scr.* **46**, 150–164 (2017).

Table S4. Results of the Xia's nucleotide substitution saturation test in DAMBE, based on simulations with 32 operational taxonomic units (10,000 replicates). Analysis performed on fully resolved sites only. Iss - index of substitution saturation; Iss.c^S - critical value for symmetrical tree topology; Iss.c^A - critical value for extremely asymmetrical tree topology; T - T value; DF - degrees of freedom; P^S, P^A - probability that Iss is significantly different from the critical value (Iss.c^S or Iss.c^A, respectively); Pinv - proportion of invariable sites.

Marker /codon pos.	Iss	Iss.c^S	T	DF	P^S	Iss.c^A	T	DF	P^A	Pinv
18S rRNA	0.047	0.705	56.653	408	0.000	0.378	28.521	408	0.000	0.217
28S rRNA	0.158	0.726	15.850	133	0.000	0.445	8.005	133	0.000	0.054
rrnL mtDNA	0.253	0.688	12.141	200	0.000	0.371	3.294	200	0.001	0.044
cox1 mtDNA/1st	0.189	0.727	12.331	132	0.000	0.447	5.904	132	0.000	0.060
cox1 mtDNA/2nd	0.064	0.724	28.896	126	0.000	0.441	16.507	126	0.000	0.120
cox1 mtDNA/3rd	0.687	0.725	1.097	142	0.274	0.443	6.919	142	0.000	0.000

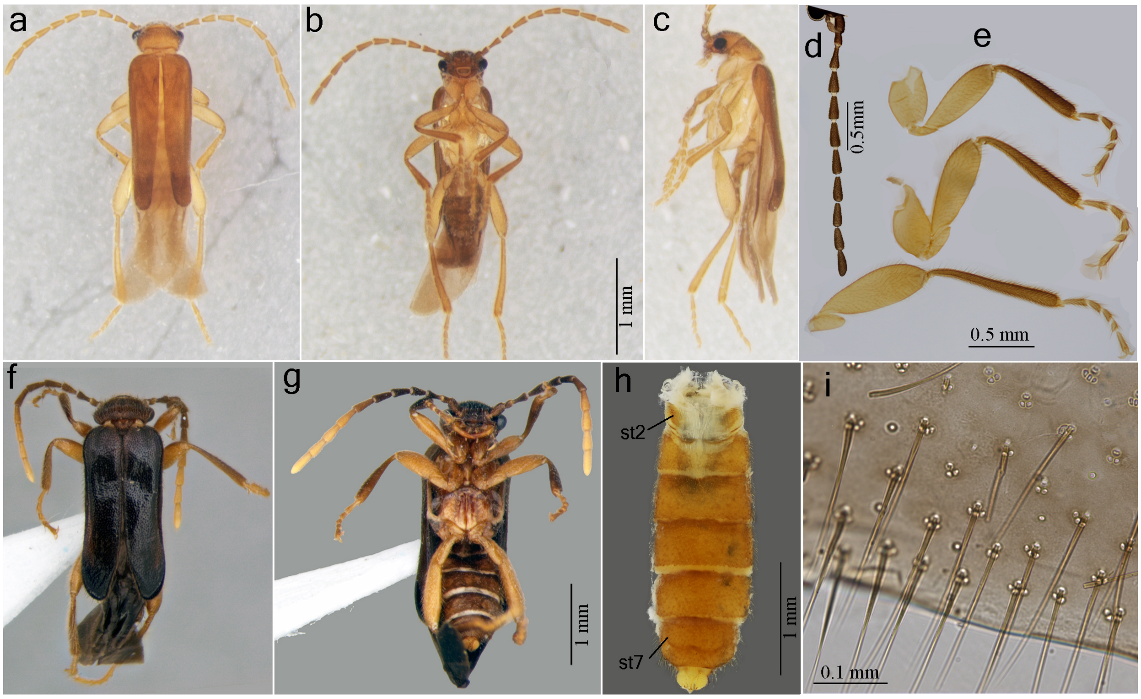

Supplementary Figure S1. Adult male habitus and morphology of *Jurasai* gen. nov. (**a–e**) *J. itajubense* sp. nov., male: (**a**, **b**, **c**) habitus (dorsal, ventral and lateral view, respectively); (**d**) right antenna (dorsal view); (**e**) left anterior, middle and posterior legs (from top to bottom, ventral view). (**f–i**) *J. digitusdei* sp. nov., male: (**f**, **g**) habitus (dorsal and ventral, respectively); (**h**) abdomen (ventral view); (**i**) detail of the abdominal sternite VII. Abbreviations: st2, abdominal sternite II; st7, abdominal sternite VII.

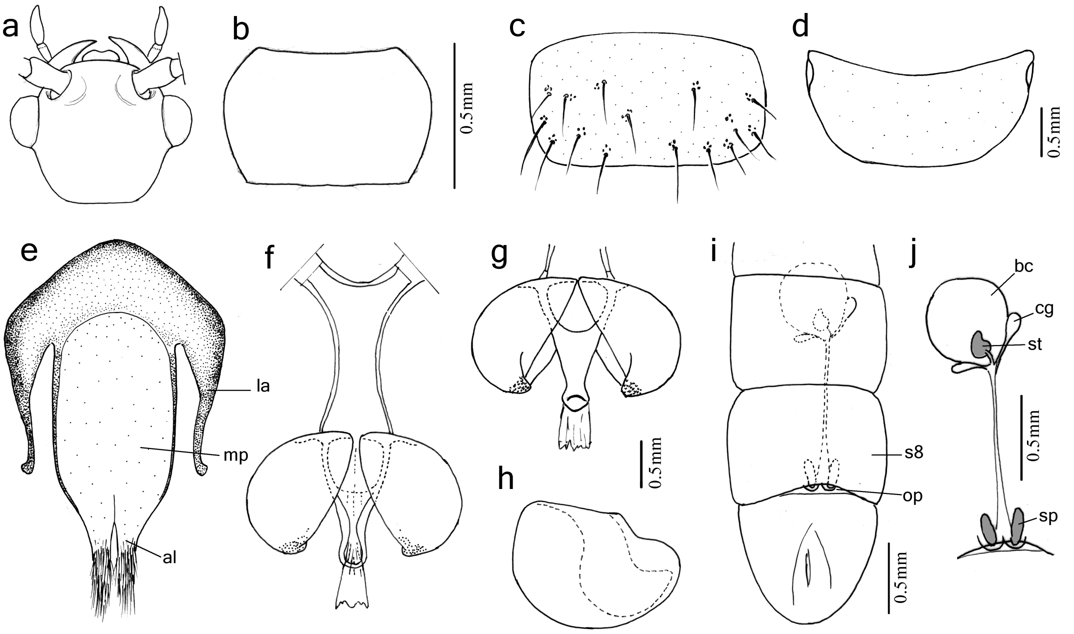

Supplementary Figure S2. Adult morphology of *Jurasai itajubense* sp. nov. Male: (**a**) head (dorsal view); (**b**) pronotum; (**c**) abdominal tergite VIII; (**d**) abdominal sternite VIII; (**e**) abdominal sternite IX; (**f**) aedeagus (dorsal view); (**g**, **h**) phallus and parameres (ventral and lateral view, respectively). Female: (**i**) abdominal segments VII–X and genital tract (ventral view); (**j**) genital tract (ventral view). Abbreviations: a, lateral arm; al, apical lobe; bc, bursa copulatrix; cg, colleterial glands; mp, median part; op, ooporus; s8, abdominal segment VIII; sp, supporting plates; st, spermatheca.

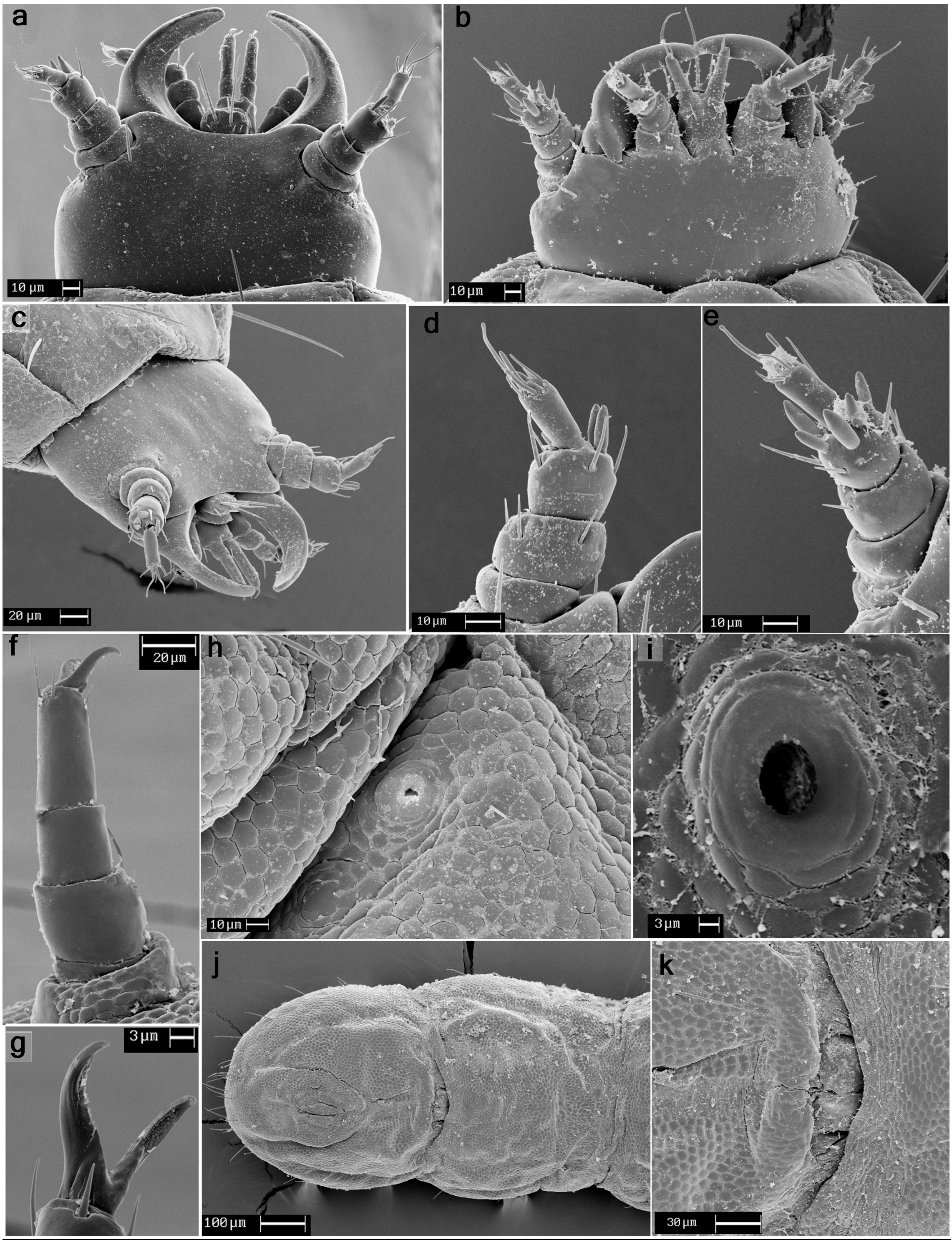

Supplementary Figure S3. Adult female morphology of *Jurasai itajubense* sp. nov. (**a**–**c**) head (dorsal, ventral and laterodorsal, respectively); (**d)** left antenna (dorsal view); (**e**) right antenna (ventral view); (**f**) posterior leg (lateral view); (**g**) claws; (**h, i)** abdominal spiracle; (**j**) abdominal segments VIII–X (ventral view); (**k**) ooporus.

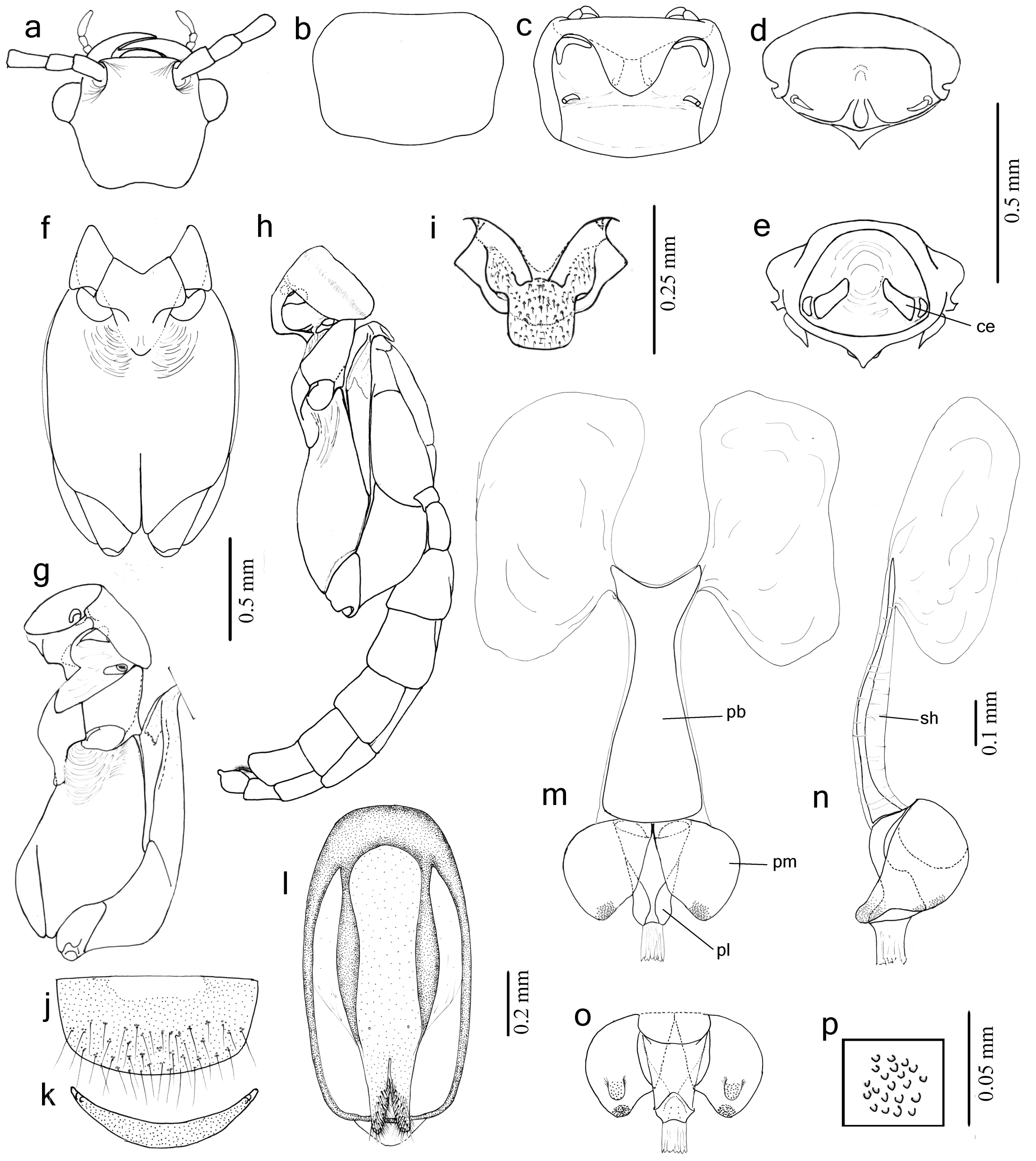

Supplementary Figure S4. Adult male morphology of *Jurasai digitusdei* sp. nov. (**a**) head (dorsal view); (**b–e**) prothorax (dorsal, ventral, anterior and posterior view, respectively); (**f**) pterothorax (ventral view); (**g**) thorax (lateroventral view); (**h**) thorax and abdomen (lateral view); (**i**) mesoscutum and scutellum; (**j**) abdominal tergite VIII; (**k**) abdominal sternite VIII; (**l**) abdominal segment IX (ventral view); (**m**, **n**) aedeagus (dorsal and lateral view, respectively); (**o**) phallus and parameres (ventral view); (**p**) asperities of parameres. Abbreviations: ce, cervical sclerites; pb, phallobase; pl, phallus; pm, parameres; sh, phallobase sheath.

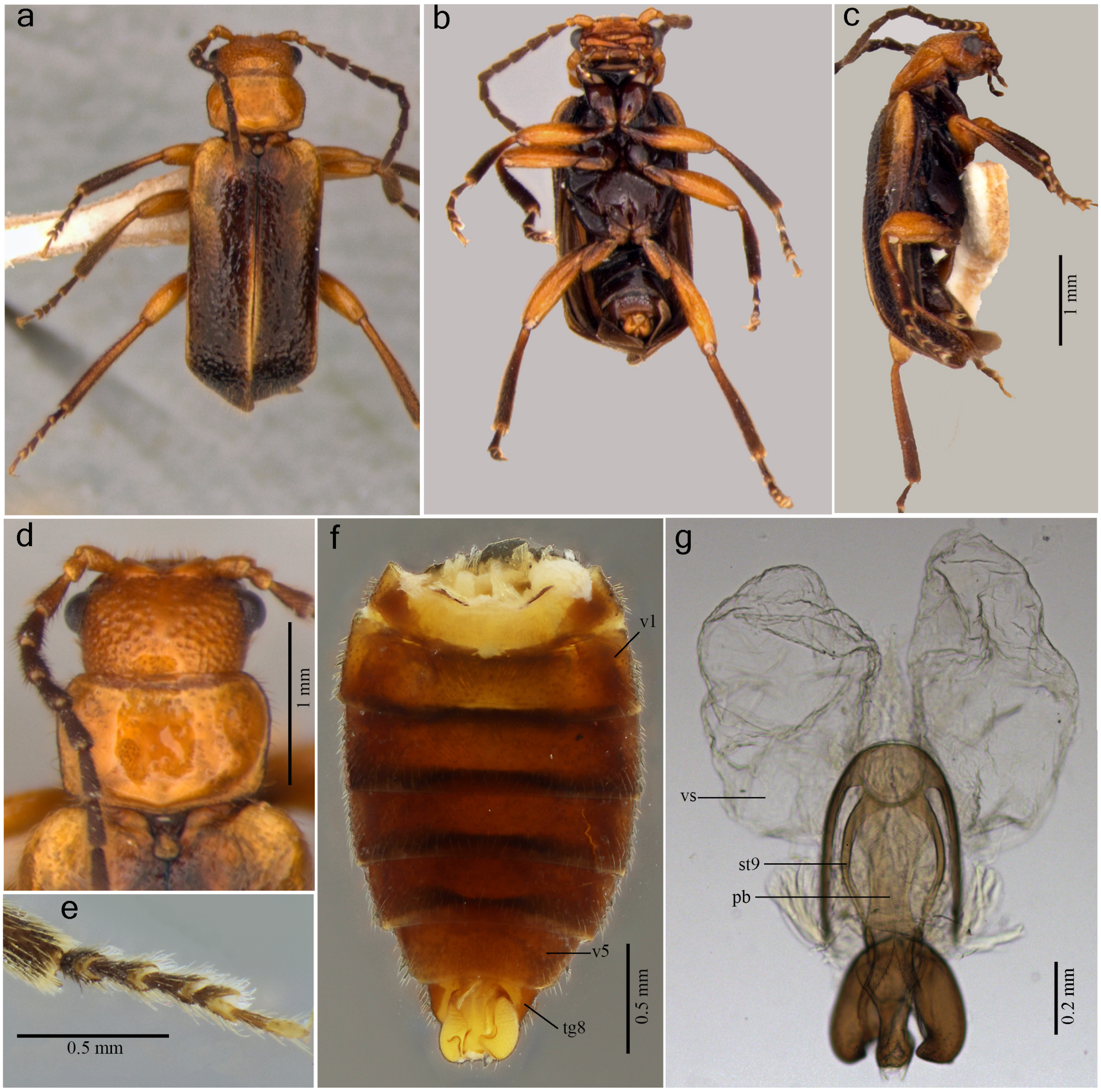

Supplementary Figure S5. Adult male habitus and morphology of *Tujamita plenalatum* sp. nov. (**a**–**c**) habitus (dorsal, ventral and lateral view, respectively), (**d**) head and prothorax (dorsal view); (**e**) left metatarsus (dorsal view); (**f**) abdomen (ventral view) (**g**) aedeagus and abdominal segment IX (ventral view). Abbreviations: pb, phallobase; st9, abdominal sternite IX; tg8, abdominal tergite VIII; v1, abdominal ventrite 1; v5, abdominal ventrite 5; vs, vesicle of phallobase sheath.

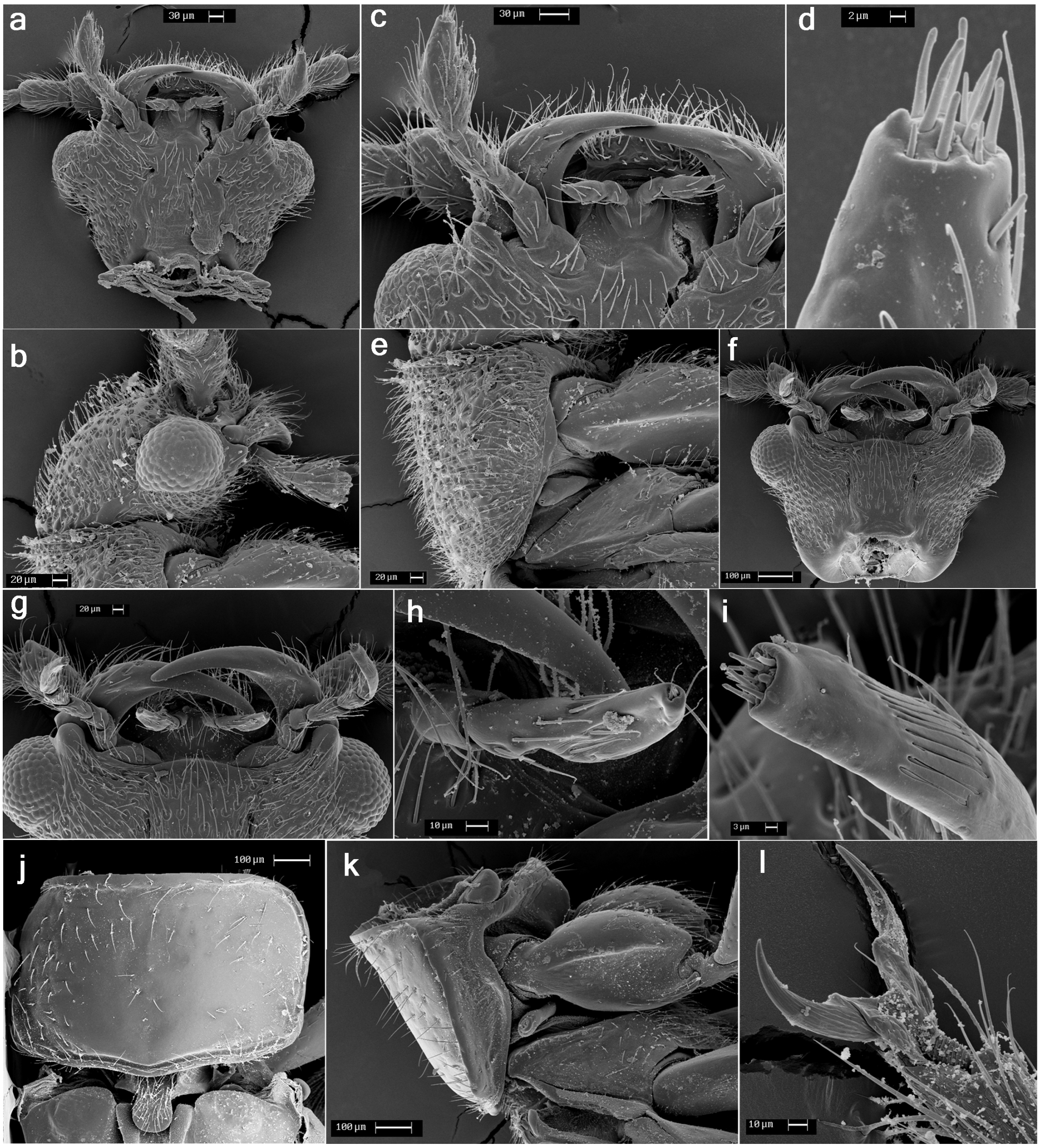

Supplementary Figure S6. Adult male morphology of Jurasaidae. (**a–e**) *Jurasai itajubense* sp. nov.: (**a, b**) head (ventral and lateral view, respectively); (**c**) detail of mouthparts (ventral view); (**d**) apex of maxillary palpus; (**e**) prothorax (lateral view). (**f–i**) *J. digitusdei* sp. nov.: (**f**) head (ventral view); (**g**) mouthparts (ventral view); (**h**) labial palpus; (**i**) apical maxillary palpomere. (**j–l**) *Tujamita plenalatum* sp. nov.: (**j**) pronotum and scutellum; (**k**) pro- and mesothorax (lateral view); (**l**) claws.

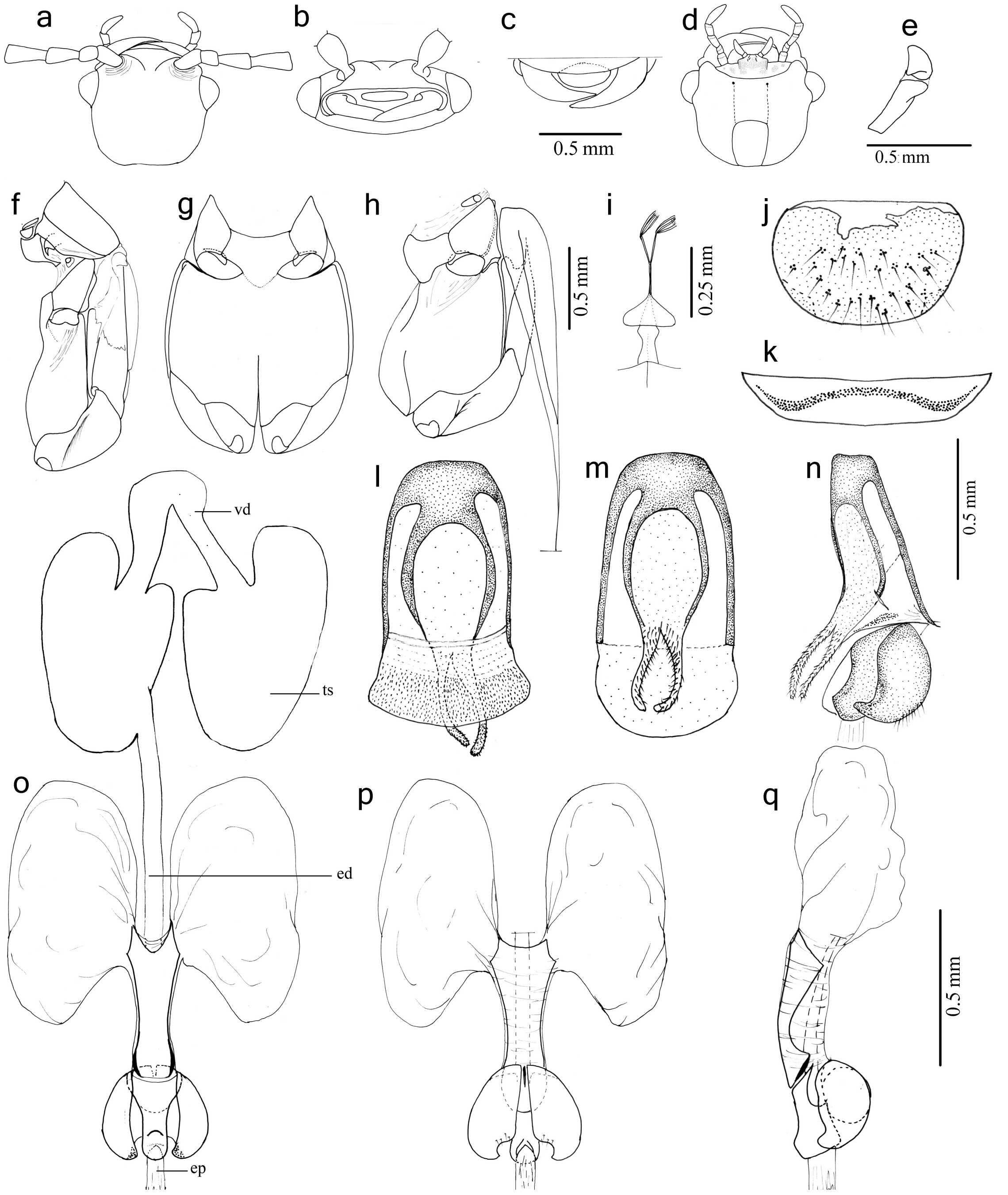

Supplementary Figure S7. Adult male morphology of *Tujamita plenalatum* sp. nov. (**a**–**b**) head (dorsal and frontal view, respectively); (**c**) mandibles and labrum (dorsal view); (**d**) head (ventral view), (**e**) right cervical sclerites (ventral view); (**f**) thorax (lateral view); (**g**) pterothorax (ventral view); (**h**) pterothorax and epipleura (lateroventral view); (**i**) metendosternite (dorsal view); (**j**) abdominal tergite VIII; (**k**) abdominal sternite VIII; (**l**, **m**) abdominal segment IX (dorsal and ventral view, respectively); (**n**) abdominal segment IX and aedeagus (lateroventral view); (**o**) aedeagus and reproductive tract (ventral view); (**p, q**) aedeagus (dorsal and lateral view, respectively). Abbreviations: ed, ejaculatory duct; ep, endophallus; ts, testis; vd, vas deferens.

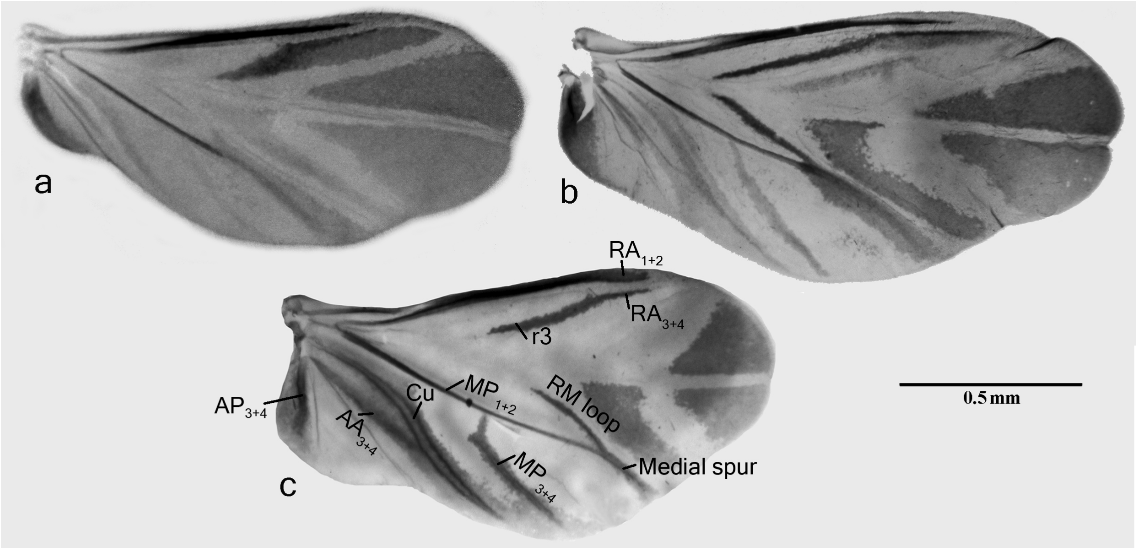

Supplementary Figure S8. Hind wings of Jurasaidae. (**a)** *Jurasai itajubense* sp. nov.; **b**, *J. digitusdei* sp. nov.; **c**, *Tujamita plenalatum* sp. nov.

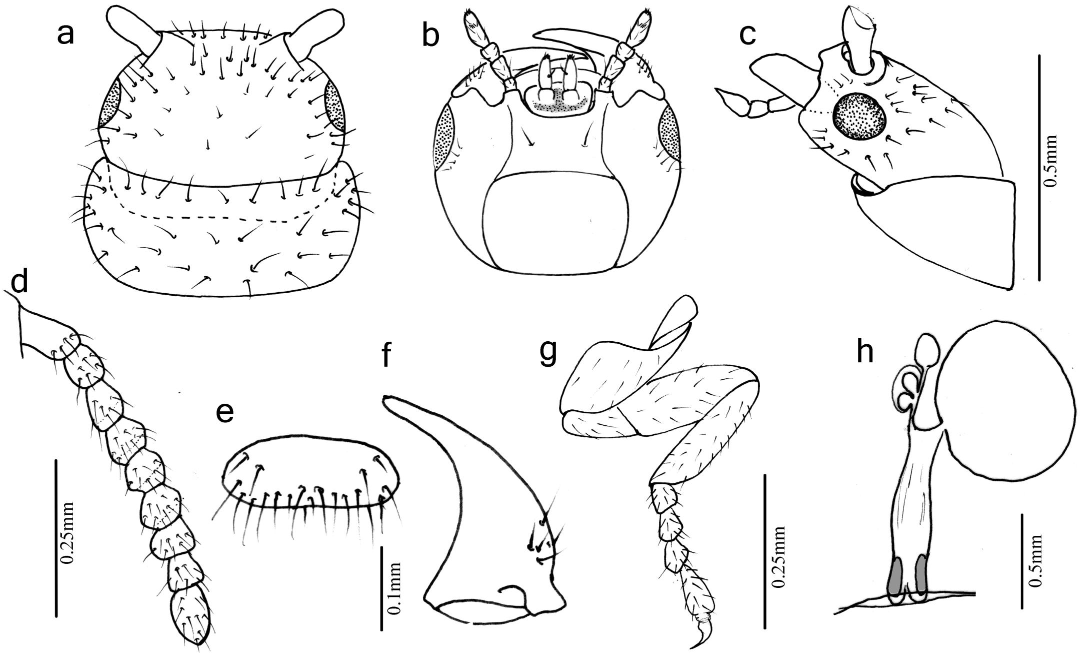

Supplementary Figure S9. Adult female morphology of *Tujamita plenalatum* sp. nov. (**a**) head and prothorax (dorsal view); (**b**) head (ventral view); (**c**) head and prothorax (lateral view); (**d**) right antenna (dorsal view); (**e**) labrum; (**f**) mandible (dorsal view); (**g**) left anterior leg (ventral view); (**h**) genital tract (ventral view).

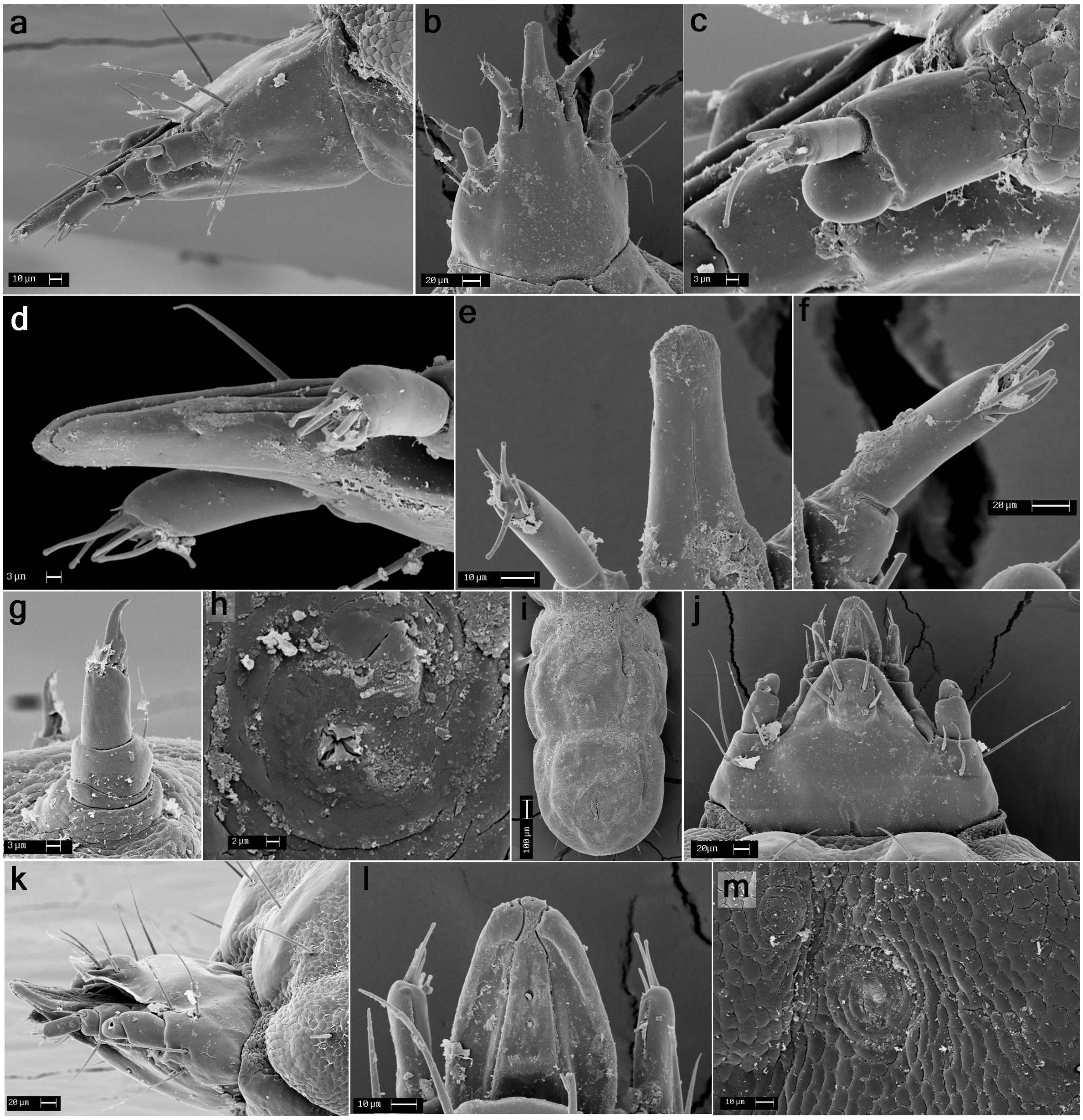

Supplementary Figure S10. Mature larval morphology of Jurasaidae. (**a–i**) *Jurasai itajubense* sp. nov.: (**a, b**) head (lateral and ventral view, respectively); (**c**) antenna (lateral view); (**d, e**) beak-like mouthpart (lateral and ventral view, respectively); (**f**) left maxillary palpus (ventral view); (**g**) right anterior leg (lateral view); (**h**) abdominal spiracle; (**i**) abdominal segments VIII–X (ventral view). **(j–m**) *Tujamita plenalatum* sp. nov.: (**j, k**) head (dorsal and lateral view, respectively); (**l**) beak-like mouthpart (dorsal view); (**m)** detail of tegument and abdominal spiracle.

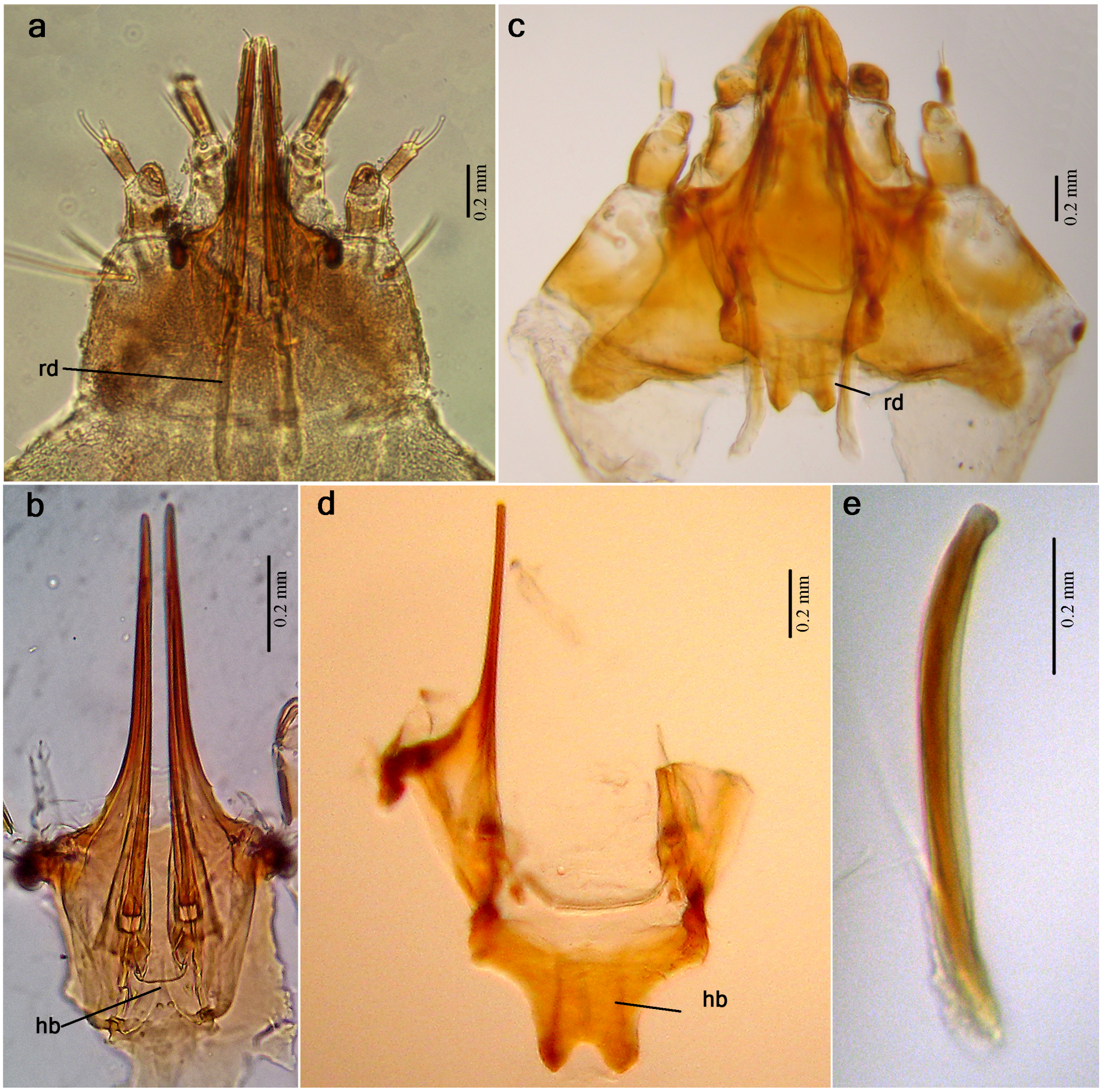

Supplementary Figure S11. Mature larval head morphology of Jurasaidae. (**a, b)** *Jurasai itajubense* sp. nov.: (**a**) head (dorsal view); (**b**) mandibles (ventral view); (**c–e**) *Tujamita plenalatum* sp. nov.: (**c**) head exuvia (dorsal view); **d**, mandibles and hypopharyngeal bracon (ventral view, lacking left mandibular apex); (**e**) right posterior rod (dorsal view). Abbreviations: hb, hypopharyngeal bracon; rd, internal rod.

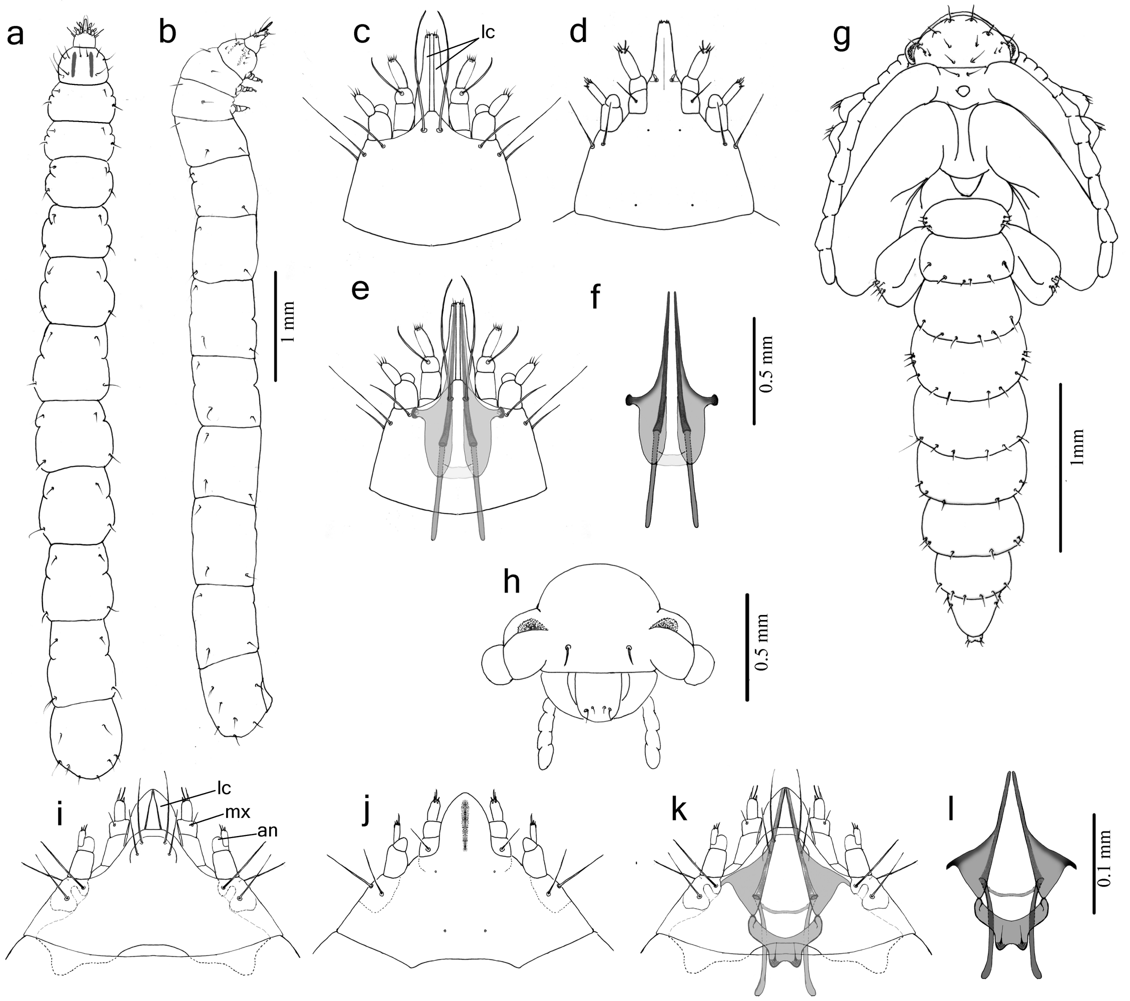

Supplementary Figure S12. Immature morphology of Jurasaidae. (**a–h**) *Jurasai itajubense* sp. nov.: (**a–f)** larva: (**a, b**) habitus (dorsal and lateral view, respectively); (**c, d**) head (dorsal and ventral, respectively); (**e**) head with mandibles and endoskeleton (dorsal view); (**f**) mandibles and endoskeleton (dorsal view); (**g, h**) pupa: (**g)** habitus (dorsal view); (**h**) head (frontal view). (**i–l**) *Tujamita plenalatum* sp. nov., larva: (**i, j**) head (dorsal and ventral view, respectively); (**k)** head with mandibles and endoskeleton (dorsal view); (**l**) mandibles and endoskeleton (dorsal view). Abbreviations: an, antenna; lc, labial channel; mx, maxillary palpus.

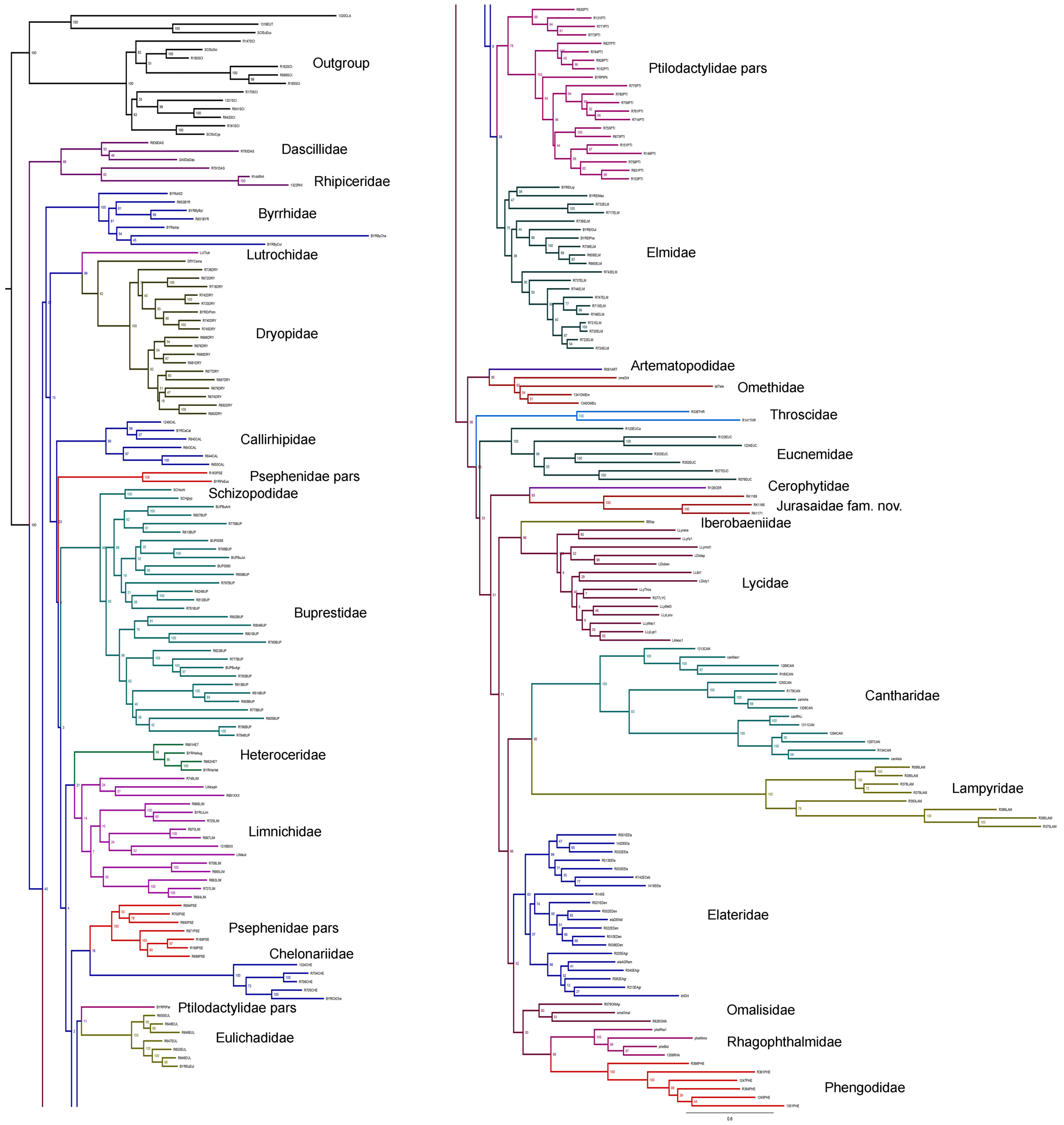

Supplementary Figure S13. Phylogenetic position of Jurasaidae within Elateriformia. Based on the maximum likelihood analysis performed on the four-gene 251-taxa dataset aligned using the Mafft algorithm. Numbers at nodes indicate bootstrap support values.

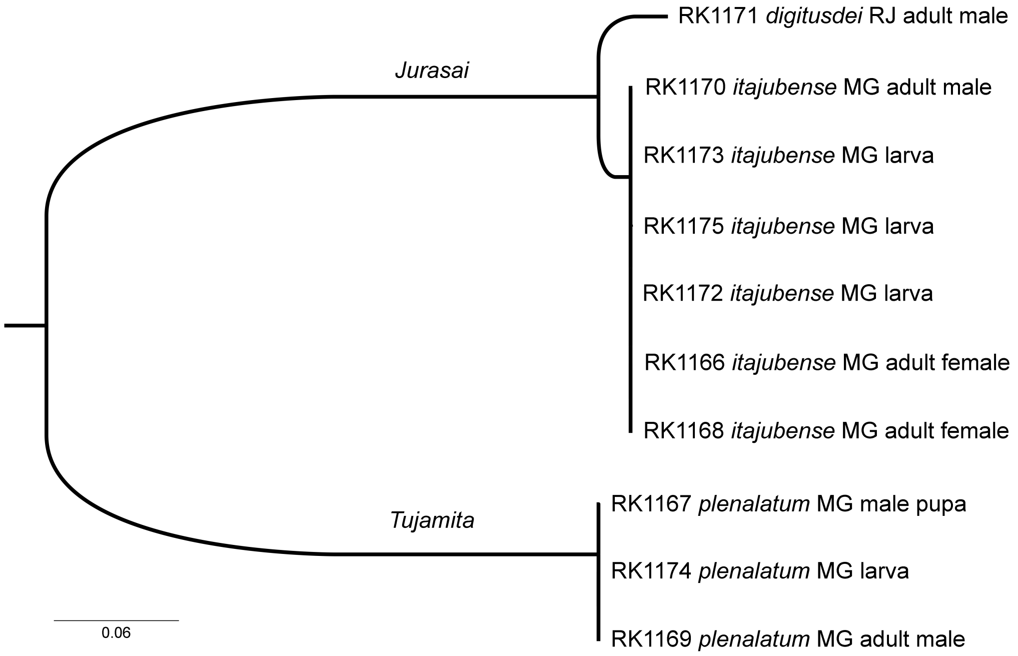

Supplementary Figure S14. DNA-based association of different sexes and semaphoronts in Jurasaidae. Based on the maximum likelihood analysis performed on the four-gene 10-taxa dataset aligned using the Mafft algorithm. Abbreviations: MG, Minas Gerais; RJ, Rio de Janeiro.
